# Updated classification of epileptic seizures: Position paper of the International League Against Epilepsy

**DOI:** 10.1111/epi.18338

**Published:** 2025-04-23

**Authors:** Sándor Beniczky, Eugen Trinka, Elaine Wirrell, Fatema Abdulla, Raidah Al Baradie, Mario Alonso Vanegas, Stéphane Auvin, Mamta Bhushan Singh, Hal Blumenfeld, Alicia Bogacz Fressola, Roberto Caraballo, Mar Carreno, Fernando Cendes, Augustina Charway, Mark Cook, Dana Craiu, Birinus Ezeala‐Adikaibe, Birgit Frauscher, Jacqueline French, M. V. Gule, Norimichi Higurashi, Akio Ikeda, Floor E. Jansen, Barbara Jobst, Philippe Kahane, Nirmeen Kishk, Ching Soong Khoo, Kollencheri Puthenveettil Vinayan, Lieven Lagae, Kheng‐Seang Lim, Angelica Lizcano, Aileen McGonigal, Katerina Tanya Perez‐Gosiengfiao, Philippe Ryvlin, Nicola Specchio, Michael R. Sperling, Hermann Stefan, William Tatum, Manjari Tripathi, Elza Márcia Yacubian, Samuel Wiebe, Jo Wilmshurst, Dong Zhou, J. Helen Cross

**Affiliations:** ^1^ Department of Neurology Aarhus University Hospital Aarhus Denmark; ^2^ Department of Clinical Medicine Aarhus University Aarhus Denmark; ^3^ Department of Clinical Neurophysiology Danish Epilepsy Center Dianalund Denmark; ^4^ Department of Neurology, Center for Cognitive Neuroscience Christian Doppler University Hospital, Paracelsus Medical University Salzburg Austria; ^5^ Neuroscience Institute, Center for Cognitive Neuroscience Christian Doppler University Hospital, Paracelsus Medical University Salzburg Austria; ^6^ Medical Informatics, and Technology, Institute of Public Health, Medical Decision Making, and Health Technology Assessment University for Health Sciences Hall in Tyrol Austria; ^7^ Division of Child and Adolescent Neurology and Epilepsy, Department of Neurology Mayo Clinic Rochester Minnesota USA; ^8^ Department of Clinical Neurosciences Salmaniya Medical Complex, Government Hospitals Manama Kingdom of Bahrain; ^9^ King Fahd Specialist Hospital Dammam Al Hofuf Saudi Arabia; ^10^ International Epilepsy Center HMG Coyoacán Mexico City Mexico; ^11^ Pediatric Neurology Department, CRMR Epilepsies Rares APHP, Robert Debré University Hospital Paris France; ^12^ INSERM NeuroDiderot, Université Paris Cité Paris France; ^13^ Institut Universitaire de France Paris France; ^14^ Department of Neurology All India Institute of Medical Sciences New Delhi India; ^15^ Department of Neurology, Neuroscience, and Neurosurgery Yale University School of Medicine New Haven Connecticut USA; ^16^ Neurological Institute Universidad de la República Montevideo Uruguay; ^17^ Hospital de Pediatría Juan P. Garrahan Buenos Aires Argentina; ^18^ Clinical Institute of Neurosciences University Hospital Clinic of Barcelona Barcelona Spain; ^19^ Epilepsy Unit, Neurology Department University Hospital Clinic of Barcelona Barcelona Spain; ^20^ Department of Neurology, School of Medical Sciences University of Campinas São Paulo Brazil; ^21^ Brazilian Institute of Neuroscience and Neurotechnology São Paulo Brazil; ^22^ 37 Military Hospital Accra Ghana; ^23^ Faculties of Engineering and Information Technology, and Medicine, Dentistry, and Health Sciences University of Melbourne Melbourne Victoria Australia; ^24^ Pediatric Neurology Discipline, Neuroscience Department “Carol Davila” University of Medicine Bucharest Romania; ^25^ Center of Expertise of Rare Pediatric Neurological Disorders Al Obregia Clinical Hospital Bucharest Romania; ^26^ Department of Medicine University of Nigeria Teaching Hospital, Ituku/Ozalla Enugu Nigeria; ^27^ Neurology Unit Memfys Hospital Enugu Nigeria; ^28^ Department of Neurology Duke University School of Medicine Durham North Carolina USA; ^29^ Department of Biomedical Engineering Duke Pratt School of Engineering Durham North Carolina USA; ^30^ Department of Neurology New York University New York New York USA; ^31^ Division of Neurology, Department of Medicine Neuroscience Institute and Groote Schuur Hospital, University of Cape Town Cape Town South Africa; ^32^ Centre for Global Epilepsy University of Oxford Oxford UK; ^33^ Musashi‐Kosugi Pediatrics & Epilepsy Clinic and Department of Pediatrics Jikei University School of Medicine Minato‐ku Japan; ^34^ Department of Epilepsy, Movement Disorders, and Physiology Kyoto University Graduate School of Medicine Shogoin Kyoto Japan; ^35^ Department of Child Neurology, Brain Center University Medical Center Utrecht the Netherlands; ^36^ Department of Neurology, Dartmouth‐Hitchcock Health Geisel School of Medicine at Dartmouth Lebanon New Hampshire USA; ^37^ Neurology Department CHU Grenoble Alpes, Université Grenoble Alpes, INSERM, U1216, Grenoble Institut Neurosciences Grenoble France; ^38^ Department of Neurology, School of Medicine, Kasralainy Hospital Cairo University Cairo Egypt; ^39^ Faculty of Medicine Universiti Kebangsaan Malaysia Kuala Lumpur Malaysia; ^40^ Neurology Unit, Department of Medicine Hospital Canselor Tuanku Muhriz Kuala Lumpur Malaysia; ^41^ Centre for Global Epilepsy Wolfson College, University of Oxford Oxford UK; ^42^ Department of Pediatric Neurology and Amrita Advanced Center for Epilepsy Amrita Institute of Medical Sciences Cochin India; ^43^ Pediatric Neurology University of Leuven Leuven Belgium; ^44^ Division of Neurology, Department of Medicine, Faculty of Medicine University of Malaya Kuala Lumpur Malaysia; ^45^ Department of Clinical Neurophysiology and Epilepsy Clinic Neurocentro and Coneuro Pereira Colombia; ^46^ Laboratory of Neuroimmunology Medcare Pereira Colombia; ^47^ Centre for Neurosciences, Mater Hospital Brisbane University of Queensland Brisbane Queensland Australia; ^48^ Department of Neurosciences University of the Philippines–Philippine General Hospital Manila Philippines; ^49^ Department of Neurosciences Makati Medical Center Makati Philippines; ^50^ Institute for Neurological Sciences St. Lukes Medical Center Global City Taguig Philippines; ^51^ Department of Clinical Neurosciences Centre Hospitalier Universitaire Vaudois and Université de Lausanne Lausanne Switzerland; ^52^ Neurology, Epilepsy, and Movement Disorders Unit Bambino Gesù Children’s Hospital, IRCCS, Rome, Italy and University Hospitals KU Leuven Leuven Belgium; ^53^ Department of Neurology, Jefferson Comprehensive Epilepsy Center Thomas Jefferson University Philadelphia Pennsylvania USA; ^54^ Department of Neurology‐Biomagnetism University Hospital Erlangen Germany; ^55^ Department of Neurology Mayo Clinic Jacksonville Florida USA; ^56^ Department of Neurology AIIMS Delhi India; ^57^ Department of Neurology and Neurosurgery Universidade Federal de São Paulo São Paulo Brazil; ^58^ Department of Clinical Neurosciences University of Calgary Calgary Alberta Canada; ^59^ Department of Pediatric Neurology, Red Cross War Memorial Children's Hospital Neuroscience Institute, University of Cape Town Cape Town South Africa; ^60^ Department of Neurology West China Hospital of Sichuan University Chengdu China; ^61^ NIHR BRC Great Ormond Street Institute of Child Health, Great Ormond Street Hospital & Young Epilepsy University College London London UK

**Keywords:** International League Against Epilepsy, seizure classification, update

## Abstract

The International League Against Epilepsy (ILAE) has updated the operational classification of epileptic seizures, building upon the framework established in 2017. This revision, informed by the implementation experience, involved a working group appointed by the ILAE Executive Committee. Comprising 37 members from all ILAE regions, the group utilized a modified Delphi process, requiring a consensus threshold of more than two thirds for any proposal. Following public comments, the Executive Committee appointed seven additional experts to the revision task force to address and incorporate the issues raised, as appropriate. The updated classification maintains four main seizure classes: Focal, Generalized, Unknown (whether focal or generalized), and Unclassified. Taxonomic rules distinguish classifiers, which are considered to reflect biological classes and directly impact clinical management, from descriptors, which indicate other important seizure characteristics. Focal seizures and those of unknown origin are further classified by the patient's state of consciousness (impaired or preserved) during the seizure, defined operationally through clinical assessment of awareness and responsiveness. If the state of consciousness is undetermined, the seizure is classified under the parent term, that is, the main seizure class (focal seizure or seizure of unknown origin). Generalized seizures are grouped into absence seizures, generalized tonic–clonic seizures, and other generalized seizures, now including recognition of negative myoclonus as a seizure type. Seizures are described in the basic version as with or without observable manifestations, whereas an expanded version utilizes the chronological sequence of seizure semiology. This updated classification comprises four main classes and 21 seizure types. Special emphasis was placed on ensuring translatability into languages beyond English. Its aim is to establish a common language for all health care professionals involved in epilepsy care, from resource‐limited areas to highly specialized centers, and to provide accessible terms for patients and caregivers.

**Key points**

- The ILAE has updated the operational classification of epileptic seizures.
- Adjustments were based on experience with the clinical implementation of the classification established in 2017.
- The four main classes are: Focal, Generalized, Unknown (whether focal or generalized), and Unclassified.
- Consciousness is a classifier, and it is operationally defined by awareness and responsiveness.
- Seizures are described as with or without observable manifestations (basic) or by the chronological sequence of semiology (expanded).

## 1 INTRODUCTION

The International League Against Epilepsy (ILAE) operational classification of seizure types was published in 2017.^1^ The paper concluded with a statement suggesting that the application of this classification in the field for a few years would prompt minor revisions and clarifications. The ILAE actively promoted the implementation of the 2017 classification, sparking intense debates within the international epilepsy community.^2^, ^3^, ^4^, ^5^, ^6^

In 2023, the ILAE's Executive Committee appointed a working group assigned to assess the real‐world application of the 2017 seizure classification and recommend adjustments while preserving the fundamental framework of the 2017 classification. The basic organization of the 2017 classification evolved from the original 1981 version^7^ through subsequent modifications. The primary objective remains the establishment of a common language and standardized definitions for clinical practice. Emphasizing flexibility, the classification aims to accommodate diverse clinical settings, including resource‐limited areas and highly specialized centers. Simultaneously, it seeks to offer a clear and robust structure for implementation in research databases and clinical trials.

This seizure classification does not encompass neonatal seizures, which are addressed in a separate position paper.^8^ Additionally, a new definition of acute symptomatic seizures^9^ and the nosology of status epilepticus^10^ have been allocated to other working groups. Notably, this classification specifically encompasses clinical seizures, omitting those events solely identified by electrographic activity.

The working group, appointed by the ILAE's Executive Committee, comprised a diverse and inclusive international representation. The methodology employed three successive steps: first, the identification of strengths and weaknesses within the 2017 classification; second, proposing adjustments and updates; and finally, engaging in an iterative Delphi process to attain a broad consensus. The updated version was made available on the ILAE website for a 2‐month period to receive public comments, subsequently undergoing successive revisions. In parallel, the paper was submitted to *Epilepsia* for review. A revision task force, composed of equal parts original and new members, was appointed by the ILAE to revise the proposal based on the comments. The final version was approved by the ILAE's Executive Committee.

## 2 MATERIALS AND METHODS

### 2.1 Working group

In January 2023, the Executive Committee appointed a working group comprising 37 experts, with a balanced representation of 19 women and 18 men. The group intentionally mirrored the diverse composition of the ILAE, incorporating members from all ILAE regions: North America (*n* = 7), Latin America (*n* = 5), Europe (*n* = 11), Eastern Mediterranean (*n* = 2), Asia Oceania (*n* = 9), and Africa (*n* = 3). Within the group, 23 experts specialized in adult epileptology, whereas 13 primarily focused on pediatric epileptology. Additionally, one member brought expertise as a neurosurgeon. To ensure continuity, four members were selected from the task force involved in developing the 2017 version.

The working group conducted three workshop meetings; two were conducted entirely online in April and May 2023, and one meeting adopted a hybrid approach, combining face‐to‐face and online elements, held in September 2023 in Dublin, Ireland. Communication primarily occurred electronically, utilizing emails and an online work management platform (Monday.com). Video recordings and comprehensive documentation of the entire process were electronically archived and made accessible to all members throughout the duration of the process. The ILAE office provided technical assistance with the process.

### 2.2 Systematic review

We conducted a systematic review^11^ to evaluate the strengths and weaknesses of the 2017 ILAE seizure classification. We searched PubMed and Embase databases for articles addressing the implementation of the 2017 ILAE seizure classification. Eligibility criteria were as follows: (1) research papers investigating applicability and feasibility of the 2017 seizure classification and (2) review and opinion papers. For the first criterion, we included congress abstracts too, if they provided sufficient details for evaluation. For the second criterion, we excluded congress abstracts and reviews by the authors of the 2017 classification.

Data S1 displays the PRISMA (Preferred Reporting Items for Systematic Reviews and Meta‐Analyses) flow diagram depicting the review process^11^. Two authors (S.B. and E.T.) independently reviewed and rated the records, resolving any disagreements through consensus discussions. Subsequently, the working group further reviewed and edited the outcomes. The review encompassed a total of 41 articles, as detailed in Data S2. Among these, 22 research articles evaluated the applicability and feasibility of the 2017 classification: nine studies supported its feasibility, 11 studies found it partially feasible, and two studies deemed it unfeasible. Additionally, 19 articles comprised reviews and opinions: 10 papers expressed negative critiques, six held neutral positions with an optimistic outlook for future implementations, and three presented mixed opinions (supportive and critical).

### 2.3 Strengths and weaknesses

We clustered strengths and weaknesses extracted from the systematic review (Data S2) alongside additional input provided by the working group members.

Overall, the 2017 seizure classification's strengths lie in its operational approach and basic organization of seizure types, divided into four main classes. It offers flexibility for classification at varying levels of complexity, making it more practical for real‐world clinical use. The addition of the Unknown class was perceived as an improvement, enhancing the feasibility and applicability of the classification system.^12^, ^13^, ^14^ There were differing opinions on the introduction of the term “focal to bilateral tonic–clonic seizure.” However, a prospective study demonstrated that this term facilitated more accurate classification of seizures compared to its synonym in the older version (1981) of the classification system.^15^ The inclusion of more descriptors was seen as a strength, particularly for implementation in databases.^16^ A study validated the usefulness of distinguishing focal from generalized epileptic spasms.^17^

A robust debate occurred regarding the suitability of the term *awareness* to classify seizures, rather than using the term *consciousness*. Several papers pointed out the disadvantages of using awareness as a surrogate marker for *consciousness*.^18^, ^19^, ^20^, ^21^ Conversely, the appropriateness of the concept of *consciousness* in epileptology has also been critically discussed, given the challenges of reliably assessing it during a seizure.^22^ *Impaired consciousness* is a commonly used medical term, broadly implemented in clinical neurology.^23^, ^24^, ^25^, ^26^ It is operationally defined by awareness and responsiveness.^26^, ^27^, ^28^ Based on the concepts of George Berkeley (1685–1753),^29^ William James (1842–1910),^30^ and Arthur Schopenhauer (1788–1860),^31^ Pierre Gloor identified important components of consciousness to include the “ability to attend and perceive, to relate perception to one's own fund of personal memories, to remember recent events and to react voluntarily in response to such stimuli.”^22^ There has been much progress in recent years in understanding mechanisms and developing tools for objectively measuring normal consciousness^32^, ^33^ and impaired consciousness in neurological disorders,^24^, ^25^, ^34^, ^35^ including epilepsy.^36^, ^37^, ^38^, ^39^, ^40^, ^41^, ^42^, ^43^ For general neurologists, an epileptic seizure is a differential diagnosis within conditions of transient loss or impairment of consciousness.^44^ For medical students and similarly to lay persons, *consciousness* is simply explained as the ability to respond and to remember.^45^ The debate against using responsiveness as a classification criterion revolves around its dependence on intact motor functions and its difficulty in outpatient settings, although studies indicate that impaired responsiveness is often reported during patient history‐taking.^21^ In epilepsy monitoring units, responsiveness is frequently evaluated over awareness.^20^ Some clinicians have adopted the term *impaired awareness* to denote impaired responsiveness, believing it aligns with the ILAE position paper, despite this interpretation being incorrect.^21^ In children younger than 4–5 years, assessing awareness is often challenging or impossible,^46^ whereas responsiveness can be evaluated using age‐appropriate methods.^38^ A crucial consideration lies also in the translatability of these terms: *awareness* faces challenges in translation across languages such as Spanish, French, Portuguese, and German,^3^ whereas *consciousness* is more translatable and already a universally accepted medical term.

The clinical relevance of categorizing seizures into *motor* versus *nonmotor* and utilizing the first observed phenomenon as a classifier have been questioned. In contexts such as clinical trials or resource‐limited settings, a more practical dichotomy, “with versus without observable manifestations,” has been considered more beneficial.^47^ Notably, nonmotor seizures may exhibit observable manifestations such as aphasia or flushing. The use of the first semiological phenomenon as a classifier has shown limited clinical relevance. It does not influence critical factors such as the selection of antiseizure medication, prognosis, or the localization of seizures for surgical therapy.^2^, ^3^, ^4^, ^5^, ^19^, ^48^, ^49^ A more clinically relevant approach for characterizing the epileptic network, especially in the context of presurgical evaluation and clinical–anatomic correlation, involves describing the seizure evolution, specifically, the chronological sequence of semiological phenomena.^2^, ^3^, ^4^, ^48^, ^50^, ^51^, ^52^, ^53^

The 2017 classification categorized absences as nonmotor seizures, which is misleading. Typical absence seizures often present observable motor phenomena such as discrete automatisms, head retropulsion, and eye blinks, and atypical absences may involve atonic phenomena.^6^ Notably, motor manifestations are characteristic features of specific absence seizure types, such as eyelid myoclonia with absence and myoclonic absences.^54^

Epileptic negative myoclonus is a well‐documented phenomenon^35^ acknowledged in both the earlier^55^ and the revised version of the ILAE semiology glossary.^56^ It is important to note that epileptic negative myoclonus differs from asterixis found in toxic–metabolic encephalopathies.^57^ Although discussed in prior works, epileptic negative myoclonus was not included in the 2017 classification.

Experimental studies in animal models^58^ and humans^59^ demonstrated the focal onset in generalized seizures,^60^, ^61^, ^62^, ^63^ and this has been incorporated in the current ILAE definitions.^1^, ^64^ The term *generalized onset seizure* seems to be in contradiction with this, and it may be misleading in the clinical practice, because focal onset of generalized seizures was well documented in large survey studies^63^ and video‐electroencephalographic (EEG) recordings.^65^, ^66^, ^67^

Epileptic seizures can be classified using various principles, potentially resulting in numerous seizure types, some of which might be redundant and lack clinical relevance. Establishing clear taxonomic rules is essential to precisely define and differentiate classifiers (used to identify seizure types) from descriptors (used to characterize specific features within a seizure type).

### 2.4 Proposed adjustments

Building upon the strengths and weaknesses discussed and clustered in the previous section, the working group formulated proposals for adjustments. These proposals were later modified during the Delphi process and subsequent revision, as detailed below.

1. Taxonomic rules: We distinguish *classifiers*, which reflect biological classes (conceptual justification) and directly impact clinical management (utilitarian justification), from *descriptors*, which represent key seizure characteristics and indirectly aid patient management when combined with other clinical data. Main seizure classes, seizure types, and level of consciousness are classifiers, whereas semiological features are descriptors.
2. Terminology of the main seizure classes: Change “generalized‐onset seizure” to “generalized seizure,” change “focal‐onset seizure” to “focal seizure,” and change “unknown‐onset seizure” to “unknown whether focal or generalized.”
3. Level of consciousness is also a classifier for focal seizures and for seizures of unknown origin. We propose substituting awareness (aware or impaired awareness) with consciousness (preserved or impaired), operationally defined based on both awareness and responsiveness. Awareness is assessed through recall. Responsiveness is tested using both verbal and motor tasks.
4. Descriptors: We propose replacing the motor versus nonmotor subclassification, within focal seizures and within seizures unknown whether focal or generalized, with a distinction between seizures with observable manifestations and those without, in the basic version of the classification. In the expanded version, we propose describing seizure semiology in chronological sequence, depicting the sequence of seizure phenomena.
5. Epileptic negative myoclonus: Include the recognition of epileptic negative myoclonus within the seizure classification.
6. Generalized seizures: Remove “nonmotor” when categorizing absence seizures.
7. Epileptic spasms: Incorporate epileptic spasm as a semiological descriptor for focal seizures and for seizures unknown whether focal or generalized. Retain epileptic spasms as a seizure type for generalized seizures.

### 2.5 Delphi method

We employed a modified Delphi method^68^ to achieve consensus regarding the proposed adjustments and the update of the seizure classification. For a proposal to pass, it required at least a two‐thirds majority vote from the group. Acting as moderators, two authors (S.B. and E.T.) facilitated the process. They gathered and summarized the votes, incorporating comments, and returned them for the subsequent round, refraining from voting themselves. Throughout the process, 35 members of the working group participated in voting. Individual responses were anonymized to other participants, but after each round, they received a summary of results, along with incoming comments and suggestions.

Consensus was achieved after seven rounds. The first three Delphi rounds focused on addressing the proposals, whereas the subsequent four rounds were dedicated to the entire updated classification system. All implemented proposals garnered more than two thirds of the votes, and the final version received unanimous approval from all members of the working group.

### 2.6 Public comments and revision

The proposed position paper was reviewed by the ILAE Executive Committee for approval of its concept and content and was posted on the ILAE website for public comments from August 12 to October 16, 2024. A total of 44 comments were received from chapter representatives and individual members, in addition to the anonymous peer reviews in *Epilepsia*.

The Executive Committee appointed a revision task force to review and incorporate these comments as appropriate. Chaired by Elaine Wirrell, the revision task force included seven new members appointed by the Executive Committee and seven members from the original working group (see Data S3). The task force categorized the comments by topic and distinguished between supportive/approving and critical/disapproving comments (see Data S4).

Overall, the feedback was positive, with 25 supportive comments, two peer reviews, and five critical comments (see Data S5). The main criticisms were that the proposed changes were introduced too soon and too quickly and represented too much change. The revision task force and the Executive Committee considered the 8‐year interval appropriate for updates based on experience with implementing the 2017 classification, which had not been tested in real‐world practice beforehand. This timeframe aligns with practices in other medical societies and previous epilepsy classifications (e.g., the 1985 classification, revised in 1989). Delaying necessary updates would likely make future implementation more challenging. Community feedback, as evidenced by the systematic literature review, highlighted the need for changes. These updates followed the robust procedure recently adopted for ILAE position papers, incorporating published evidence and real‐world experience. The goal of the revision task force was to ensure the changes were accurate. Because the framework and main terms remain unchanged, the updated seizure classification aligns with the 2017 classification's overarching concept.

Another frequently debated aspect was the return to using consciousness instead of awareness. Most comments supported this change, emphasizing that consciousness is a widely accepted and translatable medical term, operationally defined through the assessment of responsiveness and awareness (recall). Responsiveness, often part of patient history, is assessable even in young children, where awareness may not be applicable.^38^ Since the 2017 classification, describing a nonresponsive patient as having “impaired awareness” became widespread, but is inaccurate. Moreover, using an alternative (“surrogate”) term for consciousness distances epilepsy classification from broader medical standards; an epileptic seizure is a key element in the differential diagnosis of transient impairment of consciousness. Concerns were raised that impairment of consciousness might be misunderstood by patients and caregivers as total loss of consciousness. However, when taking a history, health professionals should ask about responsiveness and recall (as a marker of awareness) during the seizure, then draw conclusions about consciousness themselves, rather than relying on patients or caregivers to label it as impaired or preserved. The medical term *consciousness* can then be explained to patients and caregivers as the ability to respond to verbal and motor tasks and recall during the seizure.

To aid in the correct classification of epileptic spasms, a decision flowchart figure and a detailed explanation were added to the revised paper. All changes made by the revision task force to the working group's original proposal are summarized in Data S4. The revised position paper was submitted to the ILAE Executive Committee for final approval.

## 3 RESULTS

**FIGURE 1:**
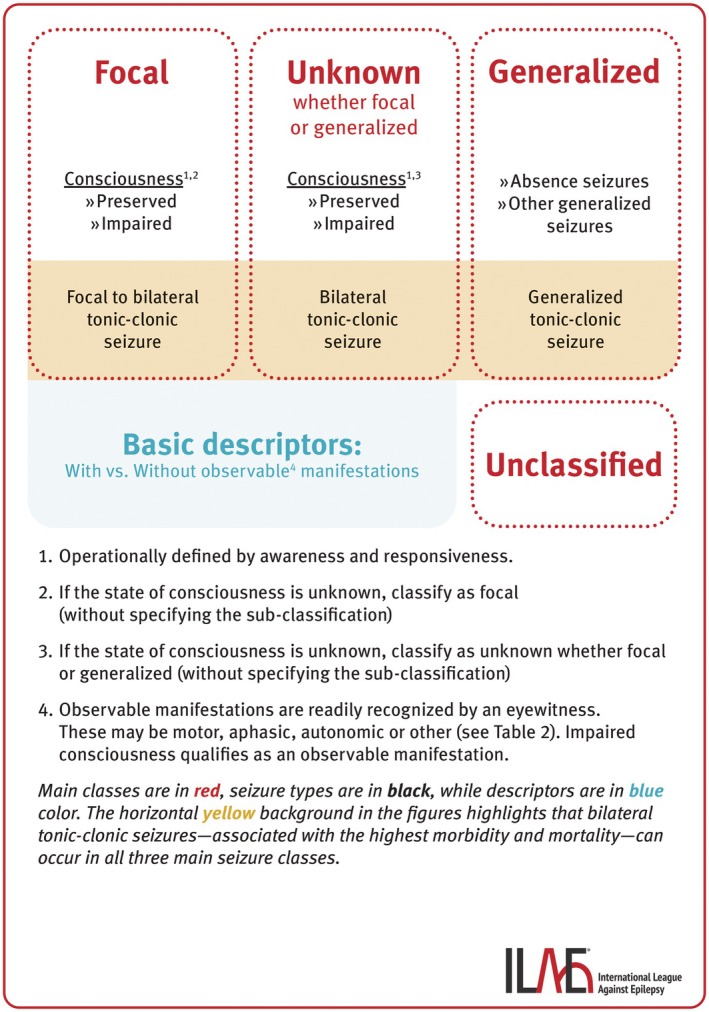
Basic version of the updated seizure classification.

**FIGURE 2:**
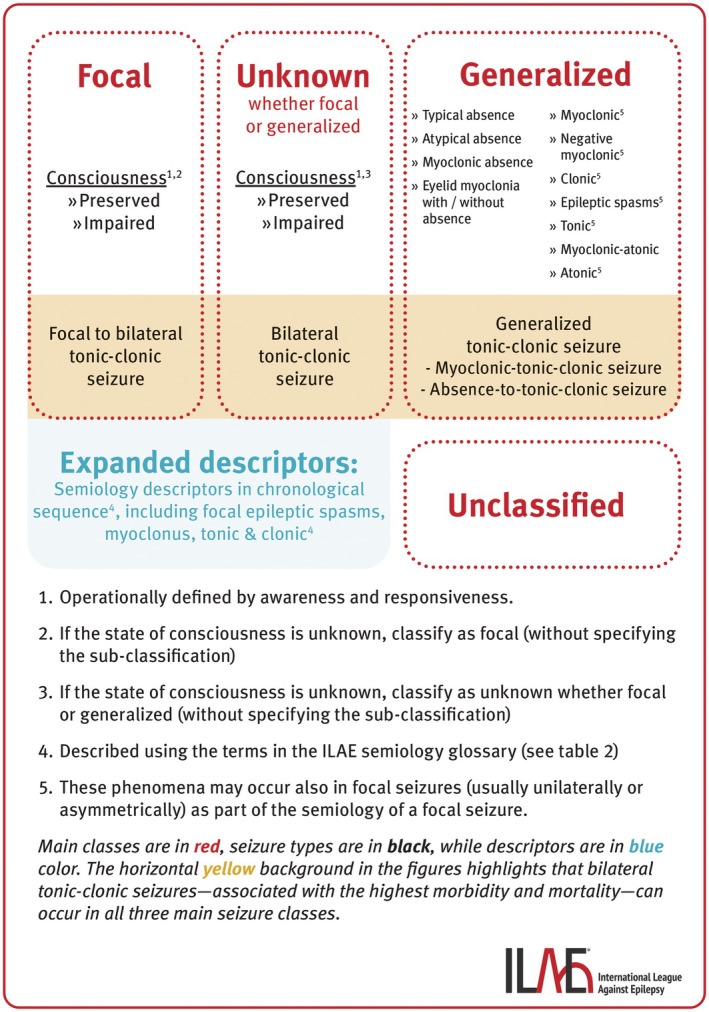
Expanded version of the updated seizure classification.

The fundamental framework for classifying epileptic seizures is maintained.^1^, ^7^ The main seizure classes include Focal, Generalized, Unknown (whether focal or generalized), and Unclassified. Figures 1 and 2 illustrate the basic and expanded seizure classifications, and Table 1 presents the taxonomic hierarchy of seizure classification. *Classifiers* define the seizure types, considered as biological classes with direct influence on patient management by guiding syndrome diagnosis, therapeutic decisions, and prognosis. *Descriptors*, on the other hand, are important clinical characteristics of the seizures that, along with other clinical data and modalities, indirectly contribute to shaping patient management. Descriptors are essential for clinical decisions and, in specific contexts, may significantly influence therapy (e.g., epileptic spasm or myoclonus in the context of a focal seizure).

**TABLE 1:** Taxonomic hierarchy of epileptic seizure classification.

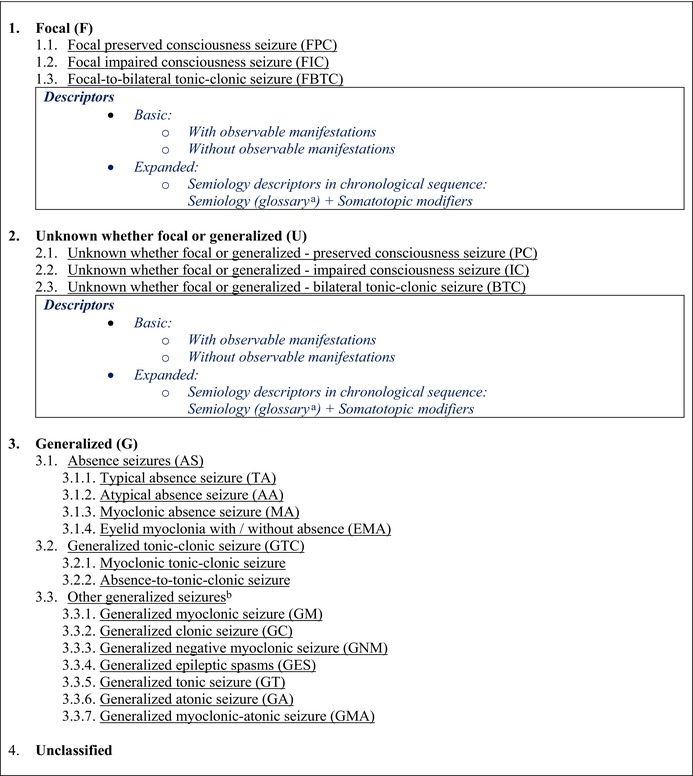

*Note:* Classifiers are shown in black, whereas descriptors are in blue. Main classes are indicated in bold font; seizure types are underlined.

^a^ See Table 2 for semiological features.

^b^ This is a grouping term, not a defined concept.

Focal seizures are defined as originating within networks limited to one hemisphere.^1^, ^64^ They may be discretely localized or more widely distributed and may originate in cortical or subcortical structures. For each seizure type, ictal onset is consistent from one seizure to another, with preferential propagation patterns that may involve the contralateral hemisphere. In some cases, however, there is more than one network, and more than one seizure type, but each individual seizure type has a consistent site of onset.^64^ Focal to bilateral tonic–clonic seizures^1^ are focal seizures in which the ictal activity propagates to both hemispheres, while the semiology evolves to impairment and eventually complete loss of consciousness and bilateral tonic muscle activation, followed by a clonic phase with progressive decrease in frequency, due to a gradual increase in the duration of the silent periods interrupting the tonic muscle activity.^56^

Generalized seizures are defined as originating at some point within, and rapidly engaging, bilaterally distributed networks, which can include cortical and subcortical structures, but not the entire cortex.^1^, ^45^ Seizure onset can appear localized, and seizures can be asymmetric.

When there is information available to characterize certain aspects of seizures, but it is insufficient for a clear classification as focal or generalized, they are categorized as Unknown (whether focal or generalized). In cases where there is no available information to characterize the seizure, but the clinician is confident that the event is an epileptic seizure, it is labeled as Unclassified.^1^ Subsequently, as more information becomes available to the clinician, these seizures can be reclassified as either focal or generalized.

Focal seizures and seizures unknown whether focal or generalized are further classified according to the patient's state of consciousness during the seizure: impaired or preserved. If the state of consciousness is undetermined, the seizure is classified under the parent term (focal seizure or seizure of unknown origin). Consciousness is operationally defined by establishing both awareness and responsiveness, relying on information obtained from medical history^21^ or through behavioral testing by medical personnel.^38^ These operational terms are explained to the patients and caregivers as the ability to remember and to respond appropriately and normally during the seizure. Rather than asking patients and caregivers about consciousness, it is advisable to ask specifically about recall of the events (awareness) and degree of responsiveness during the seizure. An inadequate response or a significantly longer response latency compared to the interictal (baseline) state qualifies as impaired responsiveness.^38^, ^56^ Patients and caregivers may need to be reminded that consciousness can still be impaired although the eyes are open and the patient attempts to interact. In real‐world scenarios, information may be available about only one of these characteristics (awareness or responsiveness). If either is impaired in any way, the seizure is classified as impaired consciousness. It is important to exercise caution and consider isolated epileptic amnesia as a potential cause for the lack of recall of ictal experiences, and to rule out ictal paresis or ictal receptive aphasia as potential causes of unresponsiveness, whenever possible. Seizures with impaired consciousness are inherently considered to have observable manifestations.

Descriptors can be employed to provide additional characterization of seizures. In the basic version, a straightforward dichotomy is utilized; seizures are described as either having observable manifestations or not. Observable manifestations are easily identified by eyewitnesses,^47^ are nonvolitional, and can include motor, aphasic, autonomic, or other features (see Table 2). In the expanded version, seizures are described in detail, by listing in chronological order the semiological features (see Table 2) that occur during the seizure.^56^, ^69^ The sequence is indicated by arrows pointing in the direction of seizure evolution (e.g., epigastric aura ➔ right hand automatism ➔ impaired responsiveness + impaired awareness). All items in the table outlining the semiological features (Table 2) are defined and their significance is explained in detail in the ILAE glossary of seizure semiology.^56^ Additionally, video examples are available for each item.^56^ The ictal evolution offers crucial insights, as it can identify specific conditions, such as epilepsy of infancy with migrating focal seizures,^70^ and aid in the localization of the cortical areas generating the seizures.^56^ Please note that colloquial terms derived from semiology, such as hyperkinetic (or hypermotor) seizures, focal spasms, focal myoclonic seizures, focal clonic seizures, and focal tonic seizures, refer to focal seizures as the main seizure type.

**TABLE 2:** Descriptors for focal seizures and for seizures unknown whether focal or generalized.

Somatotopic modifiers	
Side (left, right, bilateral–symmetric, bilateral–asymmetric) + body part	
Semiological features	
1. Elementary motor phenomena^a^	5. Autonomic phenomena^c^
Akinetic Astatic Atonic Clonic Dystonic Epileptic nystagmus Epileptic spasm Eye blinking Eye deviation Gyratory Head orientation Ictal paresis Myoclonic Myoclonic–atonic Epileptic negative myoclonus Tonic (focal tonic, chapeau de gendarme, fencing posture) Tonic–clonic (figure‐of‐four) Versive	Cardiovascular ○ Ictal asystole ○ Ictal bradycardia ○ Ictal tachycardia Cutaneous/thermoregulatory ○ Flushing ○ Piloerection ○ Sweating Epigastric Gastrointestinal ○ Borborygmi ○ Flatulence ○ Hypersalivation ○ Nausea/vomiting ○ Polydipsia ○ Sialorrhea ○ Spitting Pupillary ○ Miosis ○ Mydriasis Respiratory ○ Apnea ○ Choking ○ Hyperventilation ○ Hypoventilation Urinary ○ Incontinence ○ Urinary urge
2. Complex motor phenomena^a^	
Automatisms ○ Gestural automatisms—distal ○ Gestural automatisms—genital ○ Gestural automatisms—proximal ○ Ictal grasping ○ Mimic automatisms (gelastic, dacrystic) ○ Oroalimentary automatisms ○ Verbal automatisms ○ Vocal automatisms Hyperkinetic behavior	
	6. Affective (emotional) phenomena^c^
	Anger Anxiety Ecstatic/bliss Fear Guilt Mirth Mystic Sadness Sexual
3. Sensory phenomena^b^	
Auditory Body‐perception illusion Depersonalization Gustatory Olfactory Somatosensory ○ Painful ○ Nonpainful Vestibular/dizziness Visual	
	7. Indescribable aura^b^
	Postictal phenomena ○ Autonomic signs ○ Blindness (hemianopsia or amaurosis) ○ Confusion ○ Headache ○ Language dysfunction ○ Nose‐wiping ○ Palinacousis ○ Paresis (Todd's paresis) ○ Psychiatric signs ○ Unresponsiveness
4. Cognitive and language phenomena^c^	
Aphasia Confusion/disorientation Dysmnesia ○ Amnesia ○ Déjà vu/déjà vécu/jamais vu/dreamy state/reminiscence Forced thinking Other focal cognitive deficits (e.g., anosognosia, apraxia, neglect)	

*Note*: If phenomena not listed above occur during the seizure, they are added in free text. Awareness and responsiveness define consciousness and hence are classifiers. All items in this table are defined in the International League Against Epilepsy glossary of semiology.

^a^ Observable manifestations.

^b^ Not observable manifestations.

^c^ Possibly observable manifestations.

The descriptors are based on seizure semiology. We acknowledge the importance of other clinically relevant seizure characteristics, such as the context of occurrence (e.g., reflex or sleep‐related) and the anatomical localization of the epileptogenic zone. Although these characteristics are not formally included in the seizure classification, they are valuable in clinical practice and research.

In the basic version of seizure classification, generalized seizures are grouped into absence seizures, generalized tonic–clonic seizures, and other generalized seizures. The latter is a grouping term, not a defined concept. In the figures illustrating seizure classification, tonic–clonic seizures are positioned at the end of each main class: focal to bilateral tonic–clonic seizures, generalized tonic–clonic seizures, and bilateral tonic–clonic seizures of unknown origin (whether focal or generalized). This placement highlights these seizure types, which are associated with the highest morbidity and mortality, and are the major risk factor for sudden unexpected death in epilepsy.^71^, ^72^, ^73^, ^74^ In the expanded seizure classification, all generalized seizure types are listed (Figure 2 and Table 1). Definitions of all generalized seizure types are provided in Data S5.

It is acknowledged that generalized tonic–clonic seizures may be heralded by myoclonic jerks or an absence seizure, a distinction reflected in the subtypes of this seizure.^1^, ^75^, ^76^ If these specific features (myoclonic jerks or absence at onset) are not observed, the seizure is classified under the parent term, generalized tonic–clonic. Generalized negative myoclonus is now recognized as a distinct seizure type, whereas the other generalized seizure types remain consistent with the 2017 classification.^1^ Generalized tonic seizures may be preceded or followed by spasms (colloquially termed “tonic spasms”), a myoclonic jerk (“myoclonic–tonic seizure”), or a hyperkinetic seizure followed by a spasm (“hypermotor–tonic–spasms”). Although evidence suggests that some of these combinations of seizure types may be relevant for syndromic diagnosis (e.g., hypermotor–tonic–spasms in *CDKL5* deficiency disorder), they are not yet formally included in the seizure classification. Further research is needed to establish the clinical significance of these tonic seizure subtypes.

**FIGURE 3:**
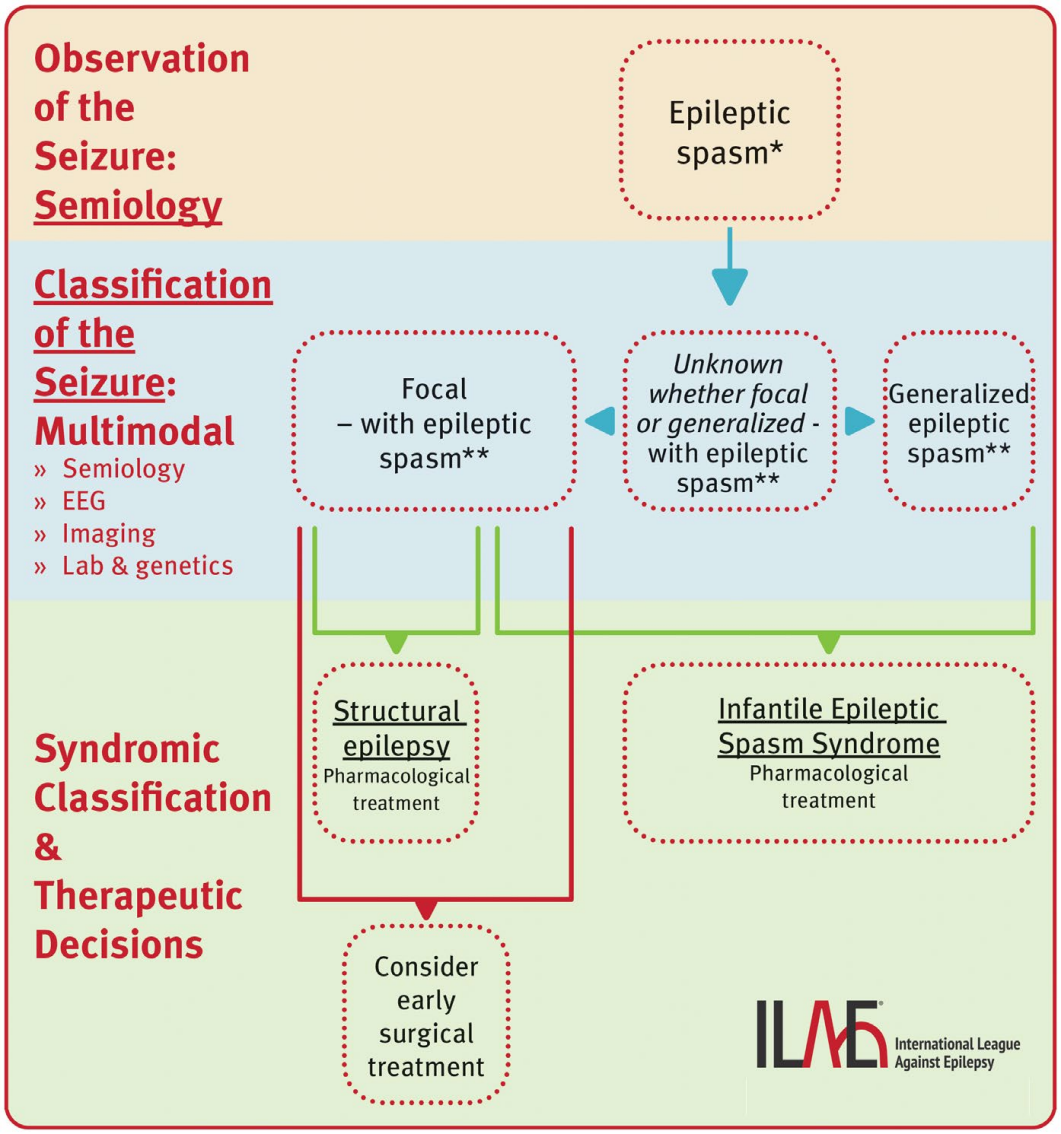
Decision flowchart for classifying epileptic spasms and their relevance to syndromic diagnosis and treatment. EEG, electroencephalography. *semiology; **seizure type.

Epileptic spasms represent an important ictal phenomenon (Table 2), and their early recognition and accurate classification is essential for optimal treatment.^1^, ^70^ Although spasms can be generalized, focal, or unknown whether focal or generalized, the most critical aspect in infants is timely recognition of this unique seizure type and initiation of spasms‐specific therapies, as delay can result in poorer developmental outcomes.^77^ Discerning whether spasms are focal or generalized can be challenging (Figure 3) and require a multimodal approach.^70^ Within the generalized main class, epileptic spasms are a *classifier*, often associated with infantile epileptic spasms syndrome (IESS).^70^ In the focal and unknown classes, epileptic spasms are a *descriptor* and thus described within the seizure semiology (e.g., focal epileptic spasm). In the context of the clinical data (including age at onset), they lead to the syndromic diagnosis of IESS,^70^ and pharmacological therapy specific for this syndrome must be initiated without delay. Furthermore, in cases of focal epileptic spasms (unilateral or asymmetric semiology) or when other findings, such as neuroimaging, suggest a focal origin, early surgical treatment should be considered, particularly if spasms‐specific therapies have failed (Figure 3). Epileptic spasms can also occur in older age groups, outside IESS, which led to the terminology shift from infantile spasms to epileptic spasms.^1^ In these cases, the pharmacological treatment differs from IESS (Figure 3). Besides epileptic spasms, other motor ictal phenomena, including myoclonus, clonus, and tonic muscle contractions can occur in both generalized seizures (defining the seizure type) and in focal seizures, where they typically present unilaterally or asymmetrically as part of the focal seizure semiology (Figure 2).

Epileptic seizures are classified within a taxonomic hierarchy, comprising main classes and seizure types (Table 1). We found it important to explicitly outline the specific list of seizure types, following the principles illustrated in the figures and detailed in this paper. The table aims to provide clear guidance for electronic databases. The seizure classification includes four main classes and 21 seizure types, a significant simplification compared to the 2017 edition, which included 63 seizure types.^5^, ^78^ The updated classification retains the flexibility of the 2017 edition. The classification of an individual seizure can halt at any level on the hierarchical tree, and seizures initially labeled as unknown or unclassified can be later reclassified, as new information about the seizure becomes available.

Although the updated seizure classification places significant emphasis on seizure semiology and can be applied in resource‐limited settings, similar to the 2017 edition, it remains interpretative. This allows for the incorporation of supplementary data to identify the seizure types.^1^ In alignment with clinical practice, it is recommended to classify seizures by considering all available information, encompassing semiology and supportive data such as EEG, neuroimaging, laboratory results, and genetics.

In the following section, we illustrate the implementation of the updated seizure classification, utilizing examples from the previous edition and from the articles that criticized it.^4^, ^79^, ^80^

A young woman awakens to find her 20‐year‐old boyfriend having a seizure in bed. The onset is not witnessed, but she is able to describe bilateral stiffening followed by bilateral “shaking.” EEG and magnetic resonance imaging (MRI) findings are normal. This seizure is classified as bilateral tonic–clonic seizure—unknown whether focal or generalized (BTC; 2.3).

In an alternate scenario of the previous case, the EEG shows a clear right parietal slow‐wave focus. The MRI shows a right parietal region of cortical dysplasia. In this circumstance, the seizure is classified as focal to bilateral tonic–clonic seizure (FBTC; 1.3).

A 25‐year‐old woman describes seizures beginning with 30 s of an intense feeling that “familiar music is playing.” She can hear other people talking but afterward realizes that she could not determine what they were saying. Eyewitnesses report that the patient does not respond to external stimuli during the seizure, neither verbal nor tactile (touching the patient). After an episode, she is mildly confused and has to “reorient herself.” The seizure is classified as focal impaired consciousness seizure (FIC; 1.2) with the following evolution: auditory aura ➔ receptive aphasia ➔ impaired responsiveness ➔ postictal confusion.

A 22‐year‐old man has seizures during which he remains fully aware, with the “hair on my arms standing on edge” and a feeling of being flushed. These are classified as focal preserved consciousness seizure (FPC; 1.1) with observable manifestations: piloerection + flushing.

A 13‐year‐old with juvenile myoclonic epilepsy has seizures beginning with a few jerks, followed by stiffening of all limbs and then rhythmic jerking of all limbs. These are classified as generalized myoclonic‐tonic–clonic seizures (GTC; 3.2.1).

A 3‐month‐old boy has clusters of short seizures with flexion in the neck and hips, and abduction in the shoulders of short duration (up to 2 s). The patient has 3–15 clusters per day. The child was encephalopathic, without developmental progression. Seizures were resistant to multiple antiseizure medications, including adrenocorticotropic hormone. Repeated MRI was unrevealing. Video‐EEG showed epileptic spasms associated with a generalized suppression on EEG. The seizure is classified as generalized epileptic spasm (GES; 3.3.4).

A 14‐month‐old girl has sudden extension of both arms and flexion of the trunk for approximately 2 s. These seizures repeat in clusters. EEG shows hypsarrhythmia with bilateral spikes, most prominent over the left parietal region. MRI shows left parietal cortical dysplasia. Because of the ancillary information, the seizure is classified as focal seizure (F; 1) with epileptic spasms (brief version: focal epileptic spasms).

During long‐term video‐EEG monitoring, a 28‐year‐old female patient experiences an ascending sensation from the stomach and then starts chewing and manipulating nearby objects using the right hand. The patient can recall what happens during these episodes and is able to respond. The seizure is classified as focal preserved consciousness seizure (FPC; 1.1) with observable manifestations as follows: epigastric aura ➔ oroalimentary automatisms + gestural automatisms with the right hand + preserved awareness and responsiveness.

An 8‐year‐old boy reports episodes starting with seeing colored dots and stripes on the left side. The patient cannot recall what happened after that, but eyewitnesses report that the patient does not respond to verbal and tactile stimuli, turns the head to the left, becomes stiff, and then has jerks in all limbs. The seizure is classified as focal‐to‐bilateral tonic–clonic seizure (FBTC; 1.3) with observable manifestations as follows: elementary visual aura on the left side ➔ versive to left + loss of awareness and responsiveness ➔ bilateral tonic–clonic.

A 33‐year‐old, right‐handed man experienced febrile seizures in infancy. Habitual, unprovoked seizures started at the age of 15 years and were accompanied by a feeling of abdominal discomfort followed by loss of awareness. His wife reported that approximately once per month he displays episodes of lip smacking, fumbling hand movements, and occasional right‐hand posturing. The seizure is classified as focal impaired consciousness seizure (FIC; 1.2) with the following: epigastric aura ➔ impaired awareness ➔ oroalimentary automatisms + gestural automatisms + dystonic posturing in the right hand.

## 4 DISCUSSION

The revised seizure classification adheres to the same framework as the 2017 version, maintaining the four main classes. In addition to the archetypical classes of Focal and Generalized seizures, two more main classes are included for practical reasons: Unknown (for cases where the distinction cannot be made) and Unclassified (a temporary class, when no further information is available about the seizure). The impetus for the update arose from the collective experiences after applying the 2017 seizure classification and an iterative discourse of the international epilepsy community. The 2017 version was anticipated to require adjustments based on the insights gained during its implementation in clinical practice.

The working group employed a robust yet conservative methodology, based on a systematic analysis of the strengths and weaknesses of the 2017 version. Proposals for updates were only considered if they addressed a problem documented in the literature. Approval of any proposal required more than two thirds of the votes in the Delphi process. The large working group represented the diversity of the ILAE, encompassing broad representation from all regions and various subspecialties, allowing for a broad discussion on the ontological relativity of the terms used in the 2017 classification and widely varying conceptual schemes in different languages. The proposal was posted for public comment, and a newly appointed task force revised the document based on relevant community feedback. Much like the 2017 edition, the primary objective was to establish a common language and framework for clinical practice. With a focus on flexibility, the classification aims to accommodate diverse settings, ranging from resource‐limited areas to highly specialized centers. Simultaneously, it strives to offer a well‐defined and clear structure, suitable for implementation in research databases and clinical trials.

Special emphasis was placed on ensuring the coherence and internal consistency of the classification. Following traditional principles employed in scientific classification systems, we established clear taxonomic rules derived from clinical and conceptual reasoning. Features directly impacting patient management were designated as *classifiers*, whereas other seizure characteristics served as *descriptors*. These were organized within the taxonomic hierarchy, resulting in four main classes and a total of 21 seizure types. The descriptors were structured into two layers: in the basic version, based on the dichotomy of observable ictal manifestations or the lack thereof, and in the expanded version, organized according to the chronological sequence of seizure semiology. The numbering in the taxonomic hierarchy list is designed to ensure consistency across databases and languages, mitigating any potential ambiguity.

To keep the classification system as simple as possible, we refrained from introducing neologisms. Instead, we utilized established medical terminology commonly found in literature and ensured translatability into languages beyond English. The classification has been translated into 14 languages (Data S6), providing broad, global coverage: Arabic, Chinese, Danish, French, German, Hungarian, Italian, Japanese, Korean, Portuguese, Romanian, Russian, Spanish, and Ukrainian. We aimed to create a system that is easily communicable to both patients and caregivers. A PowerPoint file with the updated seizure classification is available online (Data S7)

The changes included in the updated seizure classification are summarized in Table 3. The term *onset* has been omitted from the names of the main seizure classes, as there is compelling evidence suggesting focal onset in generalized seizures as well.^58^, ^59^, ^60^, ^61^, ^65^, ^66^, ^67^, ^81^ The names of these classes now align with their definitions in the ILAE position papers.^1^, ^64^

**TABLE 3:** Key changes in seizure classification from 2017 to 2025.

“Onset” is removed from the names of the main seizure classes. A distinction is made between classifiers and descriptors, based on taxonomic rule. Consciousness is used as a classifier instead of awareness, with consciousness operationally defined by awareness and responsiveness. The motor vs. nonmotor dichotomy is replaced by observable vs. nonobservable manifestations. The chronological sequence of seizure semiology is used to describe seizures, rather than relying solely on the first sign. Epileptic negative myoclonus is recognized as a seizure type.

Both awareness and responsiveness are used to operationally characterize consciousness, which is now the classifier. This aligns with Gloor's recommendation to “observe accurately and interact with the patient during an attack.”^22^ The motor versus nonmotor dichotomy was extended to observable versus nonobservable manifestations, which is deemed advantageous for clinical trials. This is now considered a descriptor in the basic version of the seizure classification. In the expanded version, the entire chronological sequence of seizure semiology is utilized for describing the seizure, rather than just the initial sign. This approach was considered more suitable for advanced settings, such as long‐term video‐EEG monitoring and presurgical evaluation.

The term *nonmotor* has been removed from absence seizures due to the presence of motor phenomena that may be observed during them, some of which are characteristic of certain types of absence seizures (e.g., myoclonic absence, eyelid myoclonia with absence). Negative myoclonus is now recognized as a seizure type. Within generalized seizures, epileptic spasm is considered a seizure type, whereas within focal seizures and seizures of unknown origin, epileptic spasm is described as part of the seizure semiology (e.g., focal epileptic spasm). Similarly, motor phenomena defining generalized seizure types (myoclonic, tonic, atonic) may also be part of the semiology of a focal seizure.

The updated classification maintains continuity with the 2017 edition, so that seizures already classified with the previous version can easily be converted. For example, impaired awareness translates to impaired consciousness, and a motor seizure is an observable manifestation.

These adjustments of the updated seizure classification were based on experience with the application of the 2017 version. They are relatively minor modifications that preserve the fundamental framework of seizure classification. The aim is to enhance broad clinical applicability across diverse settings and consequently aid useability of the classification.

## CONFLICT OF INTEREST STATEMENT

B.J. serves as associate editor of the journal *Neurology*. She has received research support from NIH, CDC, and Neuropace. D.C. has received educational grants from UCB, Astra Zeneca, and Desitin and is a member of the advisory board of Astra Zeneca and UCB. D.C. is a chair of the Education and Training Committee and of the Guidelines Committee of the European Pediatric Neurology Society. D.C. has no conflicts of interest related to this article. E.T. has received personal honoraria for lectures and educational activities from EVER Pharma, Marinus, Arvelle, Angelini, Alexion, Argenx, Medtronic, Biocodex, Bial‐Portela & Ca, NewBridge, GL Pharma, GlaxoSmithKline, Boehringer Ingelheim, LivaNova, Eisai, Epilog, UCB, Biogen, Sanofi, Jazz Pharmaceuticals, and Actavis; his institution has received research grants from Biogen, UCB Pharma, Eisai, Red Bull, Merck, Bayer, the European Union, FWF Osterreichischer Fond zur Wissenschaftsforderung Bundesministerium für Wissenschaft und Forschung, and Jubiläumsfond der Österreichischen Nationalbank. E.W. serves as a data and safety monitoring board member for Neurocrine, Acadia, GRIN Therapeutics, and Encoded. F.C. has received speaker honoraria or consultancy fees from UCB Pharma, Eurofarma, Libbs, Torrent, Adium, Abbott, Prati Donaduzzi, Takeda, and Biocodex. He has also received institutional grants from the Sao Paulo Research Foundation and Conselho Nacional de Desenvolvimento Científico e Tecnológico. He is the editor‐in‐chief of *Epilepsia*. J.F. receives salary support from the Epilepsy Foundation and from the Epilepsy Study Consortium for consulting work and/or attending scientific advisory boards for Acadia Pharmaceuticals, Acuta Capital Partners, Agrithera, Alterity Therapeutics Limited, Angelini Pharma, Autifony Therapeutics Limited, Axonis Therapeutics, Baergic Bio, Beacon Biosignals, Biogen, Biohaven Pharmaceuticals, Bloom Science, Bright Minds Biosciences, Camp4 Therapeutics Corporation, Cerebral Therapeutics, Cerecin, Cerevel, Cognizance Biomarkers, Cowen and Company, Crossject, Eisai, Encoded Therapeutics, Engrail, Epalex, Epitel, Equilibre BioPharmaceuticals, Genentech, GRIN Therapeutics, IQVIA RDS, iQure Pharma, Janssen Pharmaceutica, Jazz Pharmaceuticals, Korro Bio, Leal Therapeutics, Lipocine, LivaNova, Longboard Pharmaceuticals, Marinus, Modulight.bio, Neumirna Therapeutics, Neurocrine, Neuronetics, NeuroPace, NeuroPro Therapeutics, Neuroventis, Ono Pharmaceutical Co., Otsuka Pharmaceutical Development, Ovid Therapeutics, Paladin Labs, Praxis, PureTech, Rapport Therapeutics, Receptor Holdings, Sage Therapeutics, SK Life Sciences, Stoke, Supernus, Takeda, Third Rock Ventures, UCB, Ventus Therapeutics, Vida Ventures Management, and Xenon. J.F. has also received research support from the Epilepsy Study Consortium (funded by Eisai and UCB), Epilepsy Study Consortium/Epilepsy Foundation (funded by UCB), GW/FACES/One8Foundation, and NINDS. She is on the editorial board of *Lancet Neurology* and *Neurology Today*. She is chief medical/innovation officer for the Epilepsy Foundation. She is the president and on the board of directors for the Epilepsy Study Consortium. She has received travel/meal reimbursement related to research, advisory meetings, or presentation of results at scientific meetings from the Epilepsy Study Consortium, the Epilepsy Foundation, Angelini Pharma, Biohaven Pharmaceuticals, Cerebral Therapeutics, Cowen and Company, Longboard, Neurelis, Neurocrine, NeuroPace, Praxis, Rapport, SK Life Science, Stoke, Takeda, and Xenon. J.H.C. has acted as an investigator for studies with GW Pharma/Jazz Pharmaceuticals, Zogenix/UCB, Vitaflo, Stoke Therapeutics, Ultragenyx, and Marinius. She has been a speaker and on advisory boards for Jazz Pharmaceuticals, UCB, Biocodex, and Nutricia; all remuneration has been paid to her department. She holds an endowed chair at UCL Great Ormond Street Institute of Child Health; she holds grants from National Institute of Health Research (NIHR), EPSRC, GOSH Charity, LifeARC, and the NIHR Biomedical Research Centre at Great Ormond Street Hospital. She is president of the ILAE 2021–2025. J.W. is on the national (South African) advisory board for Novartis and Sanofi and is an associate editor of *Epilepsia* (honorarium for work covered). M.S. served as editor‐in‐chief of *Epilepsia*. He has received compensation for speaking in continuing medical education (CME) programs from Medscape. He has consulted for Medtronic, Neurelis, and Johnson & Johnson. He has received research support from Medtronic, SK Life Science, Takeda, Xenon, Cerevel, UCB Pharma, Janssen, Equilibre, Epiwatch, Byteflies, and Biohaven. He has received royalties from Oxford University Press and Cambridge University Press. N.S. has served on scientific advisory boards for GW Pharma, BioMarin, Arvelle, Marinus, and Takeda; has received speaker honoraria from Eisai, BioMarin, LivaNova, and Sanofi; and has served as an investigator for Zogenix, Marinus, BioMarin, UCB, and Roche. He has been supported by #NEXTGENERATIONEU and funded by the Ministry of University and Research, National Recovery and Resilience Plan, project MNESYS (PE0000006): A Multiscale Integrated Approach to the Study of the Nervous System in Health and Disease (DN. 1553 11.10.2022). He has also been supported by the Italian Ministry of Health with Current Research Funds. S.A. is deputy editor for *Epilepsia*; has served as a consultant or received honoraria for lectures from Angelini Pharma, Biocodex, Eisai, Encoded, Jazz Pharmaceutics, GRIN Therapeutics, Neuraxpharm, Nutricia, Orion, Proveca, Stoke, Takeda, UCB Pharma, and Xenon; and has been an investigator for clinical trials for Eisai, Marinus, UCB Pharma, Proveca, and Takeda. S.B. serves as editor‐in‐chief of *Epileptic Disorders*. He has received compensation for speaking in CME programs from Lundbeck, Eisai, UCB and GSK. He has received research support from Independent Research Fund Denmark, Innovation Fund Denmark, European Union: Eurostars Program/EUREKA, European Union: Horizon Europe Framework Program, and Danish Agency for Higher Education and Science: International Network Program. S.W. has received educational grants on behalf of his institution from UCB Pharma, Jazz Pharmaceuticals, and Paladin Labs and has served on the advisory board of Paladin Labs. The remaining authors report no conflicts of interest directly related to this paper. We confirm that we have read the Journal's position on issues involved in ethical publication and affirm that this report is consistent with those guidelines.

## Supporting information


Data S1.

Data S2.

Data S3.

Data S4.

Data S5.

Data S6.

Data S7.

## Data Availability

The data that support the findings of this study are available on request from the corresponding author.
