# Supplementary material for: Updated classification of epileptic seizures: Position paper of the International League Against Epilepsy
Source: Epilepsia. 2025 Apr 23;66(6):1804–23. doi: 10.1111/epi.18338 (PMC12169392; doi:10.1111/epi.18338)
Supplement: Supplementary file 1 — Data S1. [file EPI-66-1804-s005.pdf]

PRISMA flow diagram for new systematic reviews

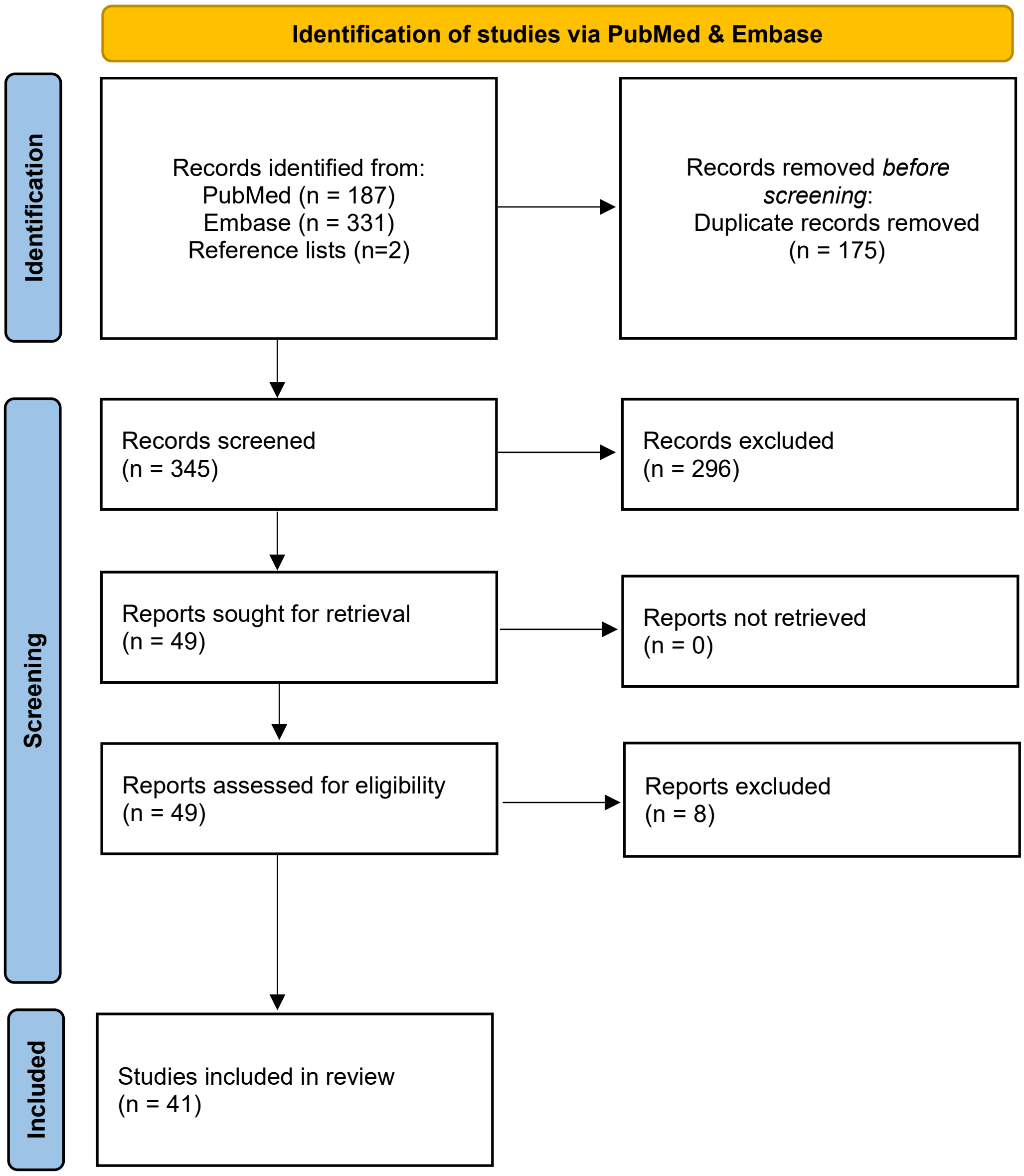

From: Page MJ, McKenzie JE, Bossuyt PM, Boutron I, Hoffmann TC, Mulrow CD, et al. The PRISMA 2020 statement: an updated guideline for reporting systematic reviews. BMJ 2021;372:n71. doi: 10.1136/bmj.n71

http://www.prisma-statement.org/
