# Supplementary material for: Updated classification of epileptic seizures: Position paper of the International League Against Epilepsy
Source: Epilepsia. 2025 Apr 23;66(6):1804–23. doi: 10.1111/epi.18338 (PMC12169392; doi:10.1111/epi.18338)
Supplement: Supplementary file 3 — Data S3. [file EPI-66-1804-s004.docx]

**The Revision Taskforce**
