# Supplementary material for: Updated classification of epileptic seizures: Position paper of the International League Against Epilepsy
Source: Epilepsia. 2025 Apr 23;66(6):1804–23. doi: 10.1111/epi.18338 (PMC12169392; doi:10.1111/epi.18338)
Supplement: Supplementary file 4 — Data S4. [file EPI-66-1804-s001.docx]

**Details on the Revision of the Proposal Submitted for Public Comments**

| *A. List of Key Changes Made by the Revision Taskforce After the Public Hearing*  *B. Changes tracked in the version submitted for public comments*  *C. Changes tracked in the definition of the generalized seizures*  *D. The list of public comments, grouped according to the topic they addressed* |
| --- |

1. **List of Key Changes Made by the Revision Taskforce After the Public Hearing**

- To align with SNOMED ontology, 'unknown state of consciousness' was removed as a seizure type from focal seizures and seizures of unknown origin. Seizures with undetermined state of consciousness are now classified under the parent term (focal seizure or seizure of unknown origin).
- “Tonic-clonic seizure unknown whether focal or generalized” was renamed “Bilateral tonic-clonic seizure”.
- The grouping terms under Generalized seizures, in the basic version, were renamed: absences, generalized tonic-clonic seizures and other generalized seizures.
- In the figures, “unknown whether focal or generalized” is positioned between focal and generalized seizures, while basic descriptors are placed under their applicable seizure types. The horizontal yellow background in the figures highlights that bilateral tonic-clonic seizures—associated with the highest morbidity and mortality—can occur in all three main seizure classes.
- Specification added: “Descriptors are important for clinical decisions and, in specific contexts, may influence therapy (e.g., spasm in the context of a focal seizure).”
- To aid in the correct classification of epileptic spasms, Figure 3 and a detailed explanation were added to the paper.
- Definitions of generalized seizures were updated (see section C).

1. **Changes tracked in the version submitted for public comments**

Updated classification of epileptic seizures: Position paper of the International League Against Epilepsy

Sándor Beniczky^1^*, Eugen Trinka^2^*, Elaine Wirrell^3^, Fatema Abdulla^4^, Raidah Al Baradie^5^, Mario Alonso Vanegas^6^, Stéphane Auvin^7^, Mamta Bhushan Singh^8^, Hal Blumenfeld^9^, Alicia Bogacz Fressola^10^, Roberto Caraballo^11^, Mar Carreno^12^, Fernando Cendes^13^, Augustina Charway^14^, Mark Cook^15^, Dana Craiu^16^, Birinus Ezeala-Adikaibe^17^, Birgit Frauscher^18^, Jacqueline French^19^, MV Gule^20^, Norimichi Higurashi^21^, Akio Ikeda^22^, Floor E. Jansen^23^, Barbara Jobst^24^, Philippe Kahane^25^, Nirmeen Kishk^26^, Ching Soong Khoo^27^, Kollencheri Puthenveettil Vinayan ^28^, Lieven Lagae^29^, Kheng-Seang Lim^30^, Angelica Lizcano^31^, Aileen McGonigal^32^, Katerina Tanya Perez-Gosiengfiao^33^, Philippe Ryvlin^34^, Nicola Specchio^35^, Michael Sperling^36^, Hermann Stefan^37^, William Tatum^38^, Manjari Tripathi^39^, Elza Márcia Yacubian^40^, Samuel Wiebe^41^, Jo Wilmshurst^42^, Dong Zhou^43^, J Helen Cross^44^

^*^ *Contributed equally*

1. Department of Neurology, Aarhus University Hospital, member of European Reference Network EpiCARE; Department of Clinical Medicine, Aarhus University; Department of Clinical Neurophysiology, Danish Epilepsy Centre, Dianalund, Denmark. ORCID 0000-0002-6035-6581
2. Department of Neurology, member of European Reference Network EpiCARE, Center for Cognitive Neuroscience, Christian Doppler University Hospital, Paracelsus Medical University, Salzburg, Austria; Neuroscience Institute, Center for Cognitive Neuroscience, Christian Doppler University Hospital, Paracelsus Medical University, Salzburg, Austria; Institute of Public Health, Medical Decision Making and Health Technology Assessment, University for Health Sciences, Medical Informatics, and Technology, Hall in Tyrol, Austria. ORCID 0000-0002-5950-269
3. Divisions of Child and Adolescent Neurology and Epilepsy, Department of Neurology, Mayo Clinic, USA
4. Department of Clinical Neurosciences, Salmaniya Medical Complex, Government Hospitals, Kingdom of Bahrain
5. King Fahd Specialist Hospital Dammam, Saudi Arabia
6. International Epilepsy Center HMG Coyoacán, Mexico City, Mexico
7. APHP, Robert Debré University Hospital, Pediatric Neurology Department, CRMR epilepsies rares, EpiCare member, Paris, France- Université Paris Cité, INSERM NeuroDiderot, Paris, France - Institut Universitaire de France, (IUF), Paris, France
8. Department of Neurology, All India Institute of Medical Sciences, New Delhi, India
9. Departments of Neurology, Neuroscience and Neurosurgery, Yale University School of Medicine, New Haven, CT, USA
10. Neurological Institute, Universidad de la República (UDELAR) Montevideo, Uruguay
11. Hospital de Pediatría Juan P Garrahan, Buenos Aires, Argentina
12. Clinical Institute of Neurosciences, University Hospital Clinic of Barcelona, Barcelona (HCP), Spain; Epilepsy Unit, Neurology Department, University Hospital Clinic of Barcelona (HCP), Barcelona, Spain
13. Department of Neurology, School of Medical Sciences, University of Campinas (UNICAMP), Campinas, São Paulo, Brazil, and The Brazilian Institute of Neuroscience and Neurotechnology (BRAINN), Campinas, São Paulo, Brazil
14. 37 Military Hospital, Accra, Ghana
15. Faculties of Engineering and Information Technology, and Medicine, Dentistry, and Health Sciences, University of Melbourne, Australia
16. “Carol Davila” University of Medicine Bucharest, Neuroscience Department, Pediatric Neurology Discipline; Center of Expertise of Rare Pediatric Neurological Disorders Al. Obregia Clinical Hospital, Bucharest, Romania, member of European Reference Network EpiCARE
17. Department of Medicine, University of Nigeria Teaching Hospital, Ituku/Ozalla, Enugu. Nigeria. Neurology Unit Memfys Hospital Enugu, Nigeria.
18. Department of Neurology, Duke University School of Medicine, Durham, NC, USA and Department of Biomedical Engineering, Duke Pratt School of Engineering, Durham, NC, USA
19. Department of Neurology, New York University, New York City, NY, USA
20. University of Cape Town, Neuroscience Institute and Groote Schuur Hospital, Department of Medicine, Division of Neurology, South Africa; The Centre for Global Epilepsy, University of Oxford, Oxford, UK
21. Musashi-Kosugi Pediatrics & Epilepsy Clinic and Department of Pediatrics, Jikei University School of Medicine, Japan
22. Department of Epilepsy, Movement Disorders and Physiology, Kyoto University Graduate School of Medicine Shogoin, Sakyo-ku, Kyoto, 606-8507, Japan
23. Department of Child Neurology, Brain Center, University Medical Center Utrecht, the Netherlands. Member of European Reference Network EpiCare.
24. Department of Neurology, Dartmouth-Hitchcock Health, Geisel School of Medicine at Dartmouth, Lebanon, NH, USA
25. Neurology Department, CHU Grenoble Alpes, Univ. Grenoble Alpes, Inserm, U1216, Grenoble Institut Neurosciences, 38000 Grenoble, France
26. Department of Neurology, School of Medicine, Kasralainy Hospital ,Cairo University, Cairo, Egypt
27. Faculty of Medicine, Universiti Kebangsaan Malaysia, Kuala Lumpur; Neurology Unit, Department of Medicine, Hospital Canselor Tuanku Muhriz, Kuala Lumpur, Malaysia and Centre for Global Epilepsy, Wolfson College, University of Oxford, Oxford, United Kingdom
28. Department of Pediatric Neurology and Amrita Advanced Center for Epilepsy , Amrita Institute of Medical Sciences, Cochin, Kerala, India
29. Pediatric Neurology , University of Leuven Belgium, member of European Reference Network EpiCARE
30. Division of Neurology, Department of Medicine, Faculty of Medicine, University Malaya, Kuala Lumpur, Malaysia
31. Department of Clinical Neurophysiology and Epilepsy Clinic, Neurocentro and Coneuro, Colombia; Laboratory of Neuroimmunology, Medcare, Colombia.
32. Centre for Neurosciences, Mater Hospital, Brisbane and The University of Queensland, Australia. ORCID 0000-0001-6775-5318
33. Department of Neurosciences, University of the Philippines-Philippine General Hospital; Department of Neurosciences, Makati Medical Center, Philippines; Institute for Neurological Sciences, St Lukes Medical Center Global City, Philippines
34. Department of Clinical Neurosciences, Centre Hospitalier Universitaire Vaudois and Université de Lausanne, Lausanne, Switzerland, member of European Reference Network EpiCARE
35. Neurology, Epilepsy and Movement Disorders Unit, Bambino Gesu’ Children’ Hospital, IRCCS, member of European Reference Network EpiCARE, Rome, Italy
36. Jefferson Comprehensive Epilepsy Center, Department of Neurology, Thomas Jefferson University, Philadelphia, PA, USA
37. Department of Neurology-Biomagnetism rtd University Hospital Erlangen ,Germany
38. Department of Neurology, Mayo Clinic, Jacksonville, Florida USA
39. Department of Neurology, AIIMS, Delhi , India
40. Department of Neurology and Neurosurgery. Universidade Federal de São Paulo, São Paulo, Brazil.
41. Department of Clinical Neurosciences, University of Calgary, Canada
42. Department of Pediatric Neurology, Red Cross War Memorial Children’s Hospital, Neuroscience Institute, University of Cape Town, South Africa. https://orcid.org/0000-0001-7328-1796
43. Department of Neurology, West China Hospital of Sichuan University, Chengdu 610041, Sichuan, China
44. University College London NIHR BRC Great Ormond Street Institute of Child Health, Great Ormond Street Hospital & Young Epilepsy, UK, member of European Reference Network EpiCARE

**Abstract**

The International League Against Epilepsy (ILAE) has updated the operational classification of epileptic seizures, building upon the framework established in 2017. This revision, informed by the implementation experience, involved a working group appointed by the ILAE Executive Committee. Comprising 37 members from all ILAE regions, the group utilized a modified Delphi process, requiring a consensus threshold of over two-thirds for any proposal. Following public comments, the Executive Committee appointed seven additional experts to the revision taskforce to address and incorporate the raised issues, as appropriate. The updated classification maintains four main seizure classes: Focal, Generalized, Unknown (whether focal or generalized) and Unclassified. Taxonomic rules distinguish classifiers, which are considered to reflect biological classes and directly impact clinical management, from descriptors, which indicate other important seizure characteristics. Focal seizures and those of unknown origin are further classified by the patient's state of consciousness (impaired or preserved) during the seizure, defined operationally through clinical assessment of awareness and responsiveness. If the state of consciousness is undetermined, the seizure is classified under the parent term – the main seizure class (focal seizure or seizure of unknown origin). Generalized seizures are grouped into absence seizures, generalized tonic-clonic seizures and other generalized seizures, now including recognition of negative myoclonus as a seizure type. Seizures are described in the basic version as with or without observable manifestations, while an expanded version utilizes the chronological sequence of seizure semiology. This updated classification comprises four main classes and 21 seizure types. Special emphasis was placed on ensuring translatability into languages beyond English. Its aim is to establish a common language for all healthcare professionals involved in epilepsy care, from resource-limited areas to highly specialized centers, and to provide accessible terms for patients and caregivers.

**Key Points**

- The ILAE has updated the operational classification of epileptic seizures.
- Adjustments were based on experience with the clinical implementation of the classification established in 2017.
- The four main classes are: Focal, Generalized, Unknown whether focal or generalized and Unclassified.
- Consciousness is a classifier, and it is operationally defined by awareness and responsiveness.
- Seizures are described as with or without observable manifestations (basic) or by the chronological sequence of semiology (expanded).

**Introduction**

The ILAE operational classification of seizure types was published in 2017^1^. The paper concluded with a statement suggesting that the application of this classification in the field for a few years will prompt minor revisions and clarifications. The ILAE actively promoted the implementation of the 2017 classification, sparking intense debates within the international epilepsy community^2-6^.

In 2023, the ILAE's Executive Committee appointed a working group assigned to assessing the real-world application of the 2017 seizure classification and recommending adjustments while preserving the fundamental framework of the 2017 classification. The basic organization of the 2017 classification evolved from the original 1981 version^7^ through subsequent modifications. The primary objective remains the establishment of a common language and standardized definitions for clinical practice. Emphasizing flexibility, the classification aims to accommodate diverse clinical settings, including resource-limited areas and highly specialized centers. Simultaneously, it seeks to offer a clear and robust structure for implementation in research databases and clinical trials.

This seizure classification does not encompass neonatal seizures, which are addressed in a separate position paper^8^. Additionally, a new definition of acute symptomatic seizures^9^ and the nosology of status epilepticus^10^ have been allocated to other working groups. Notably, this classification specifically encompasses clinical seizures, omitting those events solely identified by electrographic activity.

The working group, appointed by the ILAE's Executive Committee, comprised a diverse and inclusive international representation. The methodology employed three successive steps: firstly, the identification of strengths and weaknesses within the 2017 classification; secondly, proposing adjustments and updates; and finally, engaging in an iterative Delphi process to attain a broad consensus. The updated version was made available on the ILAE website for a two-month period to receive public comments, subsequently undergoing successive revisions. In parallel, the paper was submitted to Epilepsia for review. A revision taskforce, composed of equal parts original and new members, was appointed by the ILAE to revise the proposal based on the comments. The final version was approvedby the ILAE's Executive Committee.

**Methods**

The working group

In January 2023, the Executive Committee appointed a working group comprising 37 experts, with a balanced representation of 19 women and 18 men. The group intentionally mirrored the diverse composition of the ILAE, incorporating members from all ILAE Regions: North America (7), Latin America (5), Europe (11), Eastern Mediterranean (2), Asia Oceania (9), and Africa (3). Within the group, 23 experts specialized in adult epileptology, while 13 primarily focused on pediatric epileptology. Additionally, one member brought expertise as a neurosurgeon. To ensure continuity, four members were selected from the task force involved in developing the 2017 version.

The working group conducted three workshop meetings: two were conducted entirely online in April and May 2023, while one meeting adopted a hybrid approach, combining face-to-face and online elements, held in September 2023 in Dublin, Ireland. Communication primarily occurred electronically, utilizing emails and an online work management platform (Monday.com). Video recordings and comprehensive documentation of the entire process were electronically archived and made accessible to all members throughout the duration of the process. The ILAE office provided technical assistance with the process.

Systematic review

We conducted a systematic review^11^ to evaluate the strengths and weaknesses of the 2017 ILAE seizure classification. We searched PubMed and Embase databases for articles addressing the implementation of the 2017 ILAE seizure classification. Eligibility criteria were: 1) research papers investigating applicability and feasibility of the 2017 seizure classification; 2) review and opinion papers. For the first criterion, we included congress abstracts too, if they provided sufficient details for evaluation. For the second criterion, we excluded congress abstracts and reviews by the authors of the 2017 classification.

Supplementary Document 1 displays the PRISMA flow diagram depicting the review process. Two authors (SB and ET) independently reviewed and rated the records, resolving any disagreements through consensus discussions. Subsequently, the working group further reviewed and edited the outcomes. The review encompassed a total of 41 articles, as detailed in Supplementary Document 2. Among these, 22 research articles evaluated the applicability and feasibility of the 2017 classification: nine studies supported its feasibility, 11 studies found it partially feasible, and two studies deemed it unfeasible. Additionally, 19 articles comprised reviews and opinions: 10 papers expressed negative critiques, six held neutral positions with an optimistic outlook for future implementations, while three presented mixed opinions—supportive and critical.

Strengths and weaknesses

We clustered strengths and weaknesses extracted from the systematic review (Supplementary Document 2) alongside additional input provided by the working group members.

Overall, the 2017 seizure classification's strengths lie in its operational approach and basic organization of seizure types, divided into four main classes. It offers flexibility for classification at varying levels of complexity, making it more practical for real-world clinical use. The addition of the "unknown" class was perceived as an improvement, enhancing the feasibility and applicability of the classification system^12-14^. There were differing opinions on the introduction of the term "focal to bilateral tonic-clonic seizure." However, a prospective study demonstrated that this term facilitated more accurate classification of seizures compared to its synonym in the older version (1981) of the classification system^15^. The inclusion of more descriptors was seen as a strength, particularly for implementation in databases^16^. A study validated the usefulness of distinguishing focal from generalized epileptic spasms^17^.

A robust debate occurred regarding the suitability of the term *awareness* to classify seizures, rather than using the term *consciousness*. Several papers pointed out the disadvantages of using awareness as a surrogate marker for *consciousness*^18-21^. Conversely, the appropriateness of the concept of *consciousness* in epileptology has also been critically discussed, given the challenges of reliably assessing it during a seizure (Gloor et al. Epilepsia 1986). *Impaired consciousness* is a commonly used medical term, broadly implemented in clinical neurology (Edlow et al., 2020; Giacino et al., 2018; Laureys et al., 2015; Posner et al., 2019). It is operationally defined by awareness and responsiveness (Alnagger et al., 2023; Posner et al., 2019)^22^. Based on the concepts of Gerorge Berkeley (1685-1753), William James (1842-1910), and Arthur Schopenhauer (1788-1860), Gloor identified important components of consciousness to include the “ability to attend and perceive, to relate perception to one’s own fund of personal memories, to remember recent events and to react voluntarily in response to such stimuli” (Gloor, Epilepsia 1986). There has been much progress in recent years in understanding mechanisms and developing tools for objectively measuring normal consciousness (Seth and Bayne, 2022; Storm et al., 2017) and impaired consciousness in neurological disorders (Fischer and Edlow, 2024; Giacino et al., 2004; Giacino et al., 2018; Laureys et al., 2015), including epilepsy (Arthuis et al., 2009; Bauerschmidt et al., 2013; Beniczky et al., 2016; Cavanna et al., 2008; Englot et al., 2010; Guo et al., 2016; Lambert and Bartolomei, 2020; Wheeler et al., 2025). For general neurologists, an epileptic seizure is a differential diagnosis within conditions of transient loss or impairment of consciousness^23^. For medical students and similarly to lay persons, *consciousness* is simply explained as the ability to respond and to remember^24^. The debate against using responsiveness as a classification criterion revolves around its dependence on intact motor functions and its difficulty in outpatient settings, although studies indicate that impaired responsiveness is often reported during patient history-taking^21^. In epilepsy monitoring units, responsiveness is frequently evaluated over awareness^20^. Some clinicians have adopted the term *impaired awareness* to denote impaired responsiveness, believing it aligns with ILAE position paper, despite this interpretation being incorrect^21^. In children under 4-5 years, assessing awareness is often challenging or impossible^25^, whereas responsiveness can be evaluated using age-appropriate methods^26^. A crucial consideration lies also in the translatability of these terms: *awareness* faces challenges in translation across languages such as Spanish, French, Portuguese, and German^3^, while *consciousness* is more translatable and already a universally accepted medical term.

The clinical relevance of categorizing seizures into motor vs. nonmotor and utilizing the first observed phenomenon as a classifier have been questioned. In contexts such as clinical trials or resource-limited settings, a more practical dichotomy, “with versus without observable manifestations”, has been considered more beneficial^27^. Notably, nonmotor seizures may exhibit observable manifestations such as aphasia or flushing. The use of the first semiology phenomenon as a classifier has shown limited clinical relevance. It does not influence critical factors such as the selection of antiseizure medication, prognosis, or the localization of seizures for surgical therapy^2-4-5, 19, 28-29^. A more clinically relevant approach for characterizing the epileptic network, especially in the context of presurgical evaluation and clinic-anatomic correlation, involves describing the seizure evolution, specifically, the chronological sequence of semiology phenomena^2-4,28,30-33^.

The 2017 classification categorized absences as nonmotor seizures, which is misleading. Typical absence seizures often present observable motor phenomena such as discrete automatisms, head retropulsion, and eye blinks, while atypical absences may involve atonic phenomena^6^. Notably, motor manifestations are characteristic features of specific absence seizure types, such as eyelid myoclonia with absence and myoclonic absences^34^.

Epileptic negative myoclonus is a well-documented phenomenon^35^ acknowledged in both the earlier^36^ and the revised version of the ILAE semiology-glossary^37^. It is important to note that epileptic negative myoclonus differs from asterixis found in toxic-metabolic encephalopathies. While discussed in prior works, negative myoclonus was not included in the 2017 classification^35^.

Experimental studies in animal models^38^ and humans^39^ demonstrated the focal onset in generalized seizures^40-43^, and this has been incorporated in the current ILAE definitions^1,44^. The term “generalized onset seizure” seems to be in contradiction with this, and it may be misleading in the clinical practice, since focal onset of generalized seizures was well-documented in large survey studies^43^, and video-EEG recordings^46-48^.

Epileptic seizures can be classified using various principles, potentially resulting in numerous seizure-types, some of which might be redundant and lack clinical relevance. Establishing clear taxonomic rules is essential to precisely define and differentiate classifiers (used to identify seizure types) from descriptors (used to characterize specific features within a seizure type).

Proposed adjustments

Building upon the strengths and weaknesses discussed and clustered in the previous section, the working group formulated proposals for adjustments. These proposals were later modified during the Delphi process and subsequent revision, as detailed below.

1. *Taxonomic rules*: We distinguish Classifiers, which reflect biological classes (conceptual justification) and directly impact clinical management (utilitarian justification), from Descriptors, which represent key seizure characteristics and indirectly aid patient management when combined with other clinical data. Main seizure classes, seizure types and level of consciousness are classifiers, while semiology features are descriptors..

2. *Terminology of the main seizure classes*: change “generalized-onset seizure” to “generalized seizure”, change “focal-onset seizure” to “focal seizure”, and change “unknown onset seizure” to “unknown whether focal or generalized”.

3. *Level of consciousness* is also a classifier for focal seizures and for seizures of unknown origin. We propose to substitute awareness (aware or impaired awareness) with consciousness (preserved or impaired), operationally defined based on awareness and responsiveness.

*4. Descriptors*: We propose to replace the motor versus nonmotor sub-classification within focal seizures and within seizures unknown whether focal or generalized, with a distinction between seizures with observable manifestations and those without, in the basic version of the classification. In the expanded version, describe seizure semiology in chronological sequence, depicting the sequence of seizure phenomena.

*5. Epileptic negative myoclonus*: Include the recognition of epileptic negative myoclonus within the seizure classification.

*6. Generalized seizures*: Remove “nonmotor” when categorizing absence seizures.

*7. Epileptic spasms*: Incorporate epileptic spasm as a semiology descriptor for focal seizures and for seizures unknown whether focal or generalized. Retain epileptic spasms as a seizure type for generalized seizures.

Delphi method

We employed a modified Delphi method^49^ to achieve consensus regarding the proposed adjustments and the update of the seizure classification. For a proposal to pass, it required at least a 2/3 majority vote from the group. Acting as moderators, two authors (SB and ET) facilitated the process. They gathered and summarized the votes, incorporating comments, and returned them for the subsequent round, refraining from voting themselves. Throughout the process, thirty-five members of the working group participated in voting. Individual responses were anonymized to other participants, but after each round, they received a summary of results, along with incoming comments and suggestions.

Consensus was achieved after seven rounds. The first three Delphi rounds focused on addressing the proposals, while the subsequent four rounds were dedicated to the entire updated classification system. All implemented proposals garnered more than 2/3 of the votes, and the final version received unanimous approval from all members of the working group.

Public comments and revision

The proposed position paper was reviewed by the ILAE Executive Committee for approval of its concept and content and was posted on the ILAE website for public comments from August 12 to October 16, 2024. A total of 44 comments were received from chapter representatives and individual members, in addition to the anonymous peer reviews in *Epilepsia*.

The Executive Committee appointed a revision taskforce to review and incorporate these comments as appropriate. Chaired by Elaine Wirrell, the revision taskforce included seven new members appointed by the Executive Committee and seven members from the original working group (see Supplementary Document 3). The taskforce categorized the comments by topic and distinguished between supportive/approving and critical/disapproving comments (see Supplementary Document 4).

Overall, the feedback was positive, with 25 supportive comments, two peer reviews, and five critical comments (see Supplementary Document 3). The main criticisms were that the proposed changes were introduced too soon, too quickly, and represented too much change. The revision taskforce and the Executive Committee considered the eight-year interval appropriate for updates based on experience with implementing the 2017 classification, which had not been tested in real-world practice beforehand. This timeframe aligns with practices in other medical societies and previous epilepsy classifications (e.g., the 1985 classification, revised in 1989). Delaying necessary updates would likely make future implementation more challenging. Community feedback, as evidenced by the systematic literature review, highlighted the need for changes. These updates followed the robust procedure recently adopted for ILAE position papers, incorporating published evidence and real-world experience. The goal of the revision taskforce was to ensure the changes were accurate. Since the framework and main terms remain unchanged, the updated seizure classification aligns with the 2017 classification’s overarching concept.

Another frequently debated aspect was the return to using consciousness instead of awareness. Most comments supported this change, emphasizing that consciousness is a widely accepted and translatable medical term, operationally defined through the assessment of responsiveness and recall. Responsiveness, often part of patient history, is assessable even in young children, where awareness may not be applicable^26^. Since the 2017 classification, describing a non-responsive patient as having “impaired awareness” became widespread but is inaccurate. Moreover, using an alternative (“surrogate”) term for consciousness distances epilepsy classification from broader medical standards - an epileptic seizure is a key element in the differential diagnosis of transient impairment of consciousness. Concerns were raised that impairment of consciousness might be misunderstood by patients and caregivers as total loss of consciousness. However, when taking a history, health professionals should ask about responsiveness and recall during the seizure, then draw conclusions about consciousness themselves, rather than relying on patients or caregivers to label it as impaired or preserved. The medical term consciousness can then be explained to patients and caregivers as the ability to respond and recall during the seizure.

To aid in the correct classification of epileptic spasms, a decision-flowchart figure and a detailed explanation were added to the revised paper. All changes made by the revision taskforce to the working group’s original proposal are summarized in Supplementary Document 4. The revised position paper was submitted to the ILAE Executive Committee for final approval.

**Results**

The fundamental framework for classifying epileptic seizures is maintained^1,7^. The main seizure classes include **Focal, Generalized, Unknown whether focal or generalized, and Unclassified.** Figures 1 and 2 illustrate the basic and expanded seizure classifications, and Table 1 presents the taxonomic hierarchy of seizure classification. ***Classifiers*** define the seizure types, considered as biological classes with direct influence on patient management by guiding syndrome diagnosis, therapeutic decisions and prognosis. ***Descriptors***, on the other hand, are important clinical characteristics of the seizures that, along with other clinical data and modalities, indirectly contribute to shaping patient management. Descriptors are essential for clinical decisions and, in specific contexts, may significantly influence therapy (e.g., epileptic spasm or myoclonus in the context of a focal seizure).

Focal seizures are defined as originating within networks limited to one hemisphere^1,44^. They may be discretely localized or more widely distributed, may originate in cortical or subcortical structures. For each seizure type, ictal onset is consistent from one seizure to another, with preferential propagation patterns that may involve the contralateral hemisphere. In some cases, however, there is more than one network, and more than one seizure type, but each individual seizure type has a consistent site of onset^44^. Focal-to-bilateral tonic-clonic seizures^1^ are focal seizures in which the ictal activity propagates to both hemispheres, while the semiology evolves to impairment and eventually complete loss of consciousness and bilateral tonic muscle activation, followed by a clonic phase with progressive decrease in frequency, due to a gradual increase in the duration of the silent periods interrupting the tonic muscle activity^37^.

Generalized seizures are defined as originating at some point within, and rapidly engaging, bilaterally distributed networks, which can include cortical and subcortical structures, but not the entire cortex^1,44^. Seizure onset can appear localized, and seizures can be asymmetric.

When there is information available to characterize certain aspects of seizures, but it is insufficient for a clear classification as focal or generalized, they are categorized as “Unknown whether focal or generalized”. In cases where there is no available information to characterize the seizure, but the clinician is confident that the event is an epileptic seizure, it is labeled as “Unclassified”^1^. Subsequently, as more information becomes available to the clinician, these seizures can be re-classified as either focal or generalized.

**Figure 1. The basic version of the updated seizure classification**


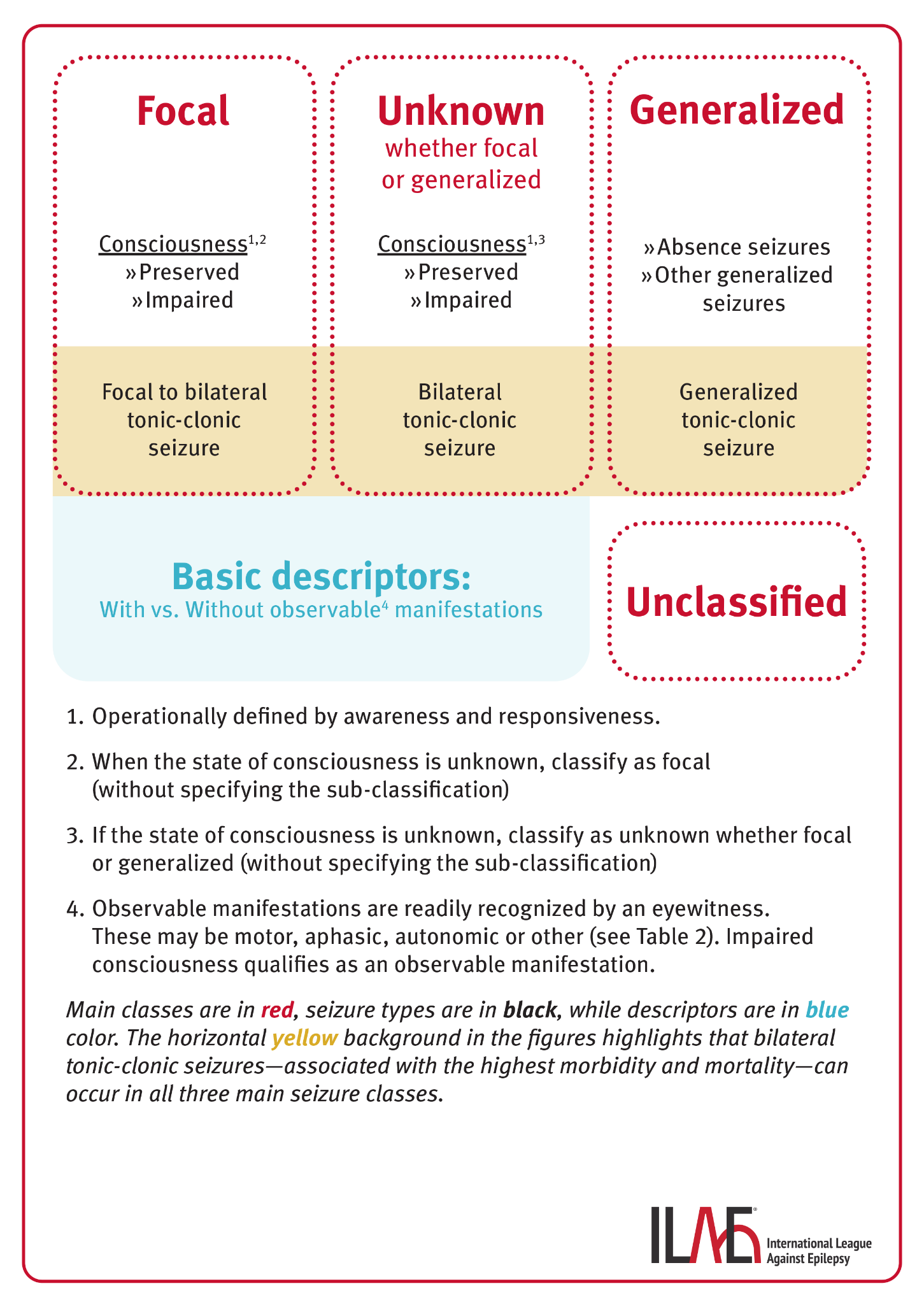


Focal seizures and seizures unknown whether focal or generalized are further classified according to the patient´s state of consciousness during the seizure: impaired or preserved. If the state of consciousness is undetermined, the seizure is classified under the parent term (focal seizure or seizure of unknown origin). Consciousness is operationally defined by establishing awareness and responsiveness, relying on information obtained from medical history^21^ or through behavioral testing by medical personnel^26^. These operational terms are explained to the patients and caregivers as the ability to remember and to respond appropriately and normally during the seizure. Rather than asking patients and caregivers about consciousness, it is advisable to ask specifically about recall of the events (awareness) and degree of responsiveness during the seizure. An inadequate response or a significantly longer response latency compared to the interictal (baseline) state qualifies as impaired responsiveness^26,37^. Patients and caregivers may need to be reminded that consciousness can still be impaired though the eyes are open, and the patient attempts to interact. In real-world scenarios, information may be available only about one of these characteristics (awareness or responsiveness). If either is impaired in any way, the seizure is classified as impaired consciousness. It is important to exercise caution and consider isolated epileptic amnesia as a potential cause for the lack of recall of ictal experiences, and to rule out ictal paresis or ictal receptive aphasia as potential causes of unresponsiveness, whenever possible.

**Figure 2. The expanded version of the updated seizure classification**


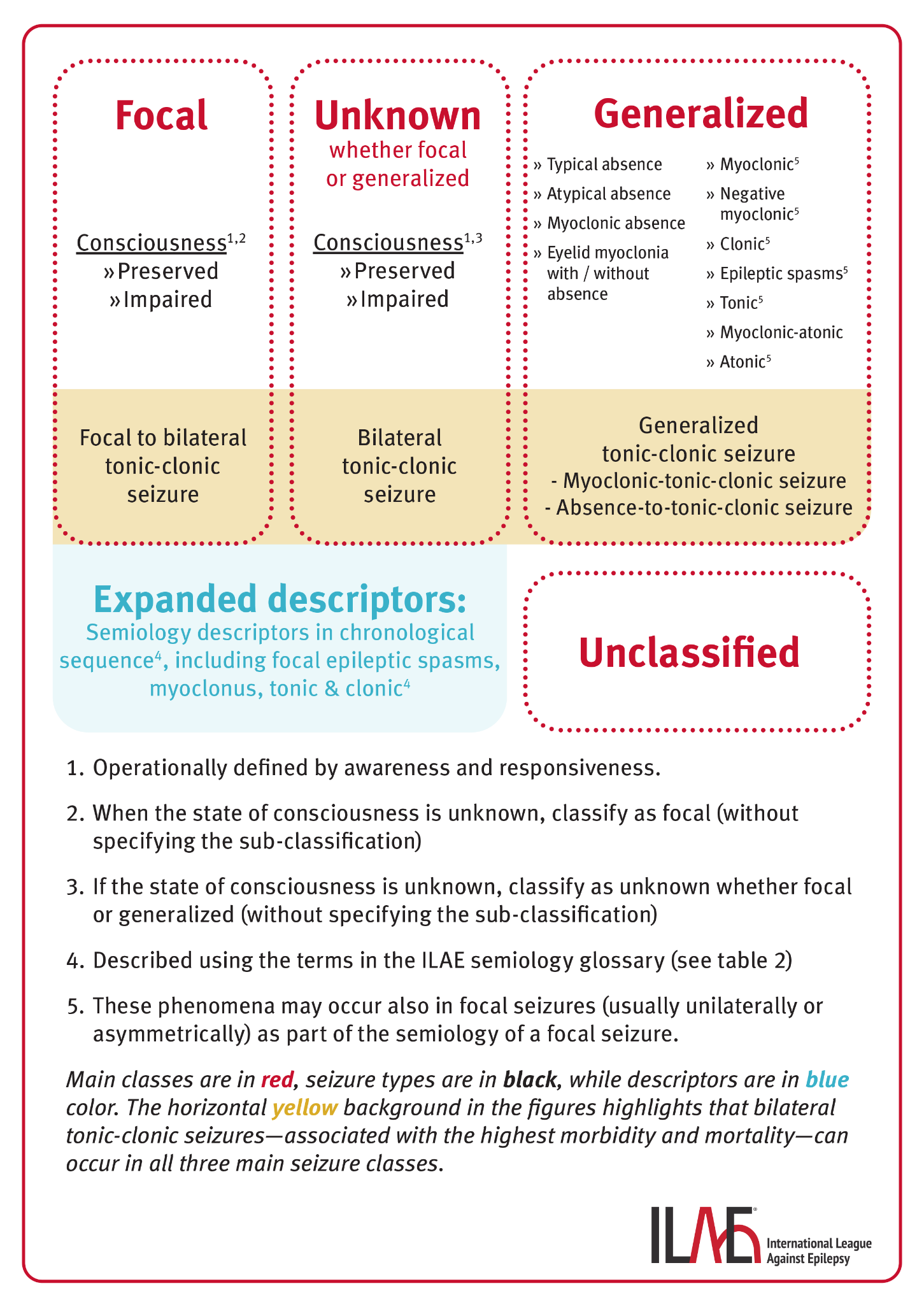


Descriptors can be employed to provide additional characterization of seizures. In the basic version, a straightforward dichotomy is utilized: seizures are described as either having observable manifestations or not. Observable manifestations are easily identified by eyewitnesses^27^, are non-volitional and can include motor, aphasic, autonomic, or other features (see Table 2).. In the expanded version, seizures are described in detail, by listing in chronological order the semiology features (see Table 2) that occur during the seizure^37,50^. The sequence is indicated by arrows pointing in the direction of seizure evolution (for example: epigastric aura 🡪 right hand automatism 🡪 impaired responsiveness + impaired awareness). All items in the table outlining the semiology features (Table 2) are defined and their significance is explained in detail in the ILAE glossary of seizure semiology^37^. Additionally, video examples are available for each item^37^. The ictal evolution offers crucial insights, as it can identify specific conditions, such as epilepsy of infancy with migrating focal seizures^51^, and aid in the localization of the cortical areas generating the seizures^37^. Please note that colloquial terms derived from semiology, such as hyperkinetic (or hypermotor) seizures, focal spasms, focal myoclonic seizures, focal clonic seizures, and focal tonic seizures, refer to focal seizures as the main seizure type.

The descriptors are based on seizure semiology. We acknowledge the importance of other clinically relevant seizure characteristics, such as the context of occurrence (e.g., reflex or sleep-related) and the anatomical localization of the epileptogenic zone. Although these characteristics are not formally included in the seizure classification, they are valuable in clinical practice and research.

In the basic version of seizure classification, generalized seizures are grouped into absence seizures, generalized tonic-clonic seizures and other generalized seizures. The latter is a grouping term, not a defined concept. In the figures illustrating seizure classification, tonic-clonic seizures are positioned at the end of each main class: focal-to-bilateral tonic-clonic seizures, generalized tonic-clonic seizures, and bilateral tonic-clonic seizures of unknown origin (whether focal or generalized). This placement highlights these seizure types, which are associated with the highest morbidity and mortality, and are the major risk factor for sudden unexpected death in epilepsy^52-55^. In the expanded seizure classification, all generalized seizure types are listed (Figure 2 and Table 1). Definitions of all generalized seizure types are provided in Supplementary Document 5.

It is acknowledged that generalized tonic-clonic seizures may be heralded by myoclonic jerks or an absence seizure, a distinction reflected in the subtypes of this seizure^1,56-57^. If these specific features (myoclonic jerks or absence at onset) are not observed, the seizure is classified under the parent term, generalized tonic-clonic. Generalized negative myoclonus is now recognized as a distinct seizure type, while the other generalized seizure types remain consistent with the 2017 classification^1^. Generalized tonic seizures may be preceded or followed by spasms (colloquially termed “*tonic spasms*”), a myoclonic jerk (“*myoclonic-tonic seizure*”), or a hyperkinetic seizure followed by a spasm (“*hypermotor-tonic-spasms*”). While evidence suggests that some of these combinations of seizure-types may be relevant for syndromic diagnosis (for example, hypermotor-tonic-spasms in CDKL5 deficiency disorder), they are not yet formally included in the seizure classification. Further research is needed to establish the clinical significance of these tonic seizure subtypes.

Epileptic spasms represent an important ictal phenomenon (Table 2), and their early recognition and accurate classification is essential for optimal treatment^1,51^. While spasms can be generalized, focal or unknown whether focal or generalized, the most critical aspect in infants is timely recognition of this unique seizure type and initiation of spasms-specific therapies, as delay can result in poorer developmental outcomes. Indeed, discerning whether spasms are focal or generalized can be challenging (Figure 3) and require a multimodal approach^51^. Within the generalized main class, epileptic spasms are a *classifier*, often associated with infantile epileptic spasm syndrome (IESS)^51^. In the focal and unknown classes, epileptic spasms are a *descriptor* and thus described within the seizure semiology (e.g., focal epileptic spasm). In the context of the clinical data (including age of onset), they lead to the syndromic diagnosis of IESS^51^, and pharmacological therapy specific for this syndrome must be initiated without delay. Furthermore, in cases of focal epileptic spasms (unilateral or asymmetric semiology) or when other findings, such as neuroimaging, suggest a focal origin, early surgical treatment should be considered (Figure 3). Epileptic spasms can also occur in older age groups, outside IESS, which led to the terminology shift from infantile spasms to epileptic spasms^1^. In these cases, the pharmacological treatment differs from IESS (Figure 3). Besides epileptic spasms, other motor ictal phenomena, including myoclonus, clonus, and tonic muscle contractions can occur in both generalized seizures (defining the seizure type) and in focal seizures where they typically present unilaterally or asymmetrically as part of the focal seizure semiology (Figure 2).

**Figure 3**. Decision flowchart for classifying epileptic spasms and their relevance to syndromic diagnosis and treatment.


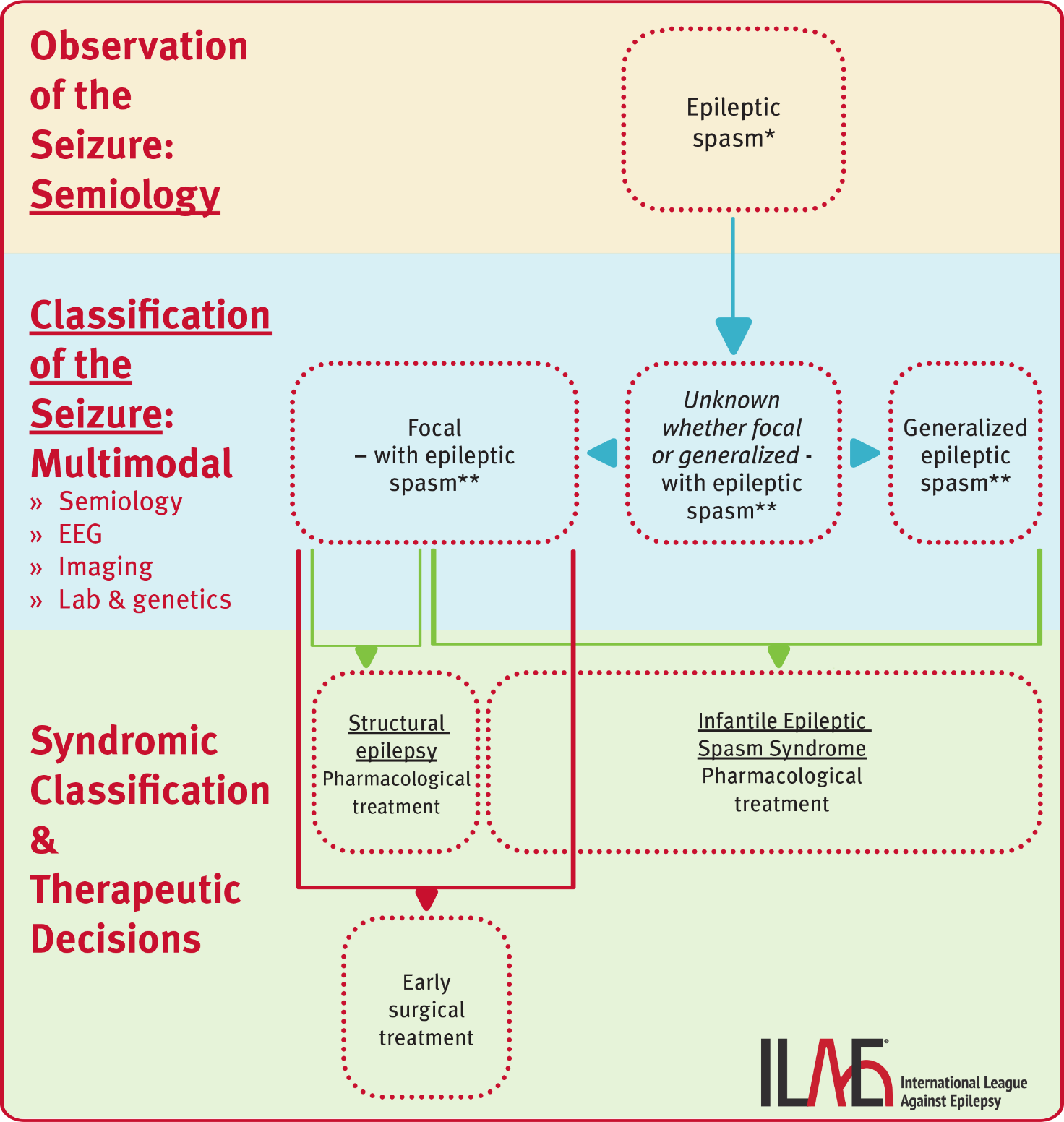


Epileptic seizures are classified within a taxonomic hierarchy, comprising main classes and seizure types. (Table 1). We found it important to explicitly outline the specific list of seizure types, following the principles illustrated in the figures and detailed in this paper. The table aims to provide clear guidance for electronic databases. The seizure classification includes four main classes and 21 seizure types a significant simplification compared to the 2017 edition, which included 63 seizure types^5, 58^. The updated classification retains the flexibility of the 2017 edition. The classification of an individual seizure can halt at any level on the hierarchical tree, and seizures initially labeled as unknown or unclassified can be later reclassified, as new information about the seizure becomes available.

**Table 1. The taxonomic hierarchy of epileptic seizure classification.** Classifiers are shown in black, while descriptors are in blue color. Main classes are indicated in bold font, seizure types are underlined.

| 1. **Focal (F)**    1. Focal preserved consciousness seizure (FPC)    2. Focal impaired consciousness seizure (FIC)    3. Focal-to-bilateral tonic-clonic seizure (FBTC)  \| ***Descriptors***   - *Basic:*   - *With observable manifestations*   - *Without observable manifestations* - *Expanded:*   - *Semiology descriptors in chronological sequence:*   *Semiology (glossary*) + Somatotopic modifiers* \| \| --- \|  1. **Unknown whether focal or generalized (U)**    1. Unknown whether focal or generalized - preserved consciousness seizure (PC)    2. Unknown whether focal or generalized - impaired consciousness seizure (IC)    3. Unknown whether focal or generalized - bilateral tonic-clonic seizure (BTC)  \| ***Descriptors***   - *Basic:*   - *With observable manifestations*   - *Without observable manifestations* - *Expanded:*   - *Semiology descriptors in chronological sequence:*   *Semiology (glossary*) + Somatotopic modifiers* \| \| --- \|  1. **Generalized (G)**    1. Absence seizures (AS)       1. Typical absence seizure (TA)       2. Atypical absence seizure (AA)       3. Myoclonic absence seizure (MA)       4. Eyelid myoclonia with / without absence (EMA)    2. Generalized tonic-clonic seizure (GTC)       1. Myoclonic tonic-clonic seizure       2. Absence-to-tonic-clonic seizure    3. Other generalized seizures**       1. Generalized myoclonic seizure (GM)       2. Generalized clonic seizure (GC)       3. Generalized negative myoclonic seizure (GNM)       4. Generalized epileptic spasms (GES)       5. Generalized tonic seizure (GT)       6. Generalized atonic seizure (GA)       7. Generalized myoclonic-atonic seizure (GMA) 2. **Unclassified** |
| --- | --- | --- |


*See table 2 with the semiology features.

**This is a grouping term, not a defined concept.

**Table 2. Descriptors for Focal seizures and for seizures Unknown whether Focal or Generalized**

| **Somatotopic modifiers**  *Side (left, right, bilateral-symmetric, bilateral-asymmetric) + Body part* | |
| --- | --- |
| **Semiology features** | |
| **1. Elementary motor phenomena^*^**  Akinetic  Astatic  Atonic  Clonic  Dystonic  Epileptic nystagmus  Epileptic spasm  Eye blinking  Eye deviation  Gyratory  Head orientation  Ictal paresis  Myoclonic  Myoclonic-atonic  Epileptic negative myoclonus  Tonic (focal tonic, chapeau de gendarme, fencing posture)  Tonic-clonic (Figure-of-four)  Versive  _______________________________________________  **2. Complex motor phenomena^*^**  Automatisms  - Gestural automatisms-distal  - Gestural automatisms-genital  - Gestural automatisms-proximal  - Ictal grasping  - Mimic automatisms (Gelastic, dacrystic)  - Oro-alimentary automatisms  - Verbal automatisms  - Vocal automatisms  Hyperkinetic behavior  _______________________________________________  **3. Sensory phenomena^**^**  Auditory  Body-perception illusion  Depersonalization  Gustatory  Olfactory  Somatosensory  - painful  - non-painful  Vestibular / Dizziness  Visual  _______________________________________________  **4. Cognitive & language phenomena^#^**  Aphasia  Confusion / disorientation  Dysmnesia  - Amnesia  - Déjà vu/déjà vécu/ jamais vu/ dreamy state /reminiscence  Forced thinking  Other focal cognitive deficits (e.g., anosognosia, apraxia, neglect) | **5. Autonomic phenomena^#^**  Cardiovascular  - Ictal asystole  - Ictal bradycardia  - Ictal tachycardia  Cutaneous / thermoregulatory  - Flushing  - Piloerection  - Sweating  Epigastric  Gastrointestinal  - Borborygmi  - Flatulence  - Hypersalivation  - Nausea / Vomiting  - Polydipsia  - Sialorrhea  - Spitting  Pupillary  - Miosis  - Mydriasis  Respiratory  - Apnea  - Choking  - Hyperventilation  - Hypoventilation  Urinary  - Incontinence  - Urinary urge  _______________________________  **6. Affective (emotional) phenomena^#^**  Anger  Anxiety  Ecstatic/bliss  Fear  Guilt  Mirth  Mystic  Sadness  Sexual  _______________________________  **7. Indescribable aura^**^**  **_______________________________**  **Postictal phenomena**  Autonomic signs  Blindness (hemianopsia or amaurosis)  Confusion  Headache  Language dysfunction  Nose-wiping  Palinacousis  Paresis (Todd´s paresis)  Psychiatric signs  Unresponsiveness |

*Observable manifestations; **Not observable manifestations; # Possibly observable manifestations. If phenomena not listed above occur during the seizure, they are added in free text. Awareness and responsiveness define consciousness and hence are classifiers. All items in this table are defined in the ILAE glossary of semiology.

While the updated seizure classification places significant emphasis on seizure semiology and can be applied in resource-limited settings, similar to the 2017 edition, it remains interpretative. This allows for the incorporation of supplementary data to identify the seizure types^1^. In alignment with clinical practice, it is recommended to classify seizures by considering all available information, encompassing semiology and supportive data such as EEG, neuroimaging, laboratory results, and genetics.

In the following section, we illustrate the implementation of the updated seizure classification, utilizing examples from the previous edition and from the articles which criticized it^4,59-60^.

A young woman awakens to find her 20-year old boyfriend having a seizure in bed. The onset is not witnessed, but she is able to describe bilateral stiffening followed by bilateral “shaking”. EEG and magnetic resonance imaging (MRI) findings are normal. This seizure is classified as Bilateral tonic-clonic seizure - unknown whether focal or generalized (BTC; 2.3).

In an alternate scenario of the previous case, the EEG shows a clear right parietal slow-wave focus. The MRI shows a right parietal region of cortical dysplasia. In this circumstance, the seizure is classified as Focal-to-bilateral tonic–clonic seizure (FBTC; 1.3).

A 25-year-old woman describes seizures beginning with 30 seconds of an intense feeling that “familiar music is playing.” She can hear other people talking, but afterwards realizes that she could not determine what they were saying. Eyewitnesses report that the patient does not respond to external stimuli during the seizure – neither verbal nor tactile (touching the patient). After an episode, she is mildly confused, and has to “reorient herself.” The seizure is classified as Focal impaired consciousness seizure (FIC; 1.2.) with : auditory aura 🡪 receptive aphasia 🡪 impaired responsiveness 🡪 postictal confusion.

A 22-year-old man has seizures during which he remains fully aware, with the “hair on my arms standing on edge” and a feeling of being flushed. These are classified as Focal preserved consciousness seizure (FPC) with observable manifestations: piloerection + flushing.

A 13-year-old with juvenile myoclonic epilepsy has seizures beginning with a few jerks, followed by stiffening of all limbs and then rhythmic jerking of all limbs. These are classified as Generalized myoclonic-tonic-clonic seizures (GTC; 3.2.1..)

A 3-month-old boy has clusters of short seizures with flexion in the neck and hips, and abduction in the shoulders of short duration (up to 2 s). The patient has 3-15 clusters per day. The child was encephalopathic, without developmental progression. Seizures were resistant to multiple antiseizure medications, including ACTH. Repeated MRI was unrevealing. Video-EEG showed epileptic spasms associated with a generalized suppression on EEG. The seizure is classified as Generalized epileptic spasm (GES; 3.3.4).

A 14-month-old girl has sudden extension of both arms and flexion of the trunk for about 2 s. These seizures repeat in clusters. EEG shows hypsarrhythmia with bilateral spikes, most prominent over the left parietal region. MRI shows left parietal cortical dysplasia. Because of the ancillary information, the seizure is classified as Focal seizure (F; 1.) with observable manifestations: epileptic spasms (brief version: Focal epileptic spasms).

During long-term video-EEG monitoring, a 28-year-old female patient experiences an ascending sensation from the stomach and then starts chewing and manipulating nearby objects using the right hand. The patient can recall what happens during these episodes and is able to respond. The seizure is classified as Focal preserved consciousness seizure (FPC; 1.1) with observable manifestations: epigastric aura 🡪 oroalimentary automatisms + gestural automatisms with the right hand + preserved awareness and responsiveness.

The patient reports episodes starting with seeing colored dots and stripes on the left side. The patient cannot recall what happened after that, but eyewitnesses report that the patient does not respond to verbal and tactile stimuli, turns the head to the left, becomes stiff and then has jerks in all limbs. The seizure is classified as Focal-to-bilateral tonic-clonic seizure (FBTC; 1.3) with observable manifestations: elementary visual aura on the left side 🡪 versive to left + loss of awareness & responsiveness 🡪 bilateral tonic-clonic.

A 33-year-old, right-handed man experienced febrile seizures in infancy. Habitual, unprovoked seizures started at the age of 15 years and were accompanied by a feeling of abdominal discomfort followed by loss of awareness. His wife reported that about once a month he displays episodes of lip smacking, fumbling hand movements and occasional right-hand posturing. The seizure is classified as Focal impaired consciousness seizure (FIC; 1.2) with : epigastric aura 🡪 impaired awareness 🡪 oroalimentary automatisms + gestural automatisms + dystonic posturing in the right hand.

**Discussion**

The revised seizure classification adheres to the same framework as the 2017 version, maintaining the four main classes. In addition to the archetypical classes of focal and generalized seizures, two more main classes are included for practical reasons: “unknown” (for cases where the distinction cannot be made) and “unclassified” (a temporary class, when no further information is available about the seizure). The impetus for the update arose from the collective experiences after applying the 2017 seizure classification and an iterative discourse of the international epilepsy community. The 2017 version was anticipated to require adjustments based on the insights gained during its implementation in clinical practice.

The working group employed a robust, yet conservative methodology, based on a systematic analysis of the strengths and weaknesses of the 2017 version. Proposals for updates were only considered if they addressed a problem documented in the literature. Approval of any proposal required more than two-thirds of the votes in the Delphi process. The large working group represented the diversity of the ILAE, encompassing broad representation from all regions and various sub-specialties allowing for a broad discussion on the ontological relativity of the terms used in the 2017 classification and widely varying conceptual schemes in different languages. The proposal was posted for public comment, and a newly appointed taskforce revised the document based on relevant community feedback. Much like the 2017 edition, the primary objective was to establish a common language and framework for clinical practice. With a focus on flexibility, the classification aims to accommodate diverse settings, ranging from resource-limited areas to highly specialized centers. Simultaneously, it strives to offer a well-defined and clear structure, suitable for implementation in research databases and clinical trials.

Special emphasis was placed on ensuring the coherence and internal consistency of the classification. Following traditional principles employed in scientific classification systems, we established clear taxonomic rules derived from clinical and conceptual reasoning. Features directly impacting patient management were designated as classifiers, while other seizure characteristics served as descriptors. These were organized within the taxonomic hierarchy, resulting in four main classes and a total of 21 seizure types. The descriptors were structured into two layers: in the basic version, based on the dichotomy of observable ictal manifestations or the lack thereof, and in the expanded version, organized according to the chronological sequence of seizure semiology. The numbering in the taxonomic hierarchy list is designed to ensure consistency across databases and languages, mitigating any potential ambiguity.

To keep the classification system as simple as possible, we refrained from introducing neologisms. Instead, we utilized established medical terminology commonly found in literature and ensured translatability into languages beyond English. The classification has been translated into 14 languages (Supplementary document 6), providing a broad, global coverage: Arabic, Chinese, Danish, French, German, Hungarian, Italian, Japanese, Korean, Portuguese, Romanian, Russian, Spanish, Ukrainian. We aimed to create a system that is easily communicable to both patients and caregivers.

**Table 3. The key changes in seizure classification from 2017 to 2025**

| 1. “Onset” is removed from the names of the main seizure classes. 2. A distinction is made between classifiers and descriptors, based on taxonomic rule. 3. Consciousness is used as a classifier instead of awareness, with consciousness operationally defined by awareness and responsiveness. 4. The motor vs. non-motor dichotomy is replaced by observable vs. non-observable manifestations. 5. The chronological sequence of seizure semiology is used to describe seizures, rather than relying solely on the first sign. 6. Epileptic negative myoclonus is recognized as a seizure type. |
| --- |

The changes included in the updated seizure classification are summarized in Table 3. The term “onset” has been omitted from the names of the main seizure classes, as there is compelling evidence suggesting focal onset in generalized seizures as well^38-41,45-48^. The names of these classes now align with their definitions in the ILAE position papers^1,44^.

Both awareness and responsiveness are used to characterize consciousness, which is now the classifier. The motor vs. non-motor dichotomy was extended to observable vs. non-observable manifestations, which is deemed advantageous for clinical trials. This is now considered a descriptor in the basic version of the seizure classification. In the expanded version, the entire chronological sequence of seizure semiology is utilized for describing the seizure, rather than just the initial sign. This approach was considered more suitable for advanced settings, such as long-term video-EEG monitoring and presurgical evaluation.

The term ”non-motor” has been removed from absence seizures due to the presence of motor phenomena that may be observed during them, some of which are characteristic of certain types of absence seizures (e.g., myoclonic absence, eyelid myoclonia with absence). Negative myoclonus is now recognized as a seizure type. Within generalized seizures, epileptic spasm is considered a seizure type, while within focal seizures and seizures of unknown origin, epileptic spasm is described as part of the seizure semiology (e.g., focal epileptic spasm). Similarly, motor phenomena defining generalized seizure types (myoclonic, tonic, atonic) may also be part of the semiology of a focal seizure.

The updated classification maintains the continuity with the 2017 edition, so that seizures already classified with the previous version can easily be converted. For example, impaired awareness translates to impaired consciousness, a motor seizure is an observable manifestation.

These adjustments of the updated seizure classification were based on experience with the application of the 2017 version. They are relatively minor modifications that preserve the fundamental framework of seizure classification. The aim is to enhance broad clinical applicability across diverse settings, and consequently aid useability of the classification.

**Acknowledgements**

We would like to express our gratitude for translating the classification to Andriy Dubenko, Marina Nikanorova, Volodymir Kharytonov, Levente Hadady, Jozsef Janszky, Dae-Won Seo, Dae Lim Koo, Kyung Min Kim, Byung Chan Lim, Eun Yeon Joo, Hunmin Kim, and Kyoung Jin Hwang. We are thankful to Jason Ryan for the assistance with the graphical layout of the figures.

We are grateful to those who submitted comments during the public hearing: Sawsan Albazi, Markus Leitinger, Hannah Cock, Reza Azizimalamiri, Kimford Meador, Robert S. Fisher, Antonio Carlos Borges, Charles Akos Szabo, Chantal Depondt, Ana Mingorance, Marian Galovic, Gautam Gangopadhyay, Zulfi Haneef, Gwendoline Kandawasvika, Sandeep Patil, Angela Gnanadurai, Camila Hobi, Juan Carlos Resendiz, Andreas Schulze-Bonhage, Colin Dunkley, Pasquale Striano, Mahesh Kamate, Rodrigo Riquelme, Bert Kleine, Mario Mastrangelo, Jun Enriquez, Mahmoud Mohammadi, Reza Shervin Badv, Zahra Rezaei, Ricardo Lutzky Saute, M.A.Aleem, GP Burman, Gary Mathern, Ahmadjon Nishonov, Ashok Kumar, Katsumi Imai, Çiğdem Özkara, Gaetano Cantalupo, Dagoberto Cabrera Hemer, health professionals from Universidad de Antioquia, Jesly Hael Doria Atencia, Zarine Mogal;on behalf of the Pakistani chapter of the ILAE, John Dunne, Matthew Walker, Lynette Sadleir, Ernest R Somerville.

**Conflicts of interest:**

BJ serves as Associate Editor of the Journal Neurology. She receives research support from NIH, CDC and Neuropace, Inc.

DC received educational grant from UCB, Astra Zeneca, Desitin and is a member of the advisory board of Astra Zeneca and UCB. Dr Craiu is a Chair of the Education and Training Committee and of the Guidelines Committee of the EPNS (European Pediatric Neurology Society). No COIs related to this article.

ET has received personal honoraria for lectures, and educational activities from EVER Pharma, Marinus, Arvelle, Angelini, Alexion, Argenx, Medtronic, Biocodex, Bial-Portela & Ca, NewBridge, GL Pharma, GlaxoSmithKline, Boehringer Ingelheim, LivaNova, Eisai, Epilog, UCB, Biogen, Sanofi, Jazz Pharmaceuticals, Actavis; his institution received research grants from Biogen, UCB Pharma, Eisai, Red Bull, Merck, Bayer, The European Union, FWF Osterreichischer Fond zur Wissenschaftsforderung Bundesministerium für Wissenschaft und Forschung, and Jubiläumsfond der Österreichischen Nationalbank.

EW serves as DSMB member for Neurocrine, Acadia, GRIN and Encoded

FC has received speaker honoraria or consultancy fees from UCB Pharma, Eurofarma, Libbs, Torrent, Adium, Abbott, Prati Donaduzzi, Takeda, and Biocodex. He has also received Institutional grants from the Sao Paulo Research Foundation (FAPESP) and CNPq (Conselho Nacional de Desenvolvimento Científico e Tecnológico). He is the Editor-in-Chief of Epilepsia

JF receives salary support from the Epilepsy Foundation and from Epilepsy Study Consortium for consulting work and/or attending Scientific Advisory Boards for Acadia Pharmaceuticals, Acuta Capital Partners, Agrithera, Inc., Alterity Therapeutics Limited, Angelini Pharma S.p.A, Autifony Therapeutics Limited, Axonis Therapeutics, Baergic Bio, Beacon Biosignals, Inc., Biogen, Biohaven Pharmaceuticals, Bloom Science Inc., Bright Minds Biosciences, Inc., Camp4 Therapeutics Corporation, Cerebral Therapeutics, Cerecin Inc., Cerevel, Cognizance Biomarkers, Cowen and Company, LLC, Crossject, Eisai, Encoded Therapeutics, Engrail, Epalex, Epitel Inc, Equilibre BioPharmaceuticals, Genentech, Inc., Grin Therapeutics, IQVIA RDS Inc, iQure Pharma Inc., Janssen Pharmaceutica, Jazz Pharmaceuticals, Korro Bio Inc., Leal Therapeutics Inc, Lipocine, LivaNova, Longboard Pharmaceuticals, Marinus, Modulight.bio, Neumirna Therapeutics, Neurocrine, Neuronetics Inc., NeuroPace, Inc., NeuroPro Therapeutics, Neuroventis, Ono Pharmaceutical Co., Otsuka Pharmaceutical Development, Ovid Therapeutics Inc., Paladin Labs Inc., Praxis, PureTech LTY Inc., Rapport Therapeutics, Inc., Receptor Holdings Inc., Sage Therapeutics, Inc., SK Life Sciences, Stoke, Supernus, Takeda, Third Rock Ventures LLC, UCB Inc., Ventus Therapeutics, Vida Ventures Management, Xenon. J. French has also received research support from the Epilepsy Study Consortium (Funded by Eisai and UCB,) Epilepsy Study Consortium/Epilepsy Foundation (Funded by UCB), GW/FACES/One8Foundation and NINDS. She is on the editorial board of Lancet Neurology and Neurology Today. She is Chief Medical/Innovation Officer for the Epilepsy Foundation. She is the President and on the Board of Directors for the Epilepsy Study Consortium, Inc. She has received travel/meal reimbursement related to research, advisory meetings, or presentation of results at scientific meetings from the Epilepsy Study Consortium, the Epilepsy Foundation, Angelini Pharma S.p.A., Biohaven Pharmaceuticals, Cerebral Therapeutics, Cowen and Company, LLC, Longboard, Neurelis, Neurocrine, NeuroPace Inc., Praxis, Rapport, SK Life Science, Stoke, Takeda, Xenon.

JHC has acted as an investigator for studies with GW Pharma/Jazz Pharmaceuticals, Zogenix/UCB, Vitaflo, Stoke Therapeutics, Ultragenyx and Marinius. She has been a speaker and on advisory boards for Jazz Pharmaceuticals, , UCB, Biocodex and Nutricia; all remuneration has been paid to her department. She holds an endowed chair at UCL Great Ormond Street Institute of Child Health; she holds grants from NIHR, EPSRC, GOSH Charity, LifeARC and the National Institute of Health Research (NIHR) Biomedical Research Centre at Great Ormond Street Hospital. She is President of the ILAE 2021-2025.

JMW: National (South African) advisory board for Novartis and Sanofi. Associate Editor Epilepsia (honorarium for work covered).

MS served as Editor-in-Chief of Epilepsia. He has received compensation for speaking at CME programs from Medscape. He has consulted for Medtronic, Neurelis, and Johnson & Johnson. He has received research support from Medtronic; SK Life Science; Takeda; Xenon; Cerevel; UCB Pharma; Janssen; Equilibre; Epiwatch; Byteflies, Biohaven. He has received royalties from Oxford University Press and Cambridge University Press.

NS has served on scientific advisory boards for GW Pharma, BioMarin, Arvelle, Marinus and Takeda; has received speaker honoraria from Eisai, Biomarin, Livanova, Sanofi; has served as an investigator for Zogenix, Marinus, Biomarin, UCB, Roche. He was supported by #NEXTGENERATIONEU (NGEU) and funded by the Ministry of University and Research (MUR), National Recovery and Resilience Plan (NRRP), project MNESYS (PE0000006) – A Multiscale integrated approach to the study of the nervous system in health and disease (DN. 1553 11.10.2022). He was supported also by the Italian Ministry of Health with Current Research Funds.

SA is Deputy Editor for Epilepsia; has served as a consultant or received honoraria for lectures from Angelini Pharma, Biocodex, Eisai, Encoded, Jazz Pharmaceutics, Grintherapeutics, Neuraxpharm, Nutricia, Orion, Proveca, Stoke, Takeda, UCB Pharma, and Xenon; and has been an investigator for clinical trials for Eisai, Marinus, UCB Pharma, Proveca, and Takeda

SB serves as Editor-in-Chief of Epileptic Disorders. He received compensation for speaking at CME programs from Lundbeck, Eisai, UCB and GSK. He received research support from: Independent Research Fund Denmark; Innovation Fund Denmark; European Union: Eurostars Programme / EUREKA; European Union: Horizon Europe Framework Programme (HORIZON); Danish Agency for Higher Education and Science: International Network Programme.

SW received educational grants on behalf of his institution from UCB Pharma, Jazz Pharma, Paladin Labs, and served on the advisory board of Paladin Labs.

The remaining authors do not report conflicts of interest directly related to this paper.

**Ethical Publication Statement**

We conﬁrm that we have read the Journal’s position on issues involved in ethical publication and aﬃrm that this report is consistent with those guidelines.

**References**

1. Fisher RS, Cross JH, French JA, Higurashi N, Hirsch E, Jansen FE, Lagae L, Moshé SL, Peltola J, Roulet Perez E, Scheffer IE, Zuberi SM. Operational classification of seizure types by the International League Against Epilepsy: Position Paper of the ILAE Commission for Classification and Terminology. Epilepsia 2017; 58: 522-530.
   doi: 10.1111/epi.13670
2. Lüders H, Akamatsu N, Amina S, Baumgartner C, Benbadis S, Bermeo-Ovalle A, Bleasel A, Bozorgi A, Carreño M, Devereaux M, Fernandez-Baca Vaca G, Francione S, García Losarcos N, Hamer H, Holthausen H, Jamal Omidi S, Kalamangalam G, Kanner A, Knake S, Lacuey N, Lhatoo S, Lim SH, Mani J, Matsumoto R, Miller J, Noachtar S, Palmini A, Park J, Rosenow F, Shahid A, Schuele S, Steinhoff B, Szabo CÁ, Tandon N, Terada K, Van Emde Boas W, Widdess-Walsh P, Kahane P. Critique of the 2017 epileptic seizure and epilepsy classifications. Epilepsia 2019; 60: 1032-1039.
   doi: 10.1111/epi.14699.
3. Palmini A, Akamatsu N, Bast T, Bauer S, Baumgartner C, Benbadis S, Bermeo-Ovalle A, Beyenburg S, Bleasel A, Bozorgi A, Brázdil M, Carreño M, Delanty N, Devereaux M, Duncan JS, Fernandez-Baca Vaca G, García Losarcos N, Ghanma L, Gil-Nagel A, Hamer H, Holthausen H, Omidi SJ, Kahane P, Kalamangalam G, Kanner A, Knake S, Kovac S, Kraemer G, Kurlemann G, Lacuey N, Landazuri P, Hui Lim S, LoRusso G, Luders H, Mani J, Matsumoto R, Miller J, Noachtar S, O'Dwyer R, Park J, Reif PS, Rémi J, Rosenow F, Sakamoto A, Schubert-Bast S, Schuele S, Shahid A, Steinhoff JB, Strzelczyk A, Szabó CÁ, Tandon N, Terada K, Toledo M, van Emde Boas W, Walker M, Widdess-Walsh P. From theory to practice: Critical points in the 2017 ILAE classification of epileptic seizures and epilepsies. Epilepsia 2020; 61: 350-353.
   doi: 10.1111/epi.16426.
4. Rosenow F, Akamatsu N, Bast T, Bauer S, Baumgartner C, Benbadis S, Bermeo-Ovalle A, Beyenburg S, Bleasel A, Bozorgi A, Brázdil M, Carreño M, Delanty N, Devereaux M, Duncan J, Fernandez-Baca Vaca G, Francione S, García Losarcos N, Ghanma L, Gil-Nagel A, Hamer H, Holthausen H, Omidi SJ, Kahane P, Kalamangalam G, Kanner A, Knake S, Kovac S, Krakow K, Krämer G, Kurlemann G, Lacuey N, Landazuri P, Lim SH, Londoño LV, LoRusso G, Luders H, Mani J, Matsumoto R, Miller J, Noachtar S, O'Dwyer R, Palmini A, Park J, Reif PS, Remi J, Sakamoto AC, Schmitz B, Schubert-Bast S, Schuele S, Shahid A, Steinhoff B, Strzelczyk A, Szabo CA, Tandon N, Terada K, Toledo M, van Emde Boas W, Walker M, Widdess-Walsh P. Could the 2017 ILAE and the four-dimensional epilepsy classifications be merged to a new "Integrated Epilepsy Classification"? Seizure 2020; 78: 31-37.
   doi: 10.1016/j.seizure.2020.02.018
5. Beniczky S, Rubboli G, Aurlien H, Hirsch LJ, Trinka E, Schomer DL; SCORE consortium. The new ILAE seizure classification: 63 seizure types? Epilepsia 2017; 58: 1298-1300.
   doi: 10.1111/epi.13799.
6. Unterberger I, Trinka E, Kaplan PW, Walser G, Luef G, Bauer G. Generalized nonmotor (absence) seizures-What do absence, generalized, and nonmotor mean? Epilepsia 2018; 59: 523-529. doi: 10.1111/epi.13996
7. Commission on Classification and Terminology of the ILAE. Proposal for revised clinical and electroencephalographic classification of epileptic seizures. From the Commission on Classification and Terminology of the International League Against Epilepsy. Epilepsia 1981; 22: 489-501.
   doi: 10.1111/j.1528-1157.1981.tb06159.x.
8. Pressler RM, Cilio MR, Mizrahi EM, Moshé SL, Nunes ML, Plouin P, Vanhatalo S, Yozawitz E, de Vries LS, Puthenveettil Vinayan K, Triki CC, Wilmshurst JM, Yamamoto H, Zuberi SM. The ILAE classification of seizures and the epilepsies: Modification for seizures in the neonate. Position paper by the ILAE Task Force on Neonatal Seizures. Epilepsia 2021; 62: 615-628.
   doi: 10.1111/epi.16815.
9. Beghi E, Carpio A, Forsgren L, Hesdorffer DC, Malmgren K, Sander JW, Tomson T, Hauser WA. Recommendation for a definition of acute symptomatic seizure. Epilepsia 2010; 51: 671-5.
   doi: 10.1111/j.1528-1167.2009.02285.x.
10. Trinka E, Cock H, Hesdorffer D, Rossetti AO, Scheffer IE, Shinnar S, Shorvon S, Lowenstein DH. A definition and classification of status epilepticus--Report of the ILAE Task Force on Classification of Status Epilepticus. Epilepsia 2015; 56: 1515-23.
    doi: 10.1111/epi.13121.
11. Page MJ, McKenzie JE, Bossuyt PM, Boutron I, Hoffmann TC, Mulrow CD, Shamseer L, Tetzlaff JM, Akl EA, Brennan SE, Chou R, Glanville J, Grimshaw JM, Hróbjartsson A, Lalu MM, Li T, Loder EW, Mayo-Wilson E, McDonald S, McGuinness LA, Stewart LA, Thomas J, Tricco AC, Welch VA, Whiting P, Moher D. The PRISMA 2020 statement: an updated guideline for reporting systematic reviews. BMJ 2021; 372: n71.
    doi: 10.1136/bmj.n71.
12. Legnani M, Bertinat A, Decima R, Demicheli E, Higgie JR, Preve F, Braga P, Bogacz A, Scaramelli A. Applicability and contribution of the new ILAE 2017 classification of epileptic seizures and epilepsies. Epileptic Disord. 2019; 21: 549-554.
    doi: 10.1684/epd.2019.1108.
13. Kartheek T., Jayalakshmi S., Babu S.P., Patil A. Application of 1981 and 2017 ILAE Epilepsy classification of seizure types in an outpatient setting. Annals of Indian Academy of Neurology. Conference: 27th Annual Conference of Indian Academy of Neurology, IANCON 2019. Hyderabad India. 22(SUPPL 1) (pp S14), 2019.
14. Manchala D.A., Desai N., Udani V., Catherine S. Comparison of 1981, 1989 and 2017 International League against Epilepsy Classification (ILAE). Epilepsia. Conference: 33rd International Epilepsy Congress. Bangkok Thailand. 60(Supplement 2) (pp 33), 2019.
15. Casas Parera I, Gonzalez Roffo MA, Báez A, Quintans F, Castellanos Oropeza P, Sánchez Retamar MC. Characterization of seizures (ILAE 1981 and 2017 classifications) and their response to treatment in a cohort of patients with glial tumors: A prospective single center study. eNeurologicalSci. 2018; 14: 51-55.
    doi: 10.1016/j.ensci.2018.12.006.
16. Lewis-Smith D, Galer PD, Balagura G, Kearney H, Ganesan S, Cosico M, O'Brien M, Vaidiswaran P, Krause R, Ellis CA, Thomas RH, Robinson PN, Helbig I. Modeling seizures in the Human Phenotype Ontology according to contemporary ILAE concepts makes big phenotypic data tractable. Epilepsia 2021; 62: 1293-1305.
    doi: 10.1111/epi.16908.
17. Takahashi Y, Ota A, Tohyama J, Kirino T, Fujiwara Y, Ikeda C, Tanaka S, Takahashi J, Shinoki T, Shiraga H, Inoue T, Fujita H, Bonno M, Nagao M, Kaneko H. Different pharmacoresistance of focal epileptic spasms, generalized epileptic spasms, and generalized epileptic spasms combined with focal seizures. Epilepsia Open 2022; 7: 85-97.
    doi: 10.1002/epi4.12560.
18. Mielke H, Meissner S, Wagner K, Joos A, Schulze-Bonhage A. Which seizure elements do patients memorize? A comparison of history and seizure documentation. Epilepsia 2020; 61: 1365-1375.
    doi: 10.1111/epi.16550.
19. Sarmast ST, Abdullahi AM, Jahan N. Current Classification of Seizures and Epilepsies: Scope, Limitations and Recommendations for Future Action. Cureus 2020; 12: e10549.
    doi: 10.7759/cureus.10549.
20. Contreras Ramirez V, Patedakis Litvinov B, Gunawardane NA, Zhao CW, Yotter C, Quraishi IH, Blumenfeld H. Evaluating consciousness and awareness during focal seizures: responsiveness testing versus recall testing. Epileptic Disord. 2022; 24: 899-905.
    doi: 10.1684/epd.2022.1472.
21. Contreras Ramirez V, Vaddiparti A, Blumenfeld H. Testing awareness in focal seizures: Clinical practice and interpretation of current guidelines. Ann Clin Transl Neurol. 2022; 9: 762-765.
    doi: 10.1002/acn3.51552.
22. Howard R, Hirsch N, Kitchen N, Kullmann D, Walker M. Disorders of Consciousness, Intensive Care Neurology and Sleep. In Neurology: A Queen Square Textbook. Edited by: Clarke C, Howard R, Rossor M and Shorvon S. Blackwell Publishing 2009: 723-769.
    ISBN: 978-1-4051-3443-9.
23. Rogers G, O'Flynn N. NICE guideline: transient loss of consciousness (blackouts) in adults and young people. Br J Gen Pract. 2011; 61: 40-2.
    doi: 10.3399/bjgp11X548965.
24. <https://www.epilepsy.com/stories/impairment-consciousness-what-does-it-mean>
25. Rochat P. Five levels of self-awareness as they unfold early in life. Conscious Cogn. 2003; 12: 717-31.
    doi: 10.1016/s1053-8100(03)00081-3.
26. Beniczky S, Neufeld M, Diehl B, Dobesberger J, Trinka E, Mameniskiene R, Rheims S, Gil-Nagel A, Craiu D, Pressler R, Krysl D, Lebedinsky A, Tassi L, Rubboli G, Ryvlin P. Testing patients during seizures: A European consensus procedure developed by a joint taskforce of the ILAE - Commission on European Affairs and the European Epilepsy Monitoring Unit Association. Epilepsia 2016; 57: 1363-8.
    doi: 10.1111/epi.13472.
27. Steriade C, Sperling MR, DiVentura B, Lozano M, Shellhaas RA, Kessler SK, Dlugos D, French J. Proposal for an updated seizure classification framework in clinical trials. Epilepsia 2022; 63: 565-572.
    doi: 10.1111/epi.17120.
28. Turek G, Skjei K. Seizure semiology, localization, and the 2017 ILAE seizure classification. Epilepsy Behav. 2022; 126: 108455.
    doi: 10.1016/j.yebeh.2021.108455.
29. Alim-Marvasti A, Romagnoli G, Dahele K, Modarres H, Pérez-García F, Sparks R, Ourselin S, Clarkson MJ, Chowdhury F, Diehl B, Duncan JS. Probabilistic landscape of seizure semiology localizing values. Brain Commun. 2022; 4: fcac130.
    doi: 10.1093/braincomms/fcac130
30. Stefan H. The challenge epilepsy treatment – New epileptic drugs. Oxford: Blackwell science; 1998.
31. Chauvel P. Contributions of Jean Talairach and Jean Bancaud to epilepsy surgery. Epilepsy surgery. Philadelphia: Lippincott Williams & Wilkins; 2001.
32. McGonigal A, Bartolomei F, Chauvel P. On seizure semiology. Epilepsia 2021; 62: 2019-35.
33. Khoo A, Alim-Marvasti A, de Tisi J, Diehl B, Walker MC, Miserocchi A, McEvoy AW, Chowdhury FA, Duncan JS. Value of semiology in predicting epileptogenic zone and surgical outcome following frontal lobe epilepsy surgery. Seizure 2023; 106: 29-35.
    doi: 10.1016/j.seizure.2023.01.019
34. Specchio N, Wirrell EC, Scheffer IE, Nabbout R, Riney K, Samia P, Guerreiro M, Gwer S, Zuberi SM, Wilmshurst JM, Yozawitz E, Pressler R, Hirsch E, Wiebe S, Cross HJ, Perucca E, Moshé SL, Tinuper P, Auvin S. International League Against Epilepsy classification and definition of epilepsy syndromes with onset in childhood: Position paper by the ILAE Task Force on Nosology and Definitions. Epilepsia. 2022 Jun;63(6):1398-1442. doi: 10.1111/epi.17241. Epub 2022 May 3. PMID: 35503717.
35. Rubboli G, Tassinari CA. Negative myoclonus. An overview of its clinical features, pathophysiological mechanisms, and management. Neurophysiol Clin. 2006; 36: 337-43.
    doi: 10.1016/j.neucli.2006.12.001
36. Blume WT, Lüders HO, Mizrahi E, Tassinari C, van Emde Boas W, Engel J Jr. Glossary of descriptive terminology for ictal semiology: report of the ILAE task force on classification and terminology. Epilepsia 2001; 42: 1212-8.
    doi: 10.1046/j.1528-1157.2001.22001.x.
37. Beniczky S, Tatum WO, Blumenfeld H, Stefan H, Mani J, Maillard L, Fahoum F, Vinayan KP, Mayor LC, Vlachou M, Seeck M, Ryvlin P, Kahane P. Seizure semiology: ILAE glossary of terms and their significance. Epileptic Disord. 2022; 24: 447-495.
    doi: 10.1684/epd.2022.1430.
38. Meeren HK, Pijn JP, Van Luijtelaar EL, Coenen AM, Lopes da Silva FH. Cortical focus drives widespread corticothalamic networks during spontaneous absence seizures in rats. J Neurosci. 2002; 22: 1480-95.
    doi: 10.1523/JNEUROSCI.22-04-01480.2002.
39. Moeller F, LeVan P, Muhle H, Stephani U, Dubeau F, Siniatchkin M, Gotman J. Absence seizures: individual patterns revealed by EEG-fMRI. Epilepsia 2010; 51: 2000-10.
    doi: 10.1111/j.1528-1167.2010.02698.x.
40. Stefan and Trinka, Generalised Absence Seizures. Where do we stand today? Z.Epileptol. 2022; 35: 56–72 <https://doi.org/10.1007/s10309-022-00469-w>
41. Devinsky O, Elder C, Sivathamboo S, Scheffer IE, Koepp MJ. Idiopathic Generalized Epilepsy: Misunderstandings, Challenges, and Opportunities. Neurology 2024; 102: e208076.
    doi: 10.1212/WNL.0000000000208076.
42. McNally KA, Blumenfeld H. (2004). Focal network involvement in generalized seizures: New insights from ECT. Epilepsy and Behavior, 5: 3-12.
43. Blumenfeld H. (2005). Cellular and network mechanisms of spike-wave seizures. Epilepsia, 46 (Suppl. 9): 21–33.
44. Berg AT, Berkovic SF, Brodie MJ, Buchhalter J, Cross JH, van Emde Boas W, Engel J, French J, Glauser TA, Mathern GW, Moshé SL, Nordli D, Plouin P, Scheffer IE. Revised terminology and concepts for organization of seizures and epilepsies: report of the ILAE Commission on Classification and Terminology, 2005-2009. Epilepsia 2010; 51: 676-85.
    doi: 10.1111/j.1528-1167.2010.02522.x.
45. Seneviratne U, Woo JJ, Boston RC, Cook M, D'Souza W. Focal seizure symptoms in idiopathic generalized epilepsies. Neurology 2015; 85: 589-95.
    doi: 10.1212/WNL.0000000000001841.
46. Leutmezer F, Lurger S, Baumgartner C. Focal features in patients with idiopathic generalized epilepsy. Epilepsy Res. 2002; 50: 293-300.
47. Christie H, D'Souza W, Cook M, Seneviratne U. Can semiology differentiate between bilateral tonic-clonic seizures of focal-onset and generalized-onset? A systematic review. Epilepsy Behav. 2021; 116: 107769.
48. Vlachou M, Ryvlin P, Armand Larsen S, Beniczky S. Focal electro-clinical features in generalized tonic-clonic seizures: decision flowchart for a diagnostic challenge. Epilepsia 2024.
    doi: 10.1111/epi.17895.
49. Taylor E. We Agree, Don't We? The Delphi Method for Health Environments Research. HERD. 2020; 13: 11-23.
    doi: 10.1177/1937586719887709.
50. Lüders H, Acharya J, Baumgartner C, Benbadis S, Bleasel A, Burgess R, et al. Semiological seizure classification. Epilepsia 1998; 39: 1006-13.
51. Zuberi SM, Wirrell E, Yozawitz E, Wilmshurst JM, Specchio N, Riney K, Pressler R, Auvin S, Samia P, Hirsch E, Galicchio S, Triki C, Snead OC, Wiebe S, Cross JH, Tinuper P, Scheffer IE, Perucca E, Moshé SL, Nabbout R. ILAE classification and definition of epilepsy syndromes with onset in neonates and infants: Position statement by the ILAE Task Force on Nosology and Definitions. Epilepsia 2022; 63: 1349-1397.
    doi: 10.1111/epi.17239
52. Salas-Puig X, Iniesta M, Abraira L, Puig J. Accidental injuries in patients with generalized tonic-clonic seizures. A multicenter, observational, cross-sectional study (QUIN-GTC study). Epilepsy Behav. 2019; 92: 135–9.
53. Ryvlin P, Nashef L, Lhatoo SD, et al. Incidence and mech-anisms of cardiorespiratory arrests in epilepsy monitoring units (MORTEMUS): a retrospective study. Lancet Neurol. 2013; 12: 966–77.
54. Sveinsson O, Andersson T, Mattsson P, Carlsson S, Tomson T. Clinical risk factors in SUDEP: a nationwide population-based case-control study. Neurology 2020; 94: e419–29.
55. Harden C, Tomson T, Gloss D, et al. Practice Guideline Summary: sudden unexpected death in epilepsy incidence rates and risk fac-tors: report of the guideline development, dissemination, and imple-mentation subcommittee of the American Academy of Neurology and the American Epilepsy Society. Epilepsy Curr. 2017; 17: 180–7.
56. Mayville C, Fakhoury T, Abou-Khalil B. Absence seizures with evolution into generalized tonic-clonic activity: clinical and EEG features. Epilepsia 2000; 41: 391–394.
57. Beniczky S, Rubboli G, Covanis A, Sperling MR. Absence-to-bilateral-tonic-clonic seizure: A generalized seizure type. Neurology 2020; 95: e2009-e2015.
    doi: 10.1212/WNL.0000000000010470.
58. Fisher RS, Helen Cross J, D'Souza C, French JA, Haut S, Higurashi N, Hirsch E, Jansen FE, Lagae L, Moshe SL, Korey SR, Purpura DP, Peltola J, Roulet Perez E, Scheffer IE, Schulze-Bonhage A, Somerville E, Sperling M, Yacubian EM, Zuberi SM. Response to the numbering of seizure types. Epilepsia 2017; 58: 1300-1301.
    doi: 10.1111/epi.13800.
59. Fisher RS, Cross JH, D'Souza C, French JA, Haut SR, Higurashi N, Hirsch E, Jansen FE, Lagae L, Moshé SL, Peltola J, Roulet Perez E, Scheffer IE, Schulze-Bonhage A, Somerville E, Sperling M, Yacubian EM, Zuberi SM. Instruction manual for the ILAE 2017 operational classification of seizure types. Epilepsia 2017; 58: 531-542.
    doi: 10.1111/epi.13671.
60. Loddenkemper T, Kellinghaus C, Wyllie E, Najm IM, Gupta A, Rosenow F, Lüders HO. A proposal for a five-dimensional patient-oriented epilepsy classification. Epileptic Disord. 2005; 7: 308-16.

1. **Changes tracked in the definition of the generalized seizures**

**Definitions of generalized epileptic seizure types**

**Typical absence seizure (TA; 3.1.1)** is a generalized epileptic seizure characterized by sudden onset, interruption of ongoing activities, a blank stare (loss of facial expression), and possibly a brief upward deviation of the eyes. Usually the patient is unresponsive; in most cases, awareness is impaired too. However, occasionally, after the seizure, patients may recall the ictal events (for example test words given during the seizure). Oral and/or manual automatisms occur in 86% of patients and eye involvement with blinking, eye opening, or subtle eyelid or perioral myoclonus in 76.5% of patients. There is immediate return to normal activity, although children may be momentarily confused as they reorient themselves. Duration is a few seconds to half a minute (median 7 s; range: 2-26 s*) but rarely they may last >30 s. Ictal EEG is characterized by regular, bilateral-synchronous (“generalized”) spike-waves. In the first seconds of seizure onset, the frequency of the spike-waves is around 3 Hz; range: 2.5–4 Hz in Childhood Absence Epilepsy (CAE), 3-5.5 Hz in Juvenile Absence Epilepsy (JAE). Disorganized discharges, defined by brief (<1 s) or transient interruptions in the ictal rhythm, or waveforms of different frequency or morphology are significantly less common in CAE than in JAE. The seizures are typically provoked by hyperventilation in most untreated patients with CAE. They may be provoked by intermittent photic stimulation too. In CAE, seizures typically occur multiple times per day but are often under-recognized. In JAE, typical absence seizures occur less than daily in the untreated state.

**Atypical absence seizure (AA; 3.1.2)** is a generalized seizure type characterized by episodes of impaired consciousness (awareness and/or responsiveness). Changes in tone (when present) are more pronounced than in typical absence seizures (for example head-drop as opposed to mild head retropulsion), and the onset and/or cessation is gradual (not abrupt). Duration is usually longer than of typical absence seizures, but with considerable overlap (median: 15 s; range: 2-10 s*). Ictal EEG shows irregular, bilateral synchronous and asynchronous/asymmetric spike-waves, with frequency lower than in typical absence (<2.5 Hz) and the ictal activity may be fragmented or include fast activity. Atypical absence seizures may occur in Lennox-Gastaut syndrome.

**Myoclonic absence seizure (MA; 3.1.3)** are absence seizures with abrupt onset and offset, associated with rhythmic 3-Hz jerks of the upper limbs, superimposed on tonic abduction of the arms during the seizure (giving a ratcheting appearance). The patient, if standing, typically bends forward during the seizure, but falling is uncommon. The myoclonic jerks are typically bilateral and symmetric but can be unilateral or asymmetric. Perioral myoclonia and rhythmic jerks of the head and legs may also occur. Impairment of consciousness varies from complete loss of awareness and responsiveness to retained awareness and responsiveness. Occasionally, autonomic manifestations, such as a change in breathing or urinary incontinence, or complex gestural automatisms, may be seen. Duration is typically 7–12 s, but occasionally longer (up to 60 s) and may occur multiple times per day. Ictal EEG shows regular 3Hz, bilateral-synchronous (“generalized”) spike-waves, time-locked with the myoclonic jerks. Myoclonic absence seizures occur in a variety of genetic conditions; this seizure type is mandatory for the diagnosis of epilepsy with myoclonic absence syndrome. Polygraphic recordings of EMG with EEG is recommended for ictal recordings.

**Eyelid myoclonia with / without absence (EMA; 3.1.4)**. Eyelid myoclonia, consists of brief, repetitive, and often rhythmic 3–6-Hz myoclonic jerks of the eyelids, often with simultaneous upward deviation of the eyeballs and extension of the head. Eyelid myoclonia can be associated with absences, but can occur without a corresponding absence. They are typically induced by involuntary or voluntary slow eye closure or exposure to bright sunlight. These seizures are very brief (median duration: 1.5 s; range: 0.5-8 s*) and occur multiple times each day, even many times per hour. Ictal EEG shows bilateral synchronous (“generalized”) fast spike activity or 3–6 Hz polyspike-and-wave discharges, typically elicited by eye closure and intermittent photic stimulation, especially in untreated patients. This seizure type is mandatory for the diagnosis of epilepsy with eyelid myoclonia (formerly called Jeavons syndrome).

**Generalized tonic-clonic seizure (GTC; 3.2)** consists of a tonic phase, with sustained muscle activity, followed by a clonic phase with progressive slowing of the clonic jerks, due to the gradual increase in the duration of the silent-periods interrupting the muscle activation, which eventually terminate the seizure. These motor phenomena are bilateral, but not always symmetric, and focal features (such as forced head version) may be observed in generalized tonic-clonic seizures. Typically, there is loss of consciousness during the seizure and in the postictal period. GTC seizures may be preceded by sporadic or irregular myoclonic jerks (Generalized myoclonic-tonic-clonic seizure; 3.2.1) often seen in patients with Juvenile Myoclonic Epilepsy, or by an absence seizure (absence-to-bilateral-tonic-clonic seizure; 3.2.2). Median duration of GTCS is 80 s (range: 57 – 102 s*). The ictal EEG is often obscured by movement artifact. Bilateral-synchronous (“generalized”) fast rhythmic spikes may be seen in the tonic stage, which is followed by bursts of spikes and slow-waves, synchronous with clonic jerks, during the clonic phase. A postictal period of generalized EEG suppression (PGES) or irregular, diffuse slow activity follows a GTC seizure. In idiopathic generalized epilepsies, GTC seizures often occur on awakening or with sleep deprivation. In focal conditions, the seizure is classified as Focal-to-bilateral tonic-clonic seizure (FBTC; 1.3). When the origin is unknown, the seizure is classified as Bilateral tonic-clonic seizure, unknown whether focal or generalized (BTC; 2.3). Tonic-clonic seizures have the highest associated morbidity and mortality, and represent the major risk factor of Sudden Unexpected Death in Epilepsy (SUDEP).

**Generalized myoclonic seizure (GM; 3.3.1).** Myoclonic jerks (a.k.a. myoclonus, plural: myoclonia) were defined as sudden, brief (lightening-like; <100-msec) involuntary, single or multiple irregular/arrhythmic contractions of muscles or muscle groups. When measured using surface electromyogram (EMG), their median duration was 80 ms (range: 30-140 ms*). Generalized myoclonic seizures are bilateral but can predominate on one side of the body, frequently involving the upper extremities. They can also involve the lower limbs and cause falls. Generalized myoclonic seizures can be reflex, triggered by photic stimulation or praxis. The typical ictal EEG correlate is bilateral-synchronous (“generalized”) polyspike-and-wave discharges (or spike-and-wave discharge), time-locked to the myoclonus. Generalized myoclonic seizures are mandatory for the diagnosis of Juvenile Myoclonic Epilepsy, and they may occur in other generalized epilepsies too. Note that unilateral myoclonic jerks can occur in focal seizures, in which case they are classified as focal seizures, and myoclonus added as a descriptor of seizure semiology.

**Generalized clonic seizure (GC; 3.3.2)** consists of myoclonic jerks that are regular and repetitive, at a relatively low frequency (typically 0.2-5 Hz) and involve the same muscle groups. Generalized clonic seizures are bilateral, but not always synchronous and symmetric. Duration is 4 s (range: 1 – 24 s*). Ictal EEG shows generalized spike-and-wave or polyspike-and-wave discharges, time-locked to the clonic jerks. Note that unilateral or asymmetric clonic phenomena can occur in focal seizures, in which case they are classified as focal seizures, and clonic is added as a descriptor of seizure semiology. Generalized clonic seizures should be distinguished from myoclonic absence seizures, which exhibit distinct movement patterns (see 2.1.3).

**Generalized negative myoclonic seizure (GNM; 3.3.3)** is defined as a brief interruption of muscle tone (<500 ms), causing a sudden, brief lapse in movement that may grossly appear like a myoclonic jerk. Generalized negative myoclonic seizures are bilateral, but not always synchronous and symmetric. To document negative myoclonus, it is often necessary to instruct the patient to perform a voluntary muscle activation, such as lifting the arms. The EEG correlate is a spike-wave or a low-amplitude sharp-transient. The onset of the EMG silent-period is related to a negative component of the spike on the EEG, occurring before the slow wave. In progressive myoclonic epilepsies, a cortical involvement has been demonstrated in cortical reflex negative myoclonus. Unilateral or asymmetric negative myoclonus can occur in focal seizures, in which case they are classified as focal seizures, and negative myoclonus is added as a descriptor of seizure semiology. Subcortical negative myoclonus may occur in metabolic encephalopathies.

**Generalized epileptic spasms (GES; 3.3.4)** consist of brief contractions of axial (predominantly truncal and proximal) muscles, each typically lasting ≤2 s (median: 1 s; range: 0.4-2 s*), causing abduction and extension of both arms, hip flexion and nodding. Subtle forms of spasms, with minimal / discrete manifestations may occur, including head nodding, grimacing, smiling, or chin movement. Epileptic spasms usually occur in clusters, often upon awakening, with increasing prominence of the motor features through the cluster, often over a period of minutes (although clusters may last 30 min or longer). The ictal EEG correlate is characterized by a high amplitude, generalized, sharp or slow wave that is followed by low amplitude, fast activity or a brief, diffuse electrodecrement. Surface EMG helps to distinguish epileptic spasms from myoclonic seizures and tonic seizures. The EMG of an epileptic spasm has a typical diamond-shape (gradual increase and gradual decrease in amplitude). Epileptic spasms are mandatory for the diagnosis of Infantile Epileptic Spasm Syndrome. Epileptic spasms may occur in focal / structural epilepsies, in which case they may appear unilateral or asymmetric. However, the bilateral symmetric semiology does not rule out the focal origin, and a complex multimodal investigation, including video-polygraphic recordings, neuroimaging, laboratory and genetic tests are needed to correctly classify epileptic spasm. When epileptic spasm occurs in a focal condition, it is classified as focal seizure, and epileptic spasm is added as a descriptor of seizure semiology. When the origin is uncertain, epileptic spasm is classified as unknown whether focal or generalized.

**Generalized tonic seizure (GT; 3.3.5)** is defined as sustained muscular contraction resulting in stiffness or tense posture, that usually causes an extension, but it may also affect the flexor muscles. The median duration of generalized tonic seizures is 8 s (range: 3 – 51 s*). Generalized tonic seizures are bilateral, but not necessarily symmetrical. They may be subtle, with slow upward eye rolling or deviation, at times with facial grimace or flexor movements of the head and/or trunk, or more clinically obvious, with a brief cry, apnea, abduction, and elevation of the limbs with a vibratory component and bilateral fist clenching. If occurring while the patient is standing, they may forcefully throw the patient off balance, leading to a fall with the patient often sustaining an injury. Tonic seizures can be precipitated by startle. During sleep, generalized tonic seizures may be very subtle and not recognized by the family members, and therefore need polygraphic recording of sleep to identify and quantify seizure frequency. Generalized tonic seizures may be preceded or followed by spasms (colloquially termed “tonic spasms”), a myoclonic jerk (“myoclonic-tonic seizure”), or a hyperkinetic seizure followed by a spasm (“hypermotor-tonic-spasms”). The ictal EEG pattern of tonic seizures consists of a burst of bilateral 10 Hz or higher frequency fast activity with a recruiting rhythm, an initial diffuse decrement followed by gradual increase in amplitude. Generalized tonic seizures are mandatory for diagnosis of Lennox-Gastaut syndrome. Focal tonic ictal phenomena may occur in focal seizures, in which case they are classified as focal seizures, and tonic is added as a descriptor of the seizure semiology.

**Generalized atonic seizure (GA; 3.3.6)** is defined as a sudden loss or decrease in muscle tone, without apparent preceding myoclonic or tonic event, involving the head, trunk, jaw, and limbs. Due to the loss of postural tone, atonic seizures frequently cause falls and injury. The median duration is 1 s (range: 0.5 – 13 s*). Polygraphic recordings including surface EMG of the antagonist muscles are useful to document this seizure type. Atonic seizures are often observed in Lennox-Gastaut syndrome. They may occur in focal epilepsies too, in which case they are classified as focal seizures and atonic is added as a descriptor of the seizure semiology.

**Generalized myoclonic-atonic seizure (GMA; 3.3.7)** is characterized by a brief myoclonic jerk affecting the proximal muscles, often associated with a slight vocalization, followed by a very brief atonic component, which may be subtle, with a head nod, or more prominent, with an abrupt fall. Median duration is 1.25 s (range: 0.7 - 1.5 s*). Ictal EEG shows bilateral-synchronous (“generalized”) polyspike or spike discharges with the myoclonus, followed by a high-voltage slow-wave accompanying the atonic component. Polygraphic recordings of EMG with EEG is recommended for ictal recordings. Myoclonic–atonic seizures are mandatory for diagnosis of Epilepsy with Myoclonic-Atonic Seizures (Doose syndrome).

*The definitions are adapted from the following papers:*

Fisher RS, Cross JH, D'Souza C, French JA, Haut SR, Higurashi N, Hirsch E, Jansen FE, Lagae L, Moshé SL, Peltola J, Roulet Perez E, Scheffer IE, Schulze-Bonhage A, Somerville E, Sperling M, Yacubian EM, Zuberi SM. Instruction manual for the ILAE 2017 operational classification of seizure types. Epilepsia. 2017;58:531-542. doi: 10.1111/epi.13671.

Zuberi SM, Wirrell E, Yozawitz E, Wilmshurst JM, Specchio N, Riney K, Pressler R, Auvin S, Samia P, Hirsch E, Galicchio S, Triki C, Snead OC, Wiebe S, Cross JH, Tinuper P, Scheffer IE, Perucca E, Moshé SL, Nabbout R. ILAE classification and definition of epilepsy syndromes with onset in neonates and infants: Position statement by the ILAE Task Force on Nosology and Definitions. Epilepsia. 2022;63:1349-1397. doi: 10.1111/epi.17239.

Specchio N, Wirrell EC, Scheffer IE, Nabbout R, Riney K, Samia P, Guerreiro M, Gwer S, Zuberi SM, Wilmshurst JM, Yozawitz E, Pressler R, Hirsch E, Wiebe S, Cross HJ, Perucca E, Moshé SL, Tinuper P, Auvin S. International League Against Epilepsy classification and definition of epilepsy syndromes with onset in childhood: Position paper by the ILAE Task Force on Nosology and Definitions. Epilepsia. 2022;63:1398-1442. doi: 10.1111/epi.17241.

Hirsch E, French J, Scheffer IE, Bogacz A, Alsaadi T, Sperling MR, Abdulla F, Zuberi SM, Trinka E, Specchio N, Somerville E, Samia P, Riney K, Nabbout R, Jain S, Wilmshurst JM, Auvin S, Wiebe S, Perucca E, Moshé SL, Tinuper P, Wirrell EC. ILAE definition of the Idiopathic Generalized Epilepsy Syndromes: Position statement by the ILAE Task Force on Nosology and Definitions. Epilepsia. 2022;63:1475-1499. doi: 10.1111/epi.17236.

Beniczky S, Tatum WO, Blumenfeld H, Stefan H, Mani J, Maillard L, Fahoum F, Vinayan KP, Mayor LC, Vlachou M, Seeck M, Ryvlin P, Kahane P. Seizure semiology: ILAE glossary of terms and their significance. Epileptic Disord. 2022;24:447-495. doi: 10.1684/epd.2022.1430.

Meritam Larsen P, Wüstenhagen S, Terney D, Gardella E, Aurlien H, Beniczky S. Duration of epileptic seizure types: A data-driven approach. Epilepsia. 2023;64:469-478. doi: 10.1111/epi.17492.

1. **The list of public comments, grouped according to the topic they addressed**
2. **Overall assessment**

**SUPPORTIVE / APPROVING: 25 comments + Epilepsia editors & reviewers + ILAE-Executive Committee**

**CRITICAL / DISAPPROVING: 5 comments**

**FOR / APPROVING**

#2: I have read your precious proposal with great interest and wish to congratulate you to this outstanding achievement.

#3: It looks very clear and sensible. I think it is an improvement.

#4: This updated approach not only reflects a better understanding of the condition but also promises to alleviate many of the ongoing debates among neurologists and epileptologists. By establishing clearer definitions, we can improve both daily practice and research, leading to more targeted interventions and better outcomes for our young patients. Such advancements in our field are crucial. They not only refine our medical practices but also foster a more nuanced understanding among all healthcare providers involved in the care of children with epilepsy. This collective progress is essential for advancing our field and improving the lives of those affected by these challenging conditions. As we continue to build on these foundations, I remain hopeful for the future of epilepsy treatment and research, particularly in regions like ours where the need is profound and the impact of such advancements can be truly life-changing.

#6: As Chair of the 2017 Seizure Classification taskforce, I am gratified that the Updated Classification revisions are limited and based on literature, 7 years of experience in the field and expert consensus. The structure and majority of terminology from the 2017 classification is retained. The revision supports the decision to classify by the first manifestation, rather than the most prominent, because the first identifies the part of brain or network pointing to the most likely site of an important lesion. The revision also continues to allow use - when available - of ancillary information and not exclusively on semiology.

#8: Excellent update.

#9: Thank you to the working group for the proposed changes, most of which I believe represent improvements. In addition, the authors have to be commended for providing simultaneous translations, which I think is of major importance for promoting wide implementation.

#11: I congratulate the working group on this updated classification.

#12: Congratulations in achieving a feat and making such a detailed classification with newer data.

#14: The revised classification is comprehensive and easier to translate to the local language. Well done to the committee.

#16: Congratulations to working team. It is a clear update, without ambiguity.

#18: I want to congratulate you to your masterpiece of work.

#22: First and foremost, thank you for the tremendous effort and dedication you’ve put into updating the seizure classification system. This work is clearly the result of significant reflection and collaboration, and it represents an important step forward in making the classification more adaptable and accessible across clinical settings globally.

#25: The panel is congratulated with the proposed simplification, in particular by avoiding confusion about awareness and consciousness (and its translation).

#26: I would acknowledge the Commission for the huge effort. The consensus gave an acceptable frame for the nosological rules behind the Classification.

#29: We would like to express our gratitude for the outstanding updated proposal for the classification of epileptic seizures in 2024. This proposal reflects the relentless efforts of a dedicated team and is underpinned by a robust research design.

#32: Updated Classification of Epileptic Seizures- is an excellent initiative made by ILAE, most of the shortcomings and lack of explanations of 2017 classifications are carefully deleted with more elaborate and descriptive classification.

#34: Thanks.

#35: I appreciate the effort of the working group to update and simplify the classification of epileptic seizures.

#36: I really appreciate the detailed update on epileptic seizure and epilepsy syndromes. I think the changes are very useful.

#37: The current proposal is better than the previous version.

#38: I genuinely appreciate the effort of the working group to update and simplify the classification of epileptic seizures. I agree with most of the proposed changes, in particular to re-adopt the term “consciousness” that is more widely used in medical language – not only in clinical neurology – respect to “awareness”, allowing a more homogeneous language particularly in the context of differential diagnosis (e.g. with syncope and other transitory “loss of consciousness” events).

#39: On behalf of the Department of Paediatric Neurology of the University of Antioquia, we would like to express our appreciation for the recent update of the epilepsy classification, which undoubtedly represents an important advance in the management and diagnosis of this complex disease.

#40: I have gone through the draft of the ILAE position paper. It is extensively researched, and clearly documented. No suggestions / revisions from my end. I greatly appreciate the tremendous efforts you and your team have put into developing this important position paper.

#41: I also approve.

#42: I am very pleased to see a more rigorous methodology being applied to this and I, of course, approve.

Formal review in Epilepsia

*Editor-in-chief:* the paper should be considered for publication only with relatively minor revisions.

*Reviewer-1*: Interesting suggested modifications of terminology promoted by the ILAE that preserves the fundamental structure of that previous system. The proposed classification notably provides some improvements in clarity and applicability to clinical practice.

The authors are commended for this thoughtful and extensive review of seizure classification.

*Reviewer-2:* This is an updated version of the 2017 ILAE classification of epileptic seizures. I must start by congratulating the ILAE for this initiative and the authors for demonstrating an honest willingness to incorporate interactions with the epileptological community and thus provide ever more useful classifications of epileptic seizures. I also believe it should be commended the way the members of this ‘Task Force’ were selected, including specialists practicing in diverse cultures and socio-economical scenarios – which encouraged changes that make seizure types easier to conceptualize, describe and communicate.

The overall framework is very good and the ‘return’ of the term consciousness, with crucial understanding that it encompasses either awareness, responsiveness or both is very welcome.

It is also a strength of this renewed initiative the encouragement to describe seizure evolution, as it provides an immediate ‘topographical map’ of epileptogenic networks that is very useful for teaching, EEG interpretation and, above all, to raise the possibility of surgical considerations.

Overall, this paper represents an important addition to epileptology that must be commended by all.

**CRITICAL / DISAPPROVING**

#13: Attempting to classify seizures better is a valiant effort and is to be applauded. Kudos to the team for putting in the effort. However, I would advocate strongly for keeping the same names as before. Changing names every few years leads to an incredibly fragmented literature and difficulty in communicating with non-epilepsy specialists who do not understand why we keep changing things (neither do I). We have community physicians who speak of petit mal, complex partial seizures, and university-based physicians who speak of dyscognitive seizures, and focal impaired awareness seizures depending on when each person trained. This terminology heterogeneity affects research in trying to put together different terminologies over the years. This also detracts new trainees who find the field very confusing and move on to other areas.

FAS -> FPC and FIAS -> FIC may seem like progress, but is a backwards step in my opinion. I am sure the team had similar discussions between this and reached this consensus, similar to all the other times in the past when the names were changed, and similar to the future times when this will change. If the price to pay for progress is added confusion, perhaps progress is not worth the price. Hope this highly qualified team does not take this as unwarranted criticism, and adds this opinion to the mix.

#20: Finally, a classification system aims to standardize terminology for clinical communication and research phenotyping. For these objectives to be met, consistency and stability over time are crucial. Therefore, I value the comprehensive expertise of ILAE members in developing a classification system with lasting and widespread adoption in epileptology and neurology. Given that the previous classification was introduced only seven years ago, frequent reclassifications and terminological changes might disrupt the field and hinder the acceptance of the system among non-specialist neurologists.

#30: I congratulate the authors on this very well written article and on their care for embracing other languages and cultures. Please, interpret my following suggestion as a respectful comment. I believe the text is excellent and most of the changes are appropriate. However, I strongly suggest the authors and/or ILAE commission reconsider the publication of this update. The fact that the 2017 classification manuscript ended with a statement suggesting revisions is not a good reason to do so. I believe the current working group could and should conclude that modifications are not necessary at this time. Actually, I believe changes, that in practical consequences are a new classification, would bring more harm than good. As the authors say, the primary objective is the establishment of a common language for clinical practice and research. It is clear that frequent reclassifications work against this main goal and not in its favor. Another harm of reclassifications is the time doctors, students, residents and researchers waste with the classification itself instead of really studying epilepsy. Classifications are inevitably overvalued, leading students to concentrate on the words instead of the actual phenomena. After this article gets published, I will have to say to all the residents that come with an interesting case to discuss: “now we don’t say unaware, we say impaired consciousness”. It may seem wispy, but we have limited time for patient care and teaching. I would have to spend a reasonable amount of time teaching vocabulary instead of using the time to deal with actual questions and ideas. I believe classifications should only be changed when there is a very strong reason, for example: new discoveries regarding mechanisms or causes (as the pathological classifications of dementia, methylation-based classification of tumors). I believe the critics and variations in conceptions, not strongly based in new facts, should not be enough to justify the cons of a new classification.

For example, the neuroscience and epilepsy community have been discussing consciousness for a very long time, including its appropriateness as a seizure descriptor. I believe changes in the classification should not reflect what the current workgroup thinks, even if it is right, to avoid constant changes based on changes in ILAE’s commissions and the opinions of the contemporary active members. I believe ILAE should change the classification only if relevant real changes in the field occur, usually when new mechanisms are discovered. So, I agree with the authors on the consciousness issue, but the harms of a new classification (which it is for its practical consequences) outweigh the gains.

I see there are some comments against frequent changes in classification, but I believe this real-world opinion is still underrepresented here, besides being the most important one. Most of the neurologists and epileptologists I talk to think there are too many reclassifications. However, for some reason, they do not comment here.

If the reclassification remains, I have minor suggestions.

#33: Despite a great deal of effort on the part of this working group, it is my view that the proposed changes are a huge step backward in the classification of seizures. the document reminds me of when I was an epilepsy fellow, back in the 1980's, where the field was full of complicated jargon that was not easily digestible. Instead of making seizure classification easy and simple to understand this new proposal makes it overly complicated, especially for non-epilepsy specialist care providers, where they now have to recognize up to 20 seizure types. That is ridiculous.

Relying mostly on seizure manifestations harkens back to clinical care nearly 100 years ago. It wasn't workable then so why should it work now? We have learned much since then but it has been through the integration of semiology with other diagnostic information, such as imaging, genetics, etc.

It is important that any classification system has merit in either diagnosis and/or treatment, which the proposed system seems to have limited value. Overall, I see this proposal as a major step backward, and I for one feel compelled to question the value of both using it and teaching it to students, residents, and trainees. If others feel the same way it is important for voices to speak up as we are a community that should speak with one voice.

#43

I appreciate that a lot of hard thinking and discussion will have been done by the commission members to produce this document and I am grateful for this work which I know will have been done at a sacrifice to their personal and family time. I also am certain that this proposal has been developed with the best of intentions and that every member of the commission deeply want to improve the quality of life for people with epilepsy.

Respectively however, these proposals do not represent minor adjustments to the 2017 classification – these are major changes and are coming at a time when the 2017 classification are still being embedded into clinical practice and data systems. I would suggest that no one would look at these two figures below and say the differences are minor.


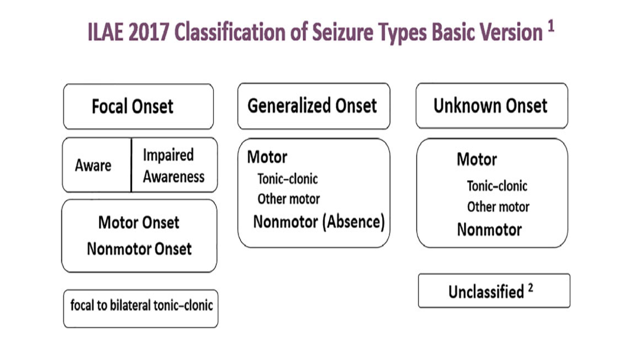


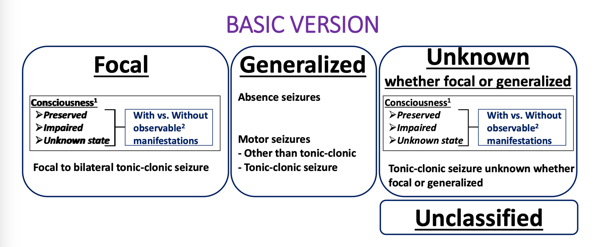


Although I have commented below on each of the specific proposed changes my MAJOR concern is that this is **too soon, too fast and represents too much change**. In addition, the rationale for making these changes are not particularly science based and not that relevant to the majority of people who use these terms. There is significant risk of reputational damage for the ILAE as the majority of people (patients, lay organisations, allied health, medical students, residents, doctors who are not neurologists etc) who are only starting to come to terms with the 2017 classification will simply see this as a reason not to bother with learning any ILAE classification if it is only going to change every 7 years.

When I first became involved in epilepsy and was getting my head around complex partial seizures one of my older neurology mentors, who had been on earlier ILAE classification taskforces, told me not to get too attached to names and concepts as the ILAE is always changing them – he had become very cynical about the process. At the time I thought that was very sad. Now I realise that many of my present colleagues (particularly those of my vintage and older) are also very jaded about the number of times new proposals come out and names are proposed. At a recent meeting of the child neurologists of New Zealand and Australia when a new proposal was mentioned there was a very large sigh - the sentiments heard were “not again”, “ you have got be joking”, mixed with some laughter from those that were never on board with the 2017 classification and still using older terminology.

My experience of being involved in a peripheral capacity of the ILAE classification process (member of the epilepsydiagnosis.org taskforce and chair of the SNOMED taskforce) has given me insight into the strongly held differences in opinion of neurologists based on where they trained and what their epilepsy focus is – in other words how they personally used the concepts and terms. Each neurologist has good rationales for why their way is the “right” or “best” way with many of them not being particularly open minded to other ways or approaches. My reflection on this is that either some people are “wrong” and others are “right” or, more likely ☺, that there are multiple ways to view things depending on your perspective and one neurologists way may be “right” for them even if not “right” for someone else.

When the 2017 classification came out we were led to believe by the ILAE that this was a line in the sand – that we would move forward with these names and concepts. I forged ahead, evangelistically spreading the word and used the new terms and concepts with in medical school teaching, guidelines, publications and coding updates. Teaching and explaining the 2017 classification is logarithmically easier than the previous 1985 version – people get it – the terminology is easy to understand.

From my perspective most of what is written in this 2024 proposal is good but it is just another way to skin the cat – if this had been the way it came out in 2017 that would have been fine but it didn’t…… and there is not any real justification for making most of these major changes other than that some people disagree with the 2017 terminology – it seems more political than science based. A different group of “experts” with different opinions on how they would like to see it – this time backed by a Delphi process. There IS NO classification system that will ever be met with unified agreement and so changing the classification purely based on a different group of people with power (and I say that as fondly as possible) isn’t helpful for the ILAE or people with epilepsy. In addition, the bottom line is the majority of these proposals are not proposing changes to concepts – they are simply suggesting changes to terminology used for concepts. I agree words are important, but consistency has to be weighed up against the benefit of just changing a name.

I am presently the Chair of the SNOMED taskforce. This experience has really opened my eye to ontologies, concepts and terminology as well as the importance of everyone being on the same page with regard to Big Data coding. It is almost the end of 2024 and we have only just got the 2017 (7 years on) concepts and terminology in SNOMED –that process has taken 2 years of work and it will take more time (years) before those updated names will find their way into the end user interfaces (EPIC etc.) HPO only updated their concepts and terminology a few years ago. INSERM and Orphanet are still updating their terminology. ICD updates are years from becoming used in many countries. My point is if you change terminology at rates that are faster than the world (people and systems) can keep up – you will risk having no one being on the same page as different systems and people are at different points along the journey. So when you try to use the data generated it will not be accurate or helpful or harmonised. This ultimately is not be good for people with epilepsy as this data is used for resource allocation, funding and research.

The ILAE needs to look at the big picture which is crucially more important than if the absolute “correct” terminology for a concept is consciousness or awareness as based on EMU studies.

Another major concern relates to the Ontology of what you are proposing – I don’t think it is sound and won’t get into the electronic medical records because the current international medical terminology systems (SNOMED and HPO) will not allow some of the terms to be put in. The 2017 classification had the same problem and we have had to modify it to get it into SNOMED and HPO. It would be better if we just had an ILAE classification scheme that had a better ontology so that the terms could go directly into electronic records.

1. **Seizure classes**

**The framework**

#25: Why not allow or even encourage TONIC-CLONIC-SEIZURE as an identical item in all the three boxes of Figure 2?

The seizure classification for the most common presentations should be used by patients, their relatives and caregivers, general neurologists, paediatricians, emergency physicians, stroke unit nurses, ..., and even by epileptologists. A presentation with a "tonic-clonic seizure from sleep with Todd's paresis " is common. "Focal to bilateral" is often assumed rather than observed. Certainly [1], people differ in their attitude to unanswerable questions [2]. If one forces observers to tick a box, a shockingly high percentage will report inobservable signs [3], and this may result in overdiagnosis. Further, allowing (and encouraging) tonic-clonic seizure as a sufficient descriptor may refer focal/generalized to the syndrome level. This should avoid quite some misuse of "generalized" (not only by generalists).

The relation of a seizure to sleep or time of day narrows the differential in TLOC quite a bit, helps with syndrome diagnosis, carries prognostic information that is going to have implications for driving [4] and will become relevant upon incorporation of Alzheimer's disease [5,6] into the classification of epilepsies. Where to put this information ?

[1] Burton RA (2009). On being certain: Believing you are right even when you're not. Macmillan.

[2] Dubner SJ, Levitt S (2014) The Three Hardest Words in the English Language. Why learning to say "I don't know" is one of the best things you can do. Episode 167. freakonomics.com/podcast/the-three-hardest-words-in-the-english-language/

[3] Thijs RD, Wagenaar WA, Middelkoop HA, Wieling W, van Dijk JG (2008). Transient loss of consciousness through the eyes of a witness. Neurology, 71(21), 1713-1718.

[4] Lawn ND, Pang EW, Lee J., Dunne JW (2023). First seizure from sleep: clinical features and prognosis. Epilepsia, 64(10), 2714-2724.

[5] Larner AJ & Marson AG (2011). Epileptic seizures in Alzheimer's disease: another fine MESS? Journal of Alzheimer's Disease, 25(3), 417-419.

[6] Baker JFW (2019). A Study of Interactions Between Memory Disorders and Epilepsy: Epileptic Seizures in Dementia, Contrasted with Transient Epileptic Amnesia. Thesis. University of Exeter or Baker J et al. (2019). Seizure, 71, 83-92.

Reviewer-2: The patient reports episodes starting with seeing colored dots and stripes on the left side. The patient cannot recall what happened after that, but eyewitnesses report that the patient does not respond to verbal and tactile stimuli, turns the head to the left, becomes stiff and then has jerks in all limbs. The seizure is classified as Focal-to-bilateral tonic-clonic seizure (FBTC; 1.4) with: elementary visual aura on the left side, versive to left, loss of awareness & responsiveness, bilateral tonic-clonic.

I am afraid this may bring some confusion when classifying the seizures in patients like this, because some of the seizures will certainly stop before becoming bilateral tonic-clonic (which is the first goal of our antiseizure medications!). Should then these patients be considered as having two seizure types ? (Focal to bilateral tonic-clonic (FBTC), AND Focal Impaired consciousness (FIC?)). In practice, what most people do is to classify as the most frequent situation (usually focal impaired consciousness (FIC) without focal to bilateral) and then add FBTC as a descriptor to mention that, sometimes, the seizures do progress to bilateral tonic-clonic. It would be interesting to learn from the authors whether they think this should be modified or commented upon in the discussion.

**Reflex seizures**

#1. If we can add reflex tonic seizure to tonic seizure classification and also reflex myoclonic seizure.

#22. Reflex seizures, particularly those triggered by specific stimuli, have been well-documented and are significant in certain populations. Incorporating reflex seizures into the classification would make it more comprehensive and provide a better tool for managing patients with photosensitive or other reflex epilepsy types.

Reviewer-1: Another seizure type I don’t see addressed are reflex or stimulus induced seizures. While these are features of certain epilepsies, they are inseparable elements of the individual seizure events in these patients. It seems that a seizure classification should account for this.

**Focal**

#15: Can we include spasms/ tonic seizures in focal seizure clarification just to emphasise that these seizure types not always generalised in nature.

#36: I agree that in the seizure classification, focal seizures now include tonic, and myoclonic, and epileptic spasms which were previously classified as only generalized.

**Generalized**

#8: I appreciate the addition of myoclonic-tonic-clonic and absence-tonic-clonic along the lines of focal-tonic-clonic. There should be a category of clonic-tonic-clonic, albeit rare, but should be a part of the new expansion.

#21: The basic version graphic seems to divide generalised seizures into 'absence seizures' or 'motor seizures'. Given absences often have motor components I wonder whether this is an unhelpful simplification as will imply to the reader absences with motor elements cannot be absences. An example of an alternative simplified group here might be:

Absence seizures

Tonic-clonic seizures

Other generalized seizures

#22: Adding rare seizure types, such as clonic-tonic-clonic and tonic-atonic seizures, would provide further clarity and utility in both research and clinical practice.

#27: I strongly agree with the subclassification of generalized seizures, and also with delegating them to the expanded version. However, having MYOCLONIC seizures in figure 1 could be helpful, as most first generalized seizures are seen by generalists, rather than epileptologists. Forgetting to ask or for myoclonic seizures remains an important factor in the misclassification of JME [1] and as "myoclonic or absence …" they carry independent prognostic information [2]. Further, myoclonic seizures should be accounted for, when valproic acid is to be avoided [3].

[1] Syvertsen M, Hellum MK, Hansen G, Edland A, Nakken KO, Selmer KK, Koht J (2017). Prevalence of juvenile myoclonic epilepsy in people <30 years of age-A population-based study in Norway. Epilepsia, 58(1), 105-112.

[2] Bonnett LJ, Tudur Smith C, Smith D, Williamson PR, Chadwick D, Marson AG (2014). Time to 12-month remission and treatment failure for generalised and unclassified epilepsy. J Neurol Neurosurg Psychiatry, 85(6), 603-610.

[3] Cerulli Irelli E, Cocchi E, Morano A, Gesche J, Caraballo RH, Lattanzi S, Strigaro G, Catania C, Ferlazzo E, Pascarella A, Casciato S, Quarato P, Pizzanelli C, Pulitano P, Giuliano L, Viola V, Mostacci B, Fortunato F, Marini C, Di Gennaro G, Gambardella A, Labate A,Operto FF, Giallonardo AT, Baykan B, Beier CP, Di Bonaventura C; Women With Epilepsy Treatment Options and Research (WETOR) Study Group. Levetiracetam vs Lamotrigine as first-line antiseizure medication in female patients with idiopathic generalized epilepsy. JAMA Neurol, 80(11), 1174-1181.

**Absence seizures**

#4: I am particularly thankful for the recent definitive classification of absence seizures. Previously categorized merely as generalized non-motor seizures, this redefinition acknowledges their distinct characteristics, including symptoms like eye-blinking, which were often overlooked or underestimated. This clarification is a significant step forward and aligns with the clinical realities I face, where absence seizures present unique diagnostic and management challenges. The recognition of specific seizure phenomena, like eye-blinking during absence seizures, enhances our diagnostic accuracy and enriches our understanding, ultimately benefiting the comprehensive care we strive to provide.

#36: Absence seizures are only described as generalized and are not recognized as focal seizures. Since atypical absence seizures can be seen in frontal lobe epilepsy, I think the atypical absence seizures should be included in not only generalized seizures but also focal seizures.

#38: Since during a seizure with eyelid myoclonia (without corresponding absence) there is no alteration of consciousness/responsiveness/awareness, it is my opinion that this type of seizure should be included among motor seizures ("other than tonic-clonic") [maybe coded as EM; 2.2.1.8], while eyelid myoclonus with concomitant absence will constitute a subtype of absences (with associated motor manifestations) [linked to the code EMA; 2.1.4]

#43: Remove nonmotor when categorizing absence seizures: Good – not a big change – people don’t actually call them generalised non-motor seizures anyway.

Adding absence-to-tonic-clonic seizure: Fine.

**Tonic seizures / sub-types of tonic seizures.**

#10.

In the CDKL5 Deficiency Disorder (CDD), there is a very characteristic seizure type characterized by the **hypermotor-tonic-spasms sequence**.

It was first described in 2011, see Klein KM Yendle SC Harvey AS Antony JH Wallace G Bienvenu T Scheffer IE. A distinctive seizure type in patients with CDKL5 mutations: Hypermotor-tonic-spasms sequence. Neurology. 2011

More than half of CDD patients present either hypermotor-tonic-spasm seizures **or tonic-spasm seizures** (ref: Demarest ST, Olson HE, et al 2019. CDKL5 deficiency disorder: Relationship between genotype, epilepsy, cortical visual impairment, and development. Epilepsia)

These characteristic seizures in CDD patients pose challenges for seizure counting and classification in trials.

This is a request to please provide guidance in the ILAE seizure classification for how to classify these seizures: e.g. **a subtype of tonic seizures**, a subtype of epileptic spams, a separate type of seizure.

One good example for how to classify some sequence seizures is the classification of “2.2.2.3. Absence-to-tonic-clonic seizure” as a subtype of GTC seizures in Table 1. A possibility would be to similarly include the tonic-to-spasm seizure so often seen in CDD patients under "2.2.1. Generalized motor seizures – other than tonic-clonic”.

But wherever you place it, my request is to please consider the inclusion of the tonic-to-spasm seizure because it is so common in CDD patients and it will make our clinical trials easier if it would have a direct map into the updated ILAE seizure classification.

#41.

My only comment relates to the lack of clear distinction in adults between generalised epileptic spasms and generalised tonic seizures. In adults they have striking overlapping features, clinical-EEG-surface EMG, and patients may have a continuum of brief (spasm) to longer duration (tonic seizure) events that differ only in their duration.

The supplement 3 description already hints at this overlap:

**Generalised epilepstic spasms** *“Subtle forms of spasms, with minimal / discrete manifestations may occur, including head nodding, grimacing, smiling, or chin movement”*.

**Generalised tonic seizure** *“They may be subtle, with slow upward eye rolling or deviation, at times with facial grimace or flexor movements of the head and/or trunk*”

**Spasms**

#23: It is confusing that 'spasms' have been categorized differently in generalized and focal seizures. We would appreciate more clarity regarding this.

#30: I believe the information that the seizures of the 14-month-old girls were (focal) spasms is equally important and influential on treatment as that of the 3-month-old boy. It seems unjustified that focal spasms are a descriptor and generalized spasms are a classifier. I do not know how I would deal with that in the classification, since focal seizures are so many.

#43: Epileptic spasms - incorporating epileptic spasms as a semiological descriptor for focal seizures and for seizures unknown whether focal or generalised. Retain epileptic spasms as a seizure type for generalised seizures. This will be problematic I think. I think of epileptic spasms as individual seizures that occur in periodic clusters. So to me this is mixing up two different concepts within focal seizures. I think epileptic spasms can be both a focal seizure type and also a focal semiological descriptor.

1. Focal Epileptic Spasms examples:
   1. The child has movement of only one arm (so unilateral) and the EEG only has discharges with the clinical change from the appropriate hemisphere. These can occur in clusters but they are each individual seizures lasting 1s or so. They have 15 focal epileptic spasms within a period of 5 min.
   2. The child has bilateral limb movement and the EEG shows bilateral discharges but the spasm is originating from a tuber. They have 10 focal epileptic spasms within a period of 3 min

Focal seizure with epileptic spasms as a semiological descriptor. The child stills and has impaired consciousness for 30 seconds then head deviates and has periodic spasms (clinically either bilateral or unilateral) over a period of 60 seconds but they remain unconscious during this time. So this I would consider a 90 min focal seizure with epileptic spasms as part of the semiology.

1. **Terms and abbreviations**

#5: It would have been nice to have a vowel in FPC to allow easy reference to focal preserved consciousness seizure and focal impaired consciousness seizures (FIC) without having to say the whole phrase when discussing with colleagues and trainees. Consider focal aware consciousness (FAC); note that this may help patients and their family understand that they can have a focal seizure where they appear conscious with eyes open and may even interact partly with the environment, but are not aware for FIC vs FAC seizures.

#6: (The updated classification) may bring terminology into simpler or more common usage. Hopefully, the energy to re-label and re-educate will be justified by greater accuracy and understanding.

#9: I suggest to replace "Focal Preserved Consciousness Seizure" and "Focal Impaired Consciousness Seizure" with "Focal Conscious Seizure" and " Focal Unconscious Seizure". Although I acknowledge that the latter may not be entirely correct in all cases, I would favour simplicity.

#17: When I was a neurology resident, I learned the term "crise parcial simples" and "crise parcial complexa". For me, it's short, simple and practical to use. The term "impaired consciousness" (in Portuguese - comprometimento de consciência) is long and tricky (because consciousness is partially preserved) and it's easier to speak "complex" - because it's really complex to talk about consciousness, awareness and responsiveness! I agree that focal is better than partial, so I would rather say "complex focal seizure" - in portuguese - crise focal complexa (CFC) - and "simple focal seizure" - in portuguese crise focal simples (CFS). The acronym in Portuguese is easier- CFC, CFS.

#22: The simplification of terms, such as replacing “Focal Preserved Consciousness Seizure” with "Focal Conscious Seizure" or "Focal Unconscious Seizure," could make the classification easier to use in practice. This streamlined language would help reduce confusion for both clinicians and patients, making the classification more accessible.

Many patients experience seizures without witnesses, and a category for "unobserved" seizures would help capture these events. Including such a category would provide a more complete diagnostic framework and allow for more accurate seizure tracking in clinical care.

#23: We propose to retain the terms focal onset and generalized onset as they help to deicide about the choice of anti-seizure medications. While sodium channel blockers work well against focal onset epilepsy., we know that drugs working through GABAergic system like valproate work well against generalized onset epilepsy.

We propose to change the term "focal to bilateral tonic-clonic seizures" to "focal-to-bilateral motor seizures" as all may not have 'tonic-clonic' seizures.

#24: The 2017 classification has not been well accepted by neurologist because the previous focal simple and complex were better understood.

#30: I have read comments asking “simple and complex partial seizures” back. These are very bad terms, they are inaccurate, and require specific translation (change in the actual meaning) for people to understand. I am certain people only like them because they were used to them. It would be a step backward to go back to this kind of terms.

#43: I understand the rationale for this and don’t feel strongly one way or the other. This isn’t a change in concept - it is just a change in terminology. From a SNOMED perspective we added Generalised Seizures and Focal Seizures as synonyms for the concept of Generalised-onset seizures and Focal-onset seizures because we believed that is practically what people say. Defining what these concepts are is more important than what they are called.

#44: “Unknown”, is a statement of what we don’t know and not a feature of the seizure. It adds unnecessary complexity. We don’t have to state what we don’t know. Worse, it implies that our job is done, when it isn’t : we still have work to do in classifying the seizure.

“Unclassified”. A classification includes things that can be classified. How can it include something that is not classified? It’s an oxymoron. This adds complexity and achieves nothing. Like “Unknown”, we don’t have to state what we don’t know and we certainly should not base a major division on what we don’t know. However, in effect, no-one has used it clinically since it’s appearance in 1981. More importantly, it again implies that the seizure fits into a category when it doesn’t. It implies our work is done when it isn’t.

Dropping of the word “onset” is a pity. It obviated the need to explain that the onset is what matters and removed the confusion regarding focal to bilateral tonic-clonic seizures. Removing “onset” invites the use of “generalised” to mean something other than generalised onset, and reopens the door to the confusion that “secondarily generalised seizures” caused. In everyday usage, the “onset” word can be dropped.

I could point out that the obvious counterpart of “bilateral” is “unilateral”, which would more logically replace “focal”, which Gastaut discarded because it implied a pinpoint onset rather than onset within a network, but that would be bridge too far.

1. **Consciousness vs. awareness**

#6: One of the changes is introduction of the term “consciousness” in place of “awareness.” Alteration of consciousness always has been a key classifier of seizures, but the term has not before been in the name of the seizure type (for example, “temporal lobe seizure,” “psychomotor seizure,” “complex partial seizure”). The 2017 Task Force thought that a typical seizure with automatisms or freezing of activity would not be perceived by the public as loss or alteration of consciousness. In the popular view, loss of consciousness conveys someone lying on the floor “out cold.” We looked for surrogates. Possibilities were awareness, responsiveness, memory, any or all of which can occur during a seizure. Responsiveness is of great operational importance because its loss is what crashes the car. However, responsiveness usually cannot be assayed retrospectively, unless someone was present to test it at the time of a seizure. Therefore, we chose awareness during the event as a surrogate. We also made note that many languages did not make a clear distinction between “awareness” and “consciousness.”

#19: Probably the most debated point in the classification of the type of seizure is the level of consciousness in focal seizures. It is difficult to find a single word that allows establishing the idea of what this means, especially when applying it in different languages. Therefore, it is only necessary to clarify what consciousness means for epilepsy, to establish the concept.

Proposal:

Consciousness in epilepsy is understood as when a patient maintains three characteristics during his seizure:

Responsiveness

Full memory of the event

Knowledge of self and environment

One of them is not enough, all three must be present, if one of them is not present, then the patient will have altered consciousness. It must be established that knowing the consciousness during a focal seizure requires an active and dynamic evaluation during the seizure by a witness.

#20: In my view, the previous classification's exclusive focus on "awareness" was a significant weakness. It has been counterintuitive to classify a responsive individual as "unaware" simply because memory encoding or consolidation during the ictal period is impaired. Therefore, I appreciate the return to earlier classifications that consider both awareness and responsiveness as operational criteria for consciousness.

#25: The panel is congratulated with the proposed simplification, in particular by avoiding confusion about awareness and consciousness (and its translation).

#29: Consciousness vs. Awareness: There are numerous debates surrounding the concepts of "consciousness" and "awareness" that extend beyond mere linguistic variations. Consciousness encompasses a vast domain that remains largely unexplored and intangible. A simplistic aspect of this is "memory," which was not addressed in this revision. Evaluating consciousness, particularly in younger children and infants, is more complex due to the significant influence of developmental processes on consciousness compared to awareness.

#35: Awareness and responsiveness are used to characterize consciousness. It may be difficult for an eyewitness to describe/appreciate responsiveness. The preferred word may be arousability which is better understood by laypersons.

#38: I genuinely appreciate the effort of the working group to update and simplify the classification of epileptic seizures. I agree with most of the proposed changes, in particular to re-adopt the term “consciousness” that is more widely used in medical language – not only in clinical neurology – respect to “awareness”, allowing a more homogeneous language particularly in the context of differential diagnosis (e.g. with syncope and other transitory “loss of consciousness” events).

#39: The definition of impairment of consciousness should not be considered as a dichotomy, but as a sign that is instaurated in a temporal variable and that can have a graduation in the temporal variable between mild, moderate and severe, taking into account its dynamic evolution during the temporal evolution of the seizure.

#43: Consciousness is used as a classifier instead of awareness with consciousness operationally defined by awareness and responsiveness

Again this is not really about concepts (at least not from the perspective of the majority of people that use these terms) it is simply about neurologists wanting the word that is used to describe the concept being the most accurate representation of what is actually happening to the individual. To me this debate is really purely about differences in opinions of “experts” with the majority of non expert neurologists seeing it as very esoteric. Whether the “correct” terminology is awareness, consciousness, dyscognitive, dissociative or what ever is not the important issue. At the end of the day it really depends on the age of the individual, the ability of the individual to describe what is happening, the ability of the witnesses to describe what is happening, whether responsiveness and awareness testing is done during the event and how well it is done, if it was captured on a video, on an EEG or both etc. And depending on a lot of these aspects different neurologists would classify the event in different ways at different times. The “TRUTH” about what is going on in an individual seizure is often difficult to get to and to agree upon. My opinion is that this proposal puts too much emphasis on getting to the “TRUTH” instead of just realising in many cases you won’t be able to do that for each individual seizure anyway. So whether you use awareness or consciousness in the big picture it doesn’t really matter – what does matter is that people understand the overall concept and use the same terminology for it. People are just getting their heads around awareness - changing it now is not going to be helpful.

Regardless of who is correct here, the differences in opinion were clearly discussed in the 2017 taskforce as there is a whole section about it in the 2017 paper. The arguments made then for awareness rather than consciousness are just as valid now as they were then (in fact some of the same reasons are being used for justifying consciousness now that were used to justify awareness then! – eg. language translation). The only substantial difference now is it is different group of people with a different perspective from the 2017 commission. So no new evidence just different people who think their way is the right way. Now they may be correct but I don’t think that is actually what is important here. Academic neurologists can debate the differences in meaning between these terms and how that relates to seizures but the rest of the world’s clinicians just see those subtle differences as esoteric. It doesn’t matter for everybody else -they just need a term to call it - they are not that bothered by what it is called and awareness is very easy for them to understand. Consciousness is not and my experience with how people understand “consciousness” is not consistent with what you say in the proposal. In fact, just for fun, I recently asked 20 different clinicians (who are not neurologists) around my department what does consciousness means – they conceptualise it as relating to alertness not awareness or responsiveness really.

Not everyone is ever going to be happy in the epilepsy world about either option but changing it now – when the present terms are only just getting embedded - will decrease the ILAE credibility. I understand that people have VERY STRONG opinions on this. As I have said I don’t, and had they used consciousness in 2017 I would have made that work and who knows maybe it would have been better – but they didn’t and now is not the time to change it.

I have been trying to think of a compromise that would (like getting rid of “onset” above) not be too big of a deal or a huge change in terminology – simply a nudge in the closer direction without actually using consciousness as a term. What about using this:

• Focal Impaired Awareness and/or Responsiveness Seizures (keep the acronym FIAS) and allowing the term Focal Impaired Consciousness seizure to also be used as a synonym – so an equivalent term

• Focal aware and responsive seizures (keep the acronym FAS) and allowing the term focal preserved consciousness seizure to also be used as a synonym.

The beauty of this approach is it doesn’t throw the baby out with the bath water and it also (I think) incorporates the concerns against just using awareness. The other advantage is that the name itself will be more understandable to people with epilepsy as you have suggested in your proposal that consciousness would have to be explained to them. It is pretty wordy though…..

#44. The “new” definition of consciousness is the same as the 1981 definition (not a bad thing). Awareness is not a synonym of memory but the document states that when taking the history, it is. Why use “awareness” at all? Why not just call it memory or recall? Great to see the comeback of “consciousness”. No longer will patients who are unaware they have had a seizure be unaware or aware they have had an unaware seizure!

Reviewer-1: The authors eliminated “dyscognitive” which they imply was a neologism they wished to avoid, which is understandable, but it served a purpose. “Awareness” has also proved inadequate and not completely accurate, so best to see it replaced. “Consciousness” seems a reasonable alternative but still seems problematic - the word in English implies a level of alertness, and impaired consciousness implies a decreased alertness level. It will be interesting to see what the larger community says when released for feedback.

Reviewer-2: The ‘return’ of the term consciousness, with crucial understanding that it encompasses either awareness, responsiveness or both is very welcome.

1. **Observable vs. motor**

#6: A seizure with no observable manifestations probably is sensory, emotional or autonomic. We thought it was worth specifying which of these was present at the start of the seizure, but apparently the updated version does not, and lumps them all under “without observable manifestations.”

#20: Definition “observable”: The term "observable" requires a clear definition and possibly revision (e.g. by “objective”). It appears to describe objectively verifiable aspects of a seizure, as opposed to elements accessible only through subjective introspection. What is considered "observable" varies depending on the degree of scrutiny applied during observation. This issue is not limited to cognitive phenomena, which may or may not be observed depending on patient interaction with their environment, but also extends to autonomic signs. For instance, when should a documented heart rate be classified as ictal tachycardia? Definitions of ictal tachycardia vary widely across research studies. Is ictal sweating considered observable only when it becomes visible, or if it is just detected through electrodermal activity (EDA) measurements? How observable is hypersalivation? Is "urinary urge" really an observable or subjective phenomenon?

The term “observable” can imply that a semiological sign has been directly observed (e.g., through inspection) or that it could be observed given the application of specific methodologies. If “observable” implies the potential for observation, this would lead to speculative classifications. Conversely, if actual observation is required, could the use of new techniques, such as wearable devices, shift a seizure classification from “not observable” to “observable”? Such methodological advancements may obscure the accurate assessment of epilepsy progression or outcomes if applied, for example, to outcome evaluations based on the resulting classification.

#26: - The removal of the terms non motor is not completely useful under the clinical viewpoint. It is true that some seizure types that are usually inserted among the non-motor seizures have associated motor manifestations (descriptors) but the identification of the predominant clinical manifestations as “non motor” may have relevant prognostic implications. Perhaps, the prefix “predominant” might help (e,g,“predominantly non motor generalized seizure”).

- The text does not sufficiently explain the concept of “observable” and “not observable signs” and only a table lists them. Non “observable signs” should be better characterized in the text. Perhaps the term “self-reported” should be more adapt. The class of “possibly observable” signs creates confusion. I think that they may be inserted among the observable ones. Further examples in the text, including a clinical vignette, might be useful to realize these aims.

#29: Observable vs. Non-observable Dichotomy: While this distinction is recognized, it may not be as clinically useful or practical as the differentiation between motor and non-motor functions. Several considerations regarding this rephrasing include:

- Terminology: Alternatives such as "observable-unobservable distinction" or "observable-latent variable dichotomy" may better convey the conceptual divide.

- Conceptual vs. Empirical: It is crucial to differentiate between conceptual distinctions and their empirical applicability; while theoretically valid, the observable-nonobservable dichotomy may have limited practical utility in clinical settings compared to other frameworks.

- Continuum vs. Dichotomy: Some scholars argue that observable and non-observable phenomena exist on a continuum rather than as strict categories, suggesting that certain variables may possess both observable and non-observable components.

- Context-Dependent: The relevance of this dichotomy may vary based on specific contexts, research questions, and theoretical frameworks. In summary, while the observable-nonobservable dichotomy serves as a conceptual distinction in literature, its practical utility and terminology may differ based on context and research focus.

#30: As “motor vs nonmotor” has its problems, also do “with or without observable manifestations”. If a witness detected a seizure, there must be an observable manifestation. I understood that a seizure without observable manifestations would only include purely sensitive and/or some autonomic seizures and unperceived seizures with post ictal manifestations. It would define if other people perceive the seizures, or the only one who perceives them is the patient. This new categorization could help to define social consequences of seizures. However, I believe the new classification as “with or without observable manifestations” will be confusing to implement. Moreover, it is not justified in the text. The authors present good reasons against the “motor vs non-motor”, but they don’t justify why they introduced a new and seemingly hard to implement classification (“observable”). In addition, whether manifestations are observed or not depends on the observer. A trained epileptologist will probably observe more signs than the regular seizure witness. Patient companions would probably vary a lot in terms of what manifestations they are able to catch. It makes even harder to implement a classification that implies that a manifestation is “observable”. In the second example case, even the authors seem to use “observable” inconsistently, since “auditory aura” is encompassed in “observable manifestations”. In sum, I believe “observable or not” adds an unnecessary label of complexity and should be removed. The authors may simply remove “motor vs non-motor” without an obligation to add something instead. It would be easier for healthcare providers to describe focal preserved consciousness seizures with sensory phenomena only, when that is the case, than to think they should always classify if the manifestations are “observable or not”.

#32: Sub-classifier (2): Replace the motor versus nonmotor sub-classification within focal seizures and within seizures unknown whether focal or generalized, with a sub-classification distinguishing between seizures with observable manifestations and those without describe seizure semiology in chronological sequence, depicting the sequence of seizure phenomena.

#39: The division between motor and non-motor events remains useful, since motor manifestations and their subtypes can have a high regionalising value in focal semiology and in the choice of appropriate medication in the subtypes of genetic generalised epilepsies (myoclonic motor, generalised tonic-clonic, atonic, negative myoclonic, tonic-clonic myoclonic, among others).

#43: The motor vr non-motor dichotomy is replaced by observable vs non-observable manifestations

This is another BIG change that just doesn’t seem necessary to me. It is also a conceptual change not just a terminology change. I understand where the drug trial people are coming from but why not just use those terms in drug trials? – they don’t have to be ILAE classification terminology in drug trials as long as it relates back to ILAE classification.

In my experience, many people, even experts didn’t use the terms non-motor onset and motor-onset correctly - they just say motor seizures and nonmotor seizure and really use motor for a seizure with predominant motor signs regardless of it there was a sensory aura before which spread to the motor cortex and use non-motor for seizures that don’t have any motor signs. So you could argue that is pretty much what you are proposing but just calling them different terms and adding a few observable semiological features which are not motor. I think that is too big of a change.

I think a compromise and less radical change would be just going to motor and nonmotor and getting rid of the onset - that would be an incremental step in that direction without completely throwing out the baby again!

#44: Observable v non-observable. Great for clinical trials but less useful in clinical practice.

Reviewer-1: Use of the term “observable” for subjective auras seems inaccurate. While these are crucial for localization, they are not really “observable” other than through patient reporting. Perhaps the term “auras and observable features” would account for this.

1. **Semiology: first vs. sequence**

#6: Focal and unknown categories now mention sequential semiological classifications. Of course, these should be in the documentation of seizures, but they are not seizure CLASSIFICATIONS. It would be impossible to list all sequential symptoms and signs and all propagation patterns during seizures. The 2017 Task Force chose to list only the most important one, which is focal to bilateral tonic-clonic. That seizure type is maintained.

#20: I also acknowledge that using the first semiological manifestation of a seizure as a classifier, as used in the last classification, has both advantages and disadvantages. The initial observed element may not fully represent the global phenomenology of focal seizures, which can vary greatly depending on the propagation pathways and the gradual involvement of multiple symptomatogenic areas by ictal activity.

#39: We believe that the first motor or non-motor sign in the symptomatic cluster of focal epilepsies is practically relevant for the initial therapeutic decision. Although the identification of these signs is key to the diagnosis, their influence on the choice and implementation of treatment seems to be less determinant in clinical practice.

#43: The chronological sequence of seizure semiology is used to describe seizures, rather than relying solely on the first sign.

This is fine - it is really just an addition rather than a change – please note though this is very unlikely to get into coding terms – it is too complex. I think Artificial Intelligence will even find it difficult to sort out from a BIG DATA perspective! For the individual patient though in their notes that seems fine – I think it is only neurologists who are likely to go to this level of detail.

Reviewer-2: It is also a strength of this renewed initiative the encouragement to describe seizure evolution, as it provides an immediate ‘topographical map’ of epileptogenic networks that is very useful for teaching, EEG interpretation and, above all, to raise the possibility of surgical considerations.

1. **Semiology table**

#5: In the section on "Cognitive & language phenomena," I recommend adding 2 categories: 1) confusion, 2) Other focal cognitive deficits (eg, anosognosia, apraxia, neglect,). #1 is common and can occur in the absence of aphasia or amnesia. Further, patients can have amnesia without confusion. #2 is rare but these deficits have been reported in the literature. Since focal seizures may inhibit normal neural function, a variety of negative cognitive deficits that are topographically related to the site of cortical ictal activity. The present Somatotopic Modifiers do not account for the variety of potential ictal cognitive deficits.

#20: The term “aphasic” is proposed to be used at the same level as “autonomic.” I strongly recommend retaining previous terms such as “cognitive” or “affective” at this classification level, as aphasia is just one possible manifestation of cognitive ictal impairment, similar to sweating being one manifestation of an autonomic phenomenon.

#23: In the section 1 " elementary motor phenomena" there are 2 terms which mean the same- ' eye deviation' and 'versive'.

#30: “Aura” is one of those words that seem good to those who are used to using it. However, it is a very bad word for a seizure classification, because it is one of those words that constitute an unnecessary exclusive language for the specific community, it has spiritual connotations, and it is more appropriately used in migraine. Sensitive manifestations or phenomena is already used to refer to the old “aura”. I suggest changing “7. Indescribable aura” for “indescribable sensation”. In the examples, I would also recommend changing auditory and visual aura for another word (might be auditory/visual phenomena, hallucinations, or manifestations). For internal consistency, I believe phenomena should be used, because it was used in table 2.

#32 (as related to #31): I am a practicing neurologist in Northern part of India for 25 yrs and my patients are from Haryana , adjacent districts of Rajasthan and Punjab, more than 25000 patients were treated and being treated in our center in last couple of decades therefore a classification at hand which should give me to address the change of semiology over time during a seizure, the individual awareness during a seizure, the reactivity to external stimuli in a clinical meaning, the existence of potentially dangerous behavior, the individual memorization of whether a seizure has occurred at all, and a quick and clear communication with subsequent action to prevent the seizure to happen further and protect the patient, therefore aura is very important part to understand and awareness about it is of utmost concern in seizure management lesions and guidance for the caregivers rightly described in this position paper.

#39: It would be advisable to simplify the categories of somatotopic mediators of seizures to make them more accessible and practical in regions with less training in seizure analysis. This simplification would facilitate understanding and clinical management in areas with limited training resources in this area, allowing for more efficient care.

#42: My only minor comment is that table 2 is incomplete (I can think up half a dozen terms that have been missed) and it is likely to evolve especially with better post-ictal assessments, Do you think it would be worthwhile pointing this out rather than wait for people to point out, for example, that deja vecu is not there and is not the same as déjà vu etc. etc. or would you rather wait for suggestions from the community to expand the table?

Reviewer-1: A few nit-picky items were noted. Although Table 2 is lengthy, there are a few omissions (ictal micturition, polydipsia, borborymi, the “regarding the hand” sign, and there could be additional) - perhaps a category of “other” would allow for capturing these and future novel findings. Also, the subclassifications selected for some items as listed in the table is arguable (is ictal orgasm purely affective or in part autonomic or sensory, is ictal fear in part autonomic). While some semiologic features are clearly classifiable, others are less clean; perhaps they call for utilization of the old rubric “Experiential” instead. One such symptom reported to me by some patients with temporal regional onset seizures are what they can only describe as “waves” - in the categories provided I am not sure where this would belong. Finally, I have concerns about the translatability of some of these terms which is a stated goal of this revision - hopefully this list was vetted across languages. Also in this table, in order to remain true to the stated goal of avoiding neologisms, probably best to eliminate the term “dysmnesia”.

1. **Negative myoclonus**

#6: A new seizure type of negative myoclonic seizures has been added, and I have no quarrel with that. Over the past years, I have received several e-mails nominating other seizure types, including tonic-atonic, atonic-tonic, absence to tonic-clonic. At some point, a classification becomes unwieldy, so inclusionary decisions had to be made.

#9: The term "Generalized Negative Myoclonic Seizure" deserves some further explanation / description.

#12: Negative myoclonic seizure need to be more clarified in terms of etiology for its varied presentations.

#14: I have a challenge with negative myoclonus - the identification of it. I work in a resource constraint environment, where EEG assessments are not routinely available and so will tend to use clinical evaluation.

#22: Although the addition of negative myoclonus is a welcome change, ensuring clear differentiation between negative and positive myoclonus, especially in the context of PME and GGE, would greatly improve diagnostic accuracy.

#29: Epileptic Negative Myoclonus: This electroclinical entity can only be effectively studied using video-EEG monitoring with an EMG extra-electrode, which is often unavailable in resource-limited countries.

#32: Negative myoclonus: Include the recognition of negative myoclonus within the seizure classification is an excellent approach.

#43: Negative myoclonus is recognised as a seizure type

Good.

1. **Miscellaneous**

**Graphical design**

#11: This classification will appear on thousands of PowerPoint slides. May I humbly suggest consulting a graphic designer for the final print version of the manuscript to enhance the style and legibility of Figures 1 and 2.

#22: Given how widely this classification will be used, it might be worth considering the involvement of a graphic designer to refine the figures, particularly Figures 1 and 2. A clearer, more engaging layout would not only improve the readability of these visuals but also enhance their effectiveness in educational and clinical settings.

#27: I wish to support the comments by Marian Galovic (#11) and Pasquale Striano (#22), the ILAE should carefully select the information to be presented in Figures 1 and 2 and tailor them to the intended audience.

Reviewer-1: I would consider re-ordering Figures 1 and 2, listing the most common seizure types on top, followed by the rarer ones. It is odd to see “other” tonic clonic over tonic clonic in the Basic, and atypical absence and absence w eyelid myoclonias at the top and tonic clonic, the most devastating and more common generalized seizure type at the bottom.

**How-to?**

#12: In the case of only autonomic semiology, where do they need to be placed?

#29: Queries:

1. Regarding the working group, how was selection bias addressed? Were participants matched in terms of experience, knowledge, training, etc.?

2. Given that one of the main objectives of the 2017 version was to ensure practicality and feasibility in resource-limited countries, where English is not always the native language, was a linguist expert included in your working groups?

3. In the review process, were papers distributed randomly between the two raters (SB and ET)?

4. Could “non-motor” be replaced with "non-convulsive"? This terminology seems more applicable.

#35: Another issue is whether we should change the seizure classification of well-controlled patients who were categorized using the 2017 classification. In other words, how do you transport patients from one classification to next?

#37: It is still not clear for me how it will be classified when the patient was fully aware and responsive during the seizure but totally amnestic to the event. There are 2 scenarios in this condition. First the patient may continue the automatisms and/or inappropriate behaviour but responsive and then amnestic or may act totally normal during the event but not remember anything at all (like Dr.Z the Jackson's famous patient. Taylor D. JNNP1980, 43, 758-767 ).

**Definitions**

#38: In the “supplementary-3-definitions-of-generalized-seizures2” file, in the description of the EMA it seems that it is mandatory to observe “the extension of the head” associated with eyelid jerks and upward eyeballs deviation, however this is not always the case, thus I suggest to be less assertive “[...] with simultaneous upward deviation of the eyeballs, sometimes associated to extension of the head [...]”

Regarding Myoclonic Absence Seizure (MA; 2.1.3), I suggest to add a statement as “Polygraphic recordings of EMG with EEG is recommended for ictal recordings” (similarly to what has been done for GMA; 2.2.1.7).

Reviewer-1: The definition of “focal” in the second paragraph of the Results may be too restrictive in requiring seizure onset of individual seizures to be limited to one hemisphere. For example, some seizures are bitemporal implying robust bihemispheric networks, where it remains unclear which if either temporal region is the inciter. Nonetheless, nobody would consider such seizures “generalized”. Perhaps the definition should be changed to those with seizure onset network confined to one hemisphere or to limited regions of both hemispheres.

#43: The definitions of generalised epileptic seizure types

These all look good, there is just one thing which is a bit of a bugbear of mine probably because my doctoral thesis was on absence seizure semiology …..

- Generalised clonic seizure:
  - I think that this seizure type is seriously in need of being more rigorously defined as it is not consistently understood by epileptologists
  - It is clear to me that clinicians use the term for different concepts (seizure semiologies). This only became apparent to me when I have been reviewing seizure videos for journals and reviewing seizure descriptions and classifications for drug trials. Prior to that I assumed everyone thought the same way I did.
  - I think that most epileptologists would classify a seizure where the individual drops to the ground with no tonic component, is completely unconscious and starts to have bilateral rhythmic myoclonic jerks that are time locked to the GSW as a generalised clonic seizure
  - However, for a seizure where the individual maintains postural tone, has a decree of impaired awareness/responsiveness and has significant rhythmic myoclonic jerks of the arms (which do not significantly lift up) epileptologists will not be consistent and it may get classified as either a:
    - myoclonic absence seizure
    - generalised clonic seizure
    - absence seizure with myoclonic components
  - The problem is that your present definition would include all of these above seizure concepts as it doesn’t mention postural tone or level of consciousness.
  - I would argue that seizures with bilateral “myoclonic jerks that are regular and repetitive at a low frequency (typically 0.2 to 5Hz and involve the same muscle groups” that are time locked to the GSW/PSW” with impaired awareness/responsiveness
    - where the individual can maintain posture (ie sitting or standing) and has impaired awareness and responsiveness should be classified as an absence seizure with myoclonic features
    - where the individual drops to the ground and cannot maintain posture and is unconscious should be classified as a generalised clonic seizure.

My rationale for this suggestion is:

- It is all a matter of severity
- It is generally widely accepted that typical absence seizures can have subtle myoclonic features of the eyes, nose, lips, eyebrows, face
- It is also not that uncommon to get subtle myoclonic jerks of limbs in absence seizures
- When the myoclonic jerks are significant AND there is tonic contraction of the arms in an absence seizure we call the seizure a myoclonic absence.
- It doesn’t make sense to me then to take a seizure which is exactly the same in terms of posture, awareness/responsiveness and EEG but because the jerks are more significant than can be found in more widely accepted absence seizures but not as significant as found in a myoclonic absence we call it something completely different – a generalised clonic seizure. It should be still considered an absence seizure.

**SNOMED**

#43.

It is almost the end of 2024 and we have only just got the 2017 (7 years on) concepts and terminology in SNOMED –that process has taken 2 years of work and it will take more time (years) before those updated names will find their way into the end user interfaces (EPIC etc.) HPO only updated their concepts and terminology a few years ago. INSERM and Orphanet are still updating their terminology. ICD updates are years from becoming used in many countries

Another major concern relates to the Ontology of what you are proposing – I don’t think it is sound and won’t get into the electronic medical records because the current international medical terminology systems (SNOMED and HPO) will not allow some of the terms to be put in. The 2017 classification had the same problem and we have had to modify it to get it into SNOMED and HPO. It would be better if we just had an ILAE classification scheme that had a better ontology so that the terms could go directly into electronic records. I have explained this more in detail below.

The ONTOLOGY - Here is where I outline my concerns regarding the ontology which is laid out in Table 2. This is important because it impacts on what terminology can actually be used within an electronic health record. So the ILAE can decide all it likes but if the term will never go into an electronic health record what is the point of the ILAE having it – this is about implementation.

There are a few things in Table 2 here that will not work in an ontology (SNOMED or HPO for example).

- 1. You can’t have two concepts called the same thing with the same definition. So you can’t have this:

2.2.2 Generalised tonic-clonic seizure

2.2.2.1 Generalised tonic-clonic seizure

That just won’t work – you would either need to rename and define the 2.2.2 term or the 2.2.2.1 term which I think would add unnecessary groupers (a SNOMED concept) . It would be easier and make more sense to go:

2.2. Generalized motor seizures (not including Absence Seizures)

~~2.2.1. Generalized motor seizures – other than tonic-clonic~~

2.2.1. Generalized myoclonic seizure (GM)

2.2.2. Generalized clonic seizure (GC)

2.2.3. Generalized negative myoclonic seizure (GNM)

2.2.4. Generalized epileptic spasm (GES)

2.2.5. Generalized tonic seizure (GT)

2.2.6. Generalized atonic seizure (GA)

2.2.7. Generalized myoclonic-atonic seizure (GMA)

2.2.8. Generalized tonic-clonic seizure (GTC)

2.2.9. Generalized Myoclonic tonic-clonic seizure

2.2.10. Generalized Absence-to-tonic-clonic seizure

- 1. Also from an Ontological perspective – you can’t have **unknown** in SNOMED or HPO. Please note this is not because the people on the ILAE SNOMED taskforce thought it was the way to go – it is simply what has to be done within an “is always a” ontology – so it is about the way the ontologies work and makes sense for data collection within Big Data systems -nothing to do with epilepsy but imperative to consider when devising classification ontologies (hierarchies). If something is **unknown** then it should be classified as the parent concept - so for example - your 1.3. Focal Unknown State of Consciousness Seizure would be classified as simply a Focal Seizure as the definition of focal seizure implies that you may or may not know about state of consciousness - if you do know about the state of consciousness then you would classify it as the subclassification of Focal Impaired Consciousness Seizure – so the table should really ideally look like this for focal:

**1. Focal Seizure**

1.1. Focal Preserved Consciousness seizure (FPC)

1.1. – 1. With observable manifestations

1.1. – 2. Without observable manifestations

1.2 Focal Impaired Consciousness seizure (FIC)

1.2. – 1. With additional* observable manifestations

1.2. – 2. Without additional* observable manifestations

1.3 Focal-to-bilateral tonic-clonic seizure (FBTC)

Although I would argue this would be better:

**1. Focal Seizure**

1.1. Focal Preserved Consciousness seizure (FPC)

1.1. – 1. With observable manifestations

1.1. – 2. Without observable manifestations

1.2 Focal Impaired Consciousness seizure (FIC)

1.2. – 1. With additional* observable manifestations

1.2.1 Focal-to-bilateral tonic-clonic seizure (FBTC)

1.2. – 2. Without additional* observable manifestations

That is not what we have done is SNOMED though (which is the first example) – after emails with Bob Fisher it was decided that a FBTCS could be either an aware or an impaired awareness seizure as the awareness was just ascertained up to the point of involving bilateral hemispheric networks – that didn’t make sense to me personally as I would say a FBTCS definitely has impaired awareness (or consciousness).

**PWE**

#28: Epileptic seizure are hard to understand until now, specially the patients and the community. Epilepsy awareness are known only for it's word, and basic information, but in reality, specification of epilepsy are far to understand. The doctors in public hospitals did not define to the patient the classification of epilepsy he/she has. Just basic check up only perform, but communication between the doctor and the patient, by asking or revealing is not practiced to define epilepsy and do's and dont's of AED the patient would take. Encouraging and helping PWE to spread awareness are only pinned during Epilepsy Awareness Week, but nothing else. If i am right or not, sorry for my comments but just to express my side, our side, as a person with epilepsy.

**Methodology**

#43: Systematic Reviews comments - although I think the idea of doing a systematic review is good - I don’t think that including reviews and opinion pieces or abstracts is good science. At the end of the day people are more likely to complain about something they don’t like than something they like – so it isn’t surprising that the majority of the opinion pieces are negative. I think this aspect of the review and proposal introduces unnecessary and unhelpful bias against the 2017 classification and won’t reflect what the majority of people think. I personally think that aspect of the review should be removed. It is just some more “experts’ opinion – they can provide feedback to you if they feel strongly but shouldn’t influence unduly the process.

Reviewer-1: Utilizing the customary structure of scientific papers to position papers is a challenge. The “Methods” section contains numerous items that the end of the day are findings and derived solutions stemming from the analysis of identified problem areas, this fitting better with the concept of “results” rather than recitation of the methods and processes used. The reading experience as a result is a bit awkward. Perhaps there should be a methods section dryly covering how the literature search and deliberations proceeded, then a results section broken down into problem spots identified from the literature and in group discussions, followed by the solutions identified which resulted in the final product.

**SPANISH TRANSLATION**

#19

1) Escriben conciencia (con c) y consciencia (sc) en el mismo documento

Considero que debe ser escrito en español con sc ya que consciencia tiene un sentido mas amplio de la incapacidad del paciente pararesponder y no tener consciencia, que es el planteamiento del término. Conciencia con c es mas de uso para un término moral (eso quedará en tu conciencia).

2)En el apartado de crisis de ausencia se escribe crisis con mioclonía palpebral son o sin alteración de consciencia. Lo cual es un error, en este apartado se trata de las mioclonías palpebrales con o sin ausencia (como lo había descrito originalmente Peter Jeavons), es un error que debe corregirse

En el último aparatado de la clasificación, considero debe cambiarse el termino en español de No clasificable por el de No clasificada. Un concepto es que no sea posible clasificar una crisis epiléptica, pero la mayoría de las veces es no clasificada porque no tenemos en el momento de clasificar, claridad de la semiología de la crisis del paciente.

**ITALIAN TRANSLATION**

#22: Some of the terms used in the translated versions, like "consapevolezza integra" in the Italian version, could benefit from minor adjustments. Terms such as "coscienza intatta" or "coscienza preservata" would likely be clearer and more consistent with clinical terminology, making the classification more user-friendly for non-English-speaking clinicians.

#38: I want to signal that the Italian translation of the new proposal does not take into account at all the reasons (extensively discussed in the paper and very much agreeable, in my opinion) that led the authors to use again the term "consciousness" (instead of “awareness”) and to replace "focal onset seizure" and "generalized onset seizure" with "focal seizure" and "generalized seizure". In fact, in the Italian translation of table 1, Figure 1, and Figure 2 is perpetuated the OLD translation, retaining “consapevolezza” (that is not used in any other neurological field in Italy, but has been adopted “believing it aligns with ILAE position paper, despite this interpretation being incorrect”), instead of “coscienza”. Idem for Figure 1, in which there are still the terms “Esordio Focale” and “Esordio Generalizzato” that the current proposal suggest as “misleading”; thus the new terms should be “Crisi focale” and “Crisi generalizzata”.

1. **Outside the scope**

#7: I suggest to create a new topic with genetic epileptic syndromes like Dravet, and Alice in the wonderland.

#31: In this expanded seizures classification, it is better to classify first whether the seizures are organic, functional or seizure mimic. Then under the heading of organic seizures,the expanded seizures classification can be followed.

#32: Lastly the symptomatic epilepsies both acute and chronic due to CNS infection in this part of the country is a major concern and comprising about 40% cause of epileptic seizure so important cause is different fever related seizure including FIRES in young children therefore symptomatic and localization related epilepsy might be given a place in the new classification.

#36:

I agree with the idea of grouping drug-resistant epilepsy syndromes occurring in newborns and infants under the name of EIDEE(Early Infantile Developmental and Epileptic Encephalopathy). This idea includes Ohtahara syndrome and EME. These two syndromes have a common finding of burst-suppression (BS) pattern on interictal EEG, and it can be difficult to determine which one they belong to. The current proposal states that EEG findings show either BS pattern or multifocal discharges in EIDEE. Shouldn't we distinguish and classify EIDEE with BS pattern from EIDEE without BS pattern? There may be cases where the SB pattern was not confirmed because sufficient EEG tests were not performed in early infancy. However, distinguishing cases where the SB pattern was clearly recognized is an important point when considering the relationship with the subsequent course, treatment, and gene mutations.

Thank you for summarizing the diagnostic criteria for Dravet syndrome in an easy-to-understand manner. I thought that the wide range of ages of onset and the concise diagnostic guidelines are valuable guidelines that lead to early specific treatment through earlier diagnosis. On the other hand, I was concerned that epileptic spasms (ES) were listed as exclusionary criteria. Although rare, ES can be seen during the course of Dravet syndrome. I propose that the diagnosis of exclusion be changed to when ES is present early in the course of epilepsy.

**New proposals**

*Crating a totally new framework – outside the scope of the public hearing.*

#2

I am aware that the ideal approach to a classification is to combine a simplicity for a broad applicability on the one hand and a granularity which captures the complexity of real life on the other hand. In daily practice I often wished to have a classification at hand which allowed me to address

1. the change of semiology over time during a seizure

2. the individual awareness during a seizure

3. the reactivity to external stimuli in a clinical meaning

4. the existence of potentially dangerous behaviour.

5. the individual memorization of whether a seizure has occurred at all

6. a quick and clear communication and documentation tool

This would have been helpful as

A. it might have implications on driving a car

B. taking care of small children

C. might interfere with workplace activities

D. might impact on seizure counting (predisposition to underreporting)

E. impacts on design of interventional studies

F. epidemiology in general and relative frequency in an individual

G. neurological topography to associate specific semiological patterns to certain brain areas as a prerequisite for epilepsy surgery

The complexity I would like to address is AWARENESS A:

A1->1 (A11) means that the patient is aware at the beginning (A1) and at the end (-1) of a seizure.

A1->0 (A10) means that the patient is aware at the beginning (A1) but not at the end (-0) of a seizure.

A0->0 (A00) means that the patient is not aware at the beginning (A0) nor at the end (-0) of a seizure.

RESPONSIVENESS R:

R1->1 (R11) means that the patient is responsive at the beginning (R1) and at the end (-1) of a seizure.

R1->0 (R10) means that the patient is responsive at the beginning (R1) but not at the end (-0) of a seizure.

R0->0 (R00) means that the patient is not responsive at the beginning (R0) nor at the end (-0) of a seizure.

Rx->x (Rxx) the patient does not know whether she/he is responsive, there are no witnesses so far.

BEHAVIOR as part of seizure semiology, BUT NOT DANGEROUS B:

B1->1 (B11) means that the patient shows no dangerous behavior (e.g. putting boots into the oven) at the beginning (R1) and at the end (-1) of a seizure.

B1->0 (B10) means that the patient shows no dangerous behavior (e.g. putting boots into the oven) at the beginning (R1) but does so at the end (-0) of a seizure.

B0->0 (B00) means that the patient shows dangerous behavior at the beginning (B0) and at the end (-0) of a seizure (i.e., throughout the seizure).

MEMORIZATION whether a seizure has occurred at all M:

M1 the patient remembers/”memorizes” that a seizure has occurred.

M1d the patient directly remembers/”memorizes” that a seizure has occurred, i.e., the patient recalls parts of semiology.

M1i the patient INdirectly remembers/”memorizes” that a seizure has occurred, i.e., the patient concludes from aspects other than semiology that a seizure must have occurred, e.g. blood and saliva on the pillow.

M1x the patient remembers the seizure as such but it is unknown by which mechanism.

Examples:

A11 R11 B11 M1d: This is a seizure in which the patient is

• fully aware from the beginning throughout the whole seizure, (A1->1, A11)

• always responsive, (R1->1, R11)

• always with adequate behaviour, (B1->1, B11)

• memorizes directly that the she/he had a seizure and can report this (M1d)

A10 R11 B11 M1: This is a seizure in which the patient is

• fully aware only at the beginning of the seizure, (A1->0, A10)

• always responsive, (R1->1, R11)

• always with adequate behaviour (B1->1, B11)

• memorizes that the she/he had a seizure and can report this (M1x). The physician does not know how the patient remebers that there was a seizure.

A10 R10 Bxx M0:This is a seizure in which the patient is

• fully aware only at the beginning of the seizure (A1->0, A10)

• responsive only at the beginning of the seizure (R1->0, R10)

• the behaviour is not reported, no witnesses available (Bxx)

• does not memorize that the she/he had a seizure (M0)

A10 R10 Bx0 M0: This is a seizure in which the patient is

• fully aware only at the beginning of the seizure (A1->0, A10)

• responsive only at the beginning of the seizure (R1->0, R10)

• the behaviour is not reported at the beginning, but later shows inadequate behaviour (e.g. puts boots into the oven), (Bx0)

• does not memorize that the she/he had a seizure (M0)

Clinical examples:

1. For a young mother it would be quite a success to improve from R11 Bx0 to R11 B11 as her child is no longer endangered by inadequate dangerous behaviour.

2. A pharmaceutical company may wish to include only patients with Aany Rany Bany M1 to guarantee adequate seizure reporting and counting.

3. There should be no problems at a suitable workplace for a patient who has seizures classified as A11 R11 B11 M1d.

4. In presurgical evaluation, A10 R10 B11 M1 has a different meaning than A00 R00 B10 M0.

Possibilities for extension:

In presurgical evaluation it might be highly relevant if the patient speaks during the seizure, therefore:

S11 (S11) means that the patient speaks adequately throughout the seizure, an involvement of the speech relevant eloquent cortex does not occur.

S1->0 (S10) means that the patient speaks adequately at the beginning, but there is impairment of speech at the end of the seizure.

SX->X (SXX) Speech was not witnessed or tested during the seizure.

SX->X/1 (SXX/1) Speech was not witnessed or tested during the seizure. However, speech was normal in the postictal testing.

I do not yet know what “unknown” should exactly mean regarding consciousness:

a. We do not know whether the patient is aware during the seizure.

b. We do not know whether the patient is responsive during the seizure.

c. We do not know whether the patient memorizes her/his seizure.

d. Any combination of the above a – c.

#7:

I also suggest to differentiate epilepsy crises due to their origin in the brain with their more common phenotypes as like as frontal lobe epilepsies and parietal epilepsies.

#18:

I would like to share a few thoughts with you:

a. The authors correctly introduced the category “unknown” with the proposed classifiers, i.e., (1) focal and general, and (2) preserved or impaired consciousness. However, the classifier (3) with or without observable manifestations does not have this important category “unknown”. -> Could it be useful to extend the grammar of this taxonomy and provide the category of “unknown” to all three major classifiers in focal seizures?

b. In close relation to this, the authors proposed

1.1-1 with observable manifestations (CLASSIFIER)

1.1.-2 without observable manifestations (CLASSIFER)

1.1.-3 serial description (DESCRIPTOR)

-> I would like to suggest that CLASSIFIERS and DESCRIPTORS are not mixed in the taxonomical hierarchy. It should become clear from the taxonomic code what kind of information is provided or required. Could the following approach be helpful, please?

1.1 with observable manifestations (CLASSIFIER)

1.1.-D DESCRIPTOR of 1.1

1.2 without observable manifestations (CLASSIFER)

1.3 unknown (CLASSIFIER)

Alternatively: I (Roman 1) serial description (DESCRIPTOR), i.e. Roman numbers (I, II, III, ) for DESCRIPTORS.

c. The somatotopic modifiers under point 4 Cognitive & language phenomenon include aphasia,

-> Could it be useful to distinguish between “receptive aphasia”, “expressive aphasia”, “global aphasia”, “other”?

-> Could it be useful to include specific neuropsychological signs or syndromes (e.g. frontal disinhibition, Klüver-Bucy, severe agitation)?

-> Could it be useful to add “slowed thinking”?

-> Could it be useful to include “out of body experience”?

-> Could it be useful to include “feeling as if watching myself from behind/above”?

d. Should a somatotopic modifier of “behavor” be introduced or added to the cognitive group?

Could it be helpful to distinguish between “behavior: dangerous in activities of daily living”, e.g. putting boots into the oven during ictal confusion. Could it be helpful to state that the patients suffers from postictal depression or postictal aggressive behavior? – It seems to be warranted to include the postictal phase to the seizure classification as this has impact on patient management.

e. Could it be useful to include a category of “triggers”. – Triggers might help reduce seizure frequency simply by preventing the exposure to the trigger.

Sleep deprivation, exposure to flicker light, hyperventilation, Listening to certain music, reading, other.

(This could be implemented like this:

1.1 with observable manifestations

1.1.-D semiology description in chronological sequ.

1.1.-T relevant triggers)

f. The authors successfully applied the concept of consciousness with subdomains of awareness and responsiveness. However, in daily work for people with epilepsy it is highly relevant if the patient can recall the seizures as such. There are several situations in which the patient has a full memory for the time during the seizure and was always adequately responsive but herself/himself cannot recall that a seizure has taken place, e.g. with behavioral observable manifestation. Relatives frequently report “my son did not realize that there was a seizure. He remembers everything. At school they also report adequate responses, also at home, but I as his mother always detect them”.

This new proposal bears the chance to address all components of consciousness. Could it be helpful to characterize consciousness in the domains awareness, responsiveness, and recall of the events as such? (This could impact on design of studies.)

g. The classification aims to “establish a common language for all healthcare professionals (HCP) involved in epilepsy care”. Could it be helpful to think this highly important point in new ways? What kind of communication could prove useful between Epileptologist in epilepsy center with stereo-EEG (SEEG),

Epileptologist in epileptsy center with presurgical evaluation but without SEEG,

Epileptologist in a hospital without presurgical evaluation,

Epileptologist in personal praxis,

Epilepsy nurses dedicated to taking care of patients with epilepsy

General neurologist in hospital,

General neurologist in personal praxis,

Occupational physician deciding on whether a job is suitable or not.General practitioner, family doctor.

Physiotherapists, ergotherapists, logopedics, neuropsychologists, who train the patient according to the diagnoses established by physicians.

Nurses on other departments than neurology in hospital.

The list is still not complete, but a few of the above listed HCPs might have difficulties with optimizing their therapeutic approach when reading

“FPC 1.1 with observable manifestations: epigastric aura->oroalimentary automatisms + gestural automatisms with the right hand + preserved awareness and responsiveness” (example provided by authors)

In order to establish a communication system that provides all HCP with fast and relevant information there could be new approaches:

Could it be helpful to communicate “Focal seizure with preserved consciousness: always aware, always responsive, recalls the occurance of a seizure as such, no dangerous behavior”? [(A1-R1-M1-B1)]

The more HCPs understand our language and the more epileptologists transfer their findings into ADLs the more adequate medical support and social integration will be given to people with epilepsy.

#29: Proposals:

1. We propose developing structured interviews and an educational module for patients and caregivers to enhance information gathering. Designing this as a smartphone application could be advantageous, and we believe collaborating with ILAE would be beneficial.

2. We suggest an AI-assisted program aimed at developing an application that will assist physicians in classifying seizures more effectively according to the updated ILAE guidelines.

3. Additionally, we propose integrating this updated epilepsy classification by ILAE with the latest version of ICD.
