# Supplementary material for: Updated classification of epileptic seizures: Position paper of the International League Against Epilepsy
Source: Epilepsia. 2025 Apr 23;66(6):1804–23. doi: 10.1111/epi.18338 (PMC12169392; doi:10.1111/epi.18338)
Supplement: Supplementary file 5 — Data S5. [file EPI-66-1804-s007.docx]

**Definitions of generalized epileptic seizure types**

**Typical absence seizure (TA; 3.1.1)** is a generalized epileptic seizure characterized by sudden onset, interruption of ongoing activities, a blank stare (loss of facial expression), and possibly a brief upward deviation of the eyes. Usually the patient is unresponsive; in most cases, awareness is impaired too. However, occasionally, after the seizure, patients may recall the ictal events (for example test words given during the seizure). Oral and/or manual automatisms occur in 86% of patients and eye involvement with blinking, eye opening, or subtle eyelid or perioral myoclonus in 76.5% of patients. There is immediate return to normal activity, although children may be momentarily confused as they reorient themselves. Duration is a few seconds to half a minute (median 7 s; range: 2-26 s*) but rarely they may last >30 s. Ictal EEG is characterized by regular, bilateral-synchronous (“generalized”) spike-waves. In the first seconds of seizure onset, the frequency of the spike-waves is around 3 Hz; range: 2.5–4 Hz in Childhood Absence Epilepsy (CAE), 3-5.5 Hz in Juvenile Absence Epilepsy (JAE). Disorganized discharges, defined by brief (<1 s) or transient interruptions in the ictal rhythm, or waveforms of different frequency or morphology are significantly less common in CAE than in JAE. The seizures are typically provoked by hyperventilation in most untreated patients with CAE. They may be provoked by intermittent photic stimulation too. In CAE, seizures typically occur multiple times per day but are often under-recognized. In JAE, typical absence seizures occur less than daily in the untreated state.

**Atypical absence seizure (AA; 3.1.2)** is a generalized seizure type characterized by episodes of impaired consciousness (awareness and/or responsiveness). Changes in tone (when present) are more pronounced than in typical absence seizures (for example head-drop as opposed to mild head retropulsion), and the onset and/or cessation is gradual (not abrupt). Duration is usually longer than of typical absence seizures, but with considerable overlap (median: 15 s; range: 2-10 s*). Ictal EEG shows irregular, bilateral synchronous and asynchronous/asymmetric spike-waves, with frequency lower than in typical absence (<2.5 Hz) and the ictal activity may be fragmented or include fast activity. Atypical absence seizures may occur in Lennox-Gastaut syndrome.

**Myoclonic absence seizure (MA; 3.1.3)** are absence seizures with abrupt onset and offset, associated with rhythmic 3-Hz jerks of the upper limbs, superimposed on tonic abduction of the arms during the seizure (giving a ratcheting appearance). The patient, if standing, typically bends forward during the seizure, but falling is uncommon. The myoclonic jerks are typically bilateral and symmetric but can be unilateral or asymmetric. Perioral myoclonia and rhythmic jerks of the head and legs may also occur. Impairment of consciousness varies from complete loss of awareness and responsiveness to retained awareness and responsiveness. Occasionally, autonomic manifestations, such as a change in breathing or urinary incontinence, or complex gestural automatisms, may be seen. Duration is typically 7–12 s, but occasionally longer (up to 60 s) and may occur multiple times per day. Ictal EEG shows regular 3Hz, bilateral-synchronous (“generalized”) spike-waves, time-locked with the myoclonic jerks. Myoclonic absence seizures occur in a variety of genetic conditions; this seizure type is mandatory for the diagnosis of epilepsy with myoclonic absence syndrome. Polygraphic recordings of EMG with EEG is recommended for ictal recordings.

**Eyelid myoclonia with / without absence (EMA; 3.1.4)**. Eyelid myoclonia, consists of brief, repetitive, and often rhythmic 3–6-Hz myoclonic jerks of the eyelids, often with simultaneous upward deviation of the eyeballs and extension of the head. Eyelid myoclonia can be associated with absences, but can occur without a corresponding absence. They are typically induced by involuntary or voluntary slow eye closure or exposure to bright sunlight. These seizures are very brief (median duration: 1.5 s; range: 0.5-8 s*) and occur multiple times each day, even many times per hour. Ictal EEG shows bilateral synchronous (“generalized”) fast spike activity or 3–6 Hz polyspike-and-wave discharges, typically elicited by eye closure and intermittent photic stimulation, especially in untreated patients. This seizure type is mandatory for the diagnosis of epilepsy with eyelid myoclonia (formerly called Jeavons syndrome).

**Generalized tonic-clonic seizure (GTC; 3.2)** consists of a tonic phase, with sustained muscle activity, followed by a clonic phase with progressive slowing of the clonic jerks, due to the gradual increase in the duration of the silent-periods interrupting the muscle activation, which eventually terminate the seizure. These motor phenomena are bilateral, but not always symmetric, and focal features (such as forced head version) may be observed in generalized tonic-clonic seizures. Typically, there is loss of consciousness during the seizure and in the postictal period. GTC seizures may be preceded by sporadic or irregular myoclonic jerks (Myoclonic-tonic-clonic seizure; 3.2.1) often seen in patients with Juvenile Myoclonic Epilepsy, or by an absence seizure (Absence-to-bilateral-tonic-clonic seizure; 3.2.2). Median duration of GTCS is 80 s (range: 57 – 102 s*). The ictal EEG is often obscured by movement artifact. Bilateral-synchronous (“generalized”) fast rhythmic spikes may be seen in the tonic stage, which is followed by bursts of spikes and slow-waves, synchronous with clonic jerks, during the clonic phase. A postictal period of generalized EEG suppression (PGES) or irregular, diffuse slow activity follows a GTC seizure. In idiopathic generalized epilepsies, GTC seizures often occur on awakening or with sleep deprivation. In focal conditions, the seizure is classified as Focal-to-bilateral tonic-clonic seizure (FBTC; 1.3). When the origin is unknown, the seizure is classified as Bilateral tonic-clonic seizure, unknown whether focal or generalized (BTC; 2.3). Tonic-clonic seizures have the highest associated morbidity and mortality, and represent the major risk factor of Sudden Unexpected Death in Epilepsy (SUDEP).

**Generalized myoclonic seizure (GM; 3.3.1).** Myoclonic jerks (a.k.a. myoclonus, plural: myoclonia) were defined as sudden, brief (lightening-like; <100-msec) involuntary, single or multiple irregular/arrhythmic contractions of muscles or muscle groups. When measured using surface electromyogram (EMG), their median duration was 80 ms (range: 30-140 ms*). Generalized myoclonic seizures are bilateral but can predominate on one side of the body, frequently involving the upper extremities. They can also involve the lower limbs and cause falls. Generalized myoclonic seizures can be reflex, triggered by photic stimulation or praxis. The typical ictal EEG correlate is bilateral-synchronous (“generalized”) polyspike-and-wave discharges (or spike-and-wave discharge), time-locked to the myoclonus. Generalized myoclonic seizures are mandatory for the diagnosis of Juvenile Myoclonic Epilepsy, and they may occur in other generalized epilepsies too. Note that unilateral myoclonic jerks can occur in focal seizures, in which case they are classified as focal seizures, and myoclonus added as a descriptor of seizure semiology.

**Generalized clonic seizure (GC; 3.3.2)** consists of myoclonic jerks that are regular and repetitive, at a relatively low frequency (typically 0.2-5 Hz) and involve the same muscle groups. Generalized clonic seizures are bilateral, but not always synchronous and symmetric. Duration is 4 s (range: 1 – 24 s*). Ictal EEG shows generalized spike-and-wave or polyspike-and-wave discharges, time-locked to the clonic jerks. Note that unilateral or asymmetric clonic phenomena can occur in focal seizures, in which case they are classified as focal seizures, and clonic is added as a descriptor of seizure semiology. Generalized clonic seizures should be distinguished from myoclonic absence seizures, which exhibit distinct movement patterns (see 2.1.3).

**Generalized negative myoclonic seizure (GNM; 3.3.3)** is defined as a brief interruption of muscle tone (<500 ms), causing a sudden, brief lapse in movement that may grossly appear like a myoclonic jerk. Generalized negative myoclonic seizures are bilateral, but not always synchronous and symmetric. To document negative myoclonus, it is often necessary to instruct the patient to perform a voluntary muscle activation, such as lifting the arms. The EEG correlate is a spike-wave or a low-amplitude sharp-transient. The onset of the EMG silent-period is related to a negative component of the spike on the EEG, occurring before the slow wave. In progressive myoclonic epilepsies, a cortical involvement has been demonstrated in cortical reflex negative myoclonus. Unilateral or asymmetric negative myoclonus can occur in focal seizures, in which case they are classified as focal seizures, and negative myoclonus is added as a descriptor of seizure semiology. Subcortical negative myoclonus may occur in metabolic encephalopathies.

**Generalized epileptic spasms (GES; 3.3.4)** consist of brief contractions of axial (predominantly truncal and proximal) muscles, each typically lasting ≤2 s (median: 1 s; range: 0.4-2 s*), causing abduction and extension of both arms, hip flexion and nodding. Subtle forms of spasms, with minimal / discrete manifestations may occur, including head nodding, grimacing, smiling, or chin movement. Epileptic spasms usually occur in clusters, often upon awakening, with increasing prominence of the motor features through the cluster, often over a period of minutes (although clusters may last 30 min or longer). The ictal EEG correlate is characterized by a high amplitude, generalized, sharp or slow wave that is followed by low amplitude, fast activity or a brief, diffuse electrodecrement. Surface EMG helps to distinguish epileptic spasms from myoclonic seizures and tonic seizures. The EMG of an epileptic spasm has a typical diamond-shape (gradual increase and gradual decrease in amplitude). Epileptic spasms are mandatory for the diagnosis of Infantile Epileptic Spasm Syndrome. Epileptic spasms may occur in focal / structural epilepsies, in which case they may appear unilateral or asymmetric. However, the bilateral symmetric semiology does not rule out the focal origin, and a complex multimodal investigation, including video-polygraphic recordings, neuroimaging, laboratory and genetic tests are needed to correctly classify epileptic spasm. When epileptic spasm occurs in a focal condition, it is classified as focal seizure, and epileptic spasm is added as a descriptor of seizure semiology. When the origin is uncertain, epileptic spasm is classified as unknown whether focal or generalized.

**Generalized tonic seizure (GT; 3.3.5)** is defined as sustained muscular contraction resulting in stiffness or tense posture, that usually causes an extension, but it may also affect the flexor muscles. The median duration of generalized tonic seizures is 8 s (range: 3 – 51 s*). Generalized tonic seizures are bilateral, but not necessarily symmetrical. They may be subtle, with slow upward eye rolling or deviation, at times with facial grimace or flexor movements of the head and/or trunk, or more clinically obvious, with a brief cry, apnea, abduction, and elevation of the limbs with a vibratory component and bilateral fist clenching. If occurring while the patient is standing, they may forcefully throw the patient off balance, leading to a fall with the patient often sustaining an injury. Tonic seizures can be precipitated by startle. During sleep, generalized tonic seizures may be very subtle and not recognized by the family members, and therefore need polygraphic recording of sleep to identify and quantify seizure frequency. Generalized tonic seizures may be preceded or followed by spasms (colloquially termed “tonic spasms”), a myoclonic jerk (“myoclonic-tonic seizure”), or a hyperkinetic seizure followed by a spasm (“hypermotor-tonic-spasms”). The ictal EEG pattern of tonic seizures consists of a burst of bilateral 10 Hz or higher frequency fast activity with a recruiting rhythm, an initial diffuse decrement followed by gradual increase in amplitude. Generalized tonic seizures are mandatory for diagnosis of Lennox-Gastaut syndrome. Focal tonic ictal phenomena may occur in focal seizures, in which case they are classified as focal seizures, and tonic is added as a descriptor of the seizure semiology.

**Generalized atonic seizure (GA; 3.3.6)** is defined as a sudden loss or decrease in muscle tone, without apparent preceding myoclonic or tonic event, involving the head, trunk, jaw, and limbs. Due to the loss of postural tone, atonic seizures frequently cause falls and injury. The median duration is 1 s (range: 0.5 – 13 s*). Polygraphic recordings including surface EMG of the antagonist muscles are useful to document this seizure type. Atonic seizures are often observed in Lennox-Gastaut syndrome. They may occur in focal epilepsies too, in which case they are classified as focal seizures and atonic is added as a descriptor of the seizure semiology.

**Generalized myoclonic-atonic seizure (GMA; 3.3.7)** is characterized by a brief myoclonic jerk affecting the proximal muscles, often associated with a slight vocalization, followed by a very brief atonic component, which may be subtle, with a head nod, or more prominent, with an abrupt fall. Median duration is 1.25 s (range: 0.7 - 1.5 s*). Ictal EEG shows bilateral-synchronous (“generalized”) polyspike or spike discharges with the myoclonus, followed by a high-voltage slow-wave accompanying the atonic component. Polygraphic recordings of EMG with EEG is recommended for ictal recordings. Myoclonic–atonic seizures are mandatory for diagnosis of Epilepsy with Myoclonic-Atonic Seizures (Doose syndrome).

*The definitions are adapted from the following papers:*

Fisher RS, Cross JH, D'Souza C, French JA, Haut SR, Higurashi N, Hirsch E, Jansen FE, Lagae L, Moshé SL, Peltola J, Roulet Perez E, Scheffer IE, Schulze-Bonhage A, Somerville E, Sperling M, Yacubian EM, Zuberi SM. Instruction manual for the ILAE 2017 operational classification of seizure types. Epilepsia. 2017;58:531-542. doi: 10.1111/epi.13671.

Zuberi SM, Wirrell E, Yozawitz E, Wilmshurst JM, Specchio N, Riney K, Pressler R, Auvin S, Samia P, Hirsch E, Galicchio S, Triki C, Snead OC, Wiebe S, Cross JH, Tinuper P, Scheffer IE, Perucca E, Moshé SL, Nabbout R. ILAE classification and definition of epilepsy syndromes with onset in neonates and infants: Position statement by the ILAE Task Force on Nosology and Definitions. Epilepsia. 2022;63:1349-1397. doi: 10.1111/epi.17239.

Specchio N, Wirrell EC, Scheffer IE, Nabbout R, Riney K, Samia P, Guerreiro M, Gwer S, Zuberi SM, Wilmshurst JM, Yozawitz E, Pressler R, Hirsch E, Wiebe S, Cross HJ, Perucca E, Moshé SL, Tinuper P, Auvin S. International League Against Epilepsy classification and definition of epilepsy syndromes with onset in childhood: Position paper by the ILAE Task Force on Nosology and Definitions. Epilepsia. 2022;63:1398-1442. doi: 10.1111/epi.17241.

Hirsch E, French J, Scheffer IE, Bogacz A, Alsaadi T, Sperling MR, Abdulla F, Zuberi SM, Trinka E, Specchio N, Somerville E, Samia P, Riney K, Nabbout R, Jain S, Wilmshurst JM, Auvin S, Wiebe S, Perucca E, Moshé SL, Tinuper P, Wirrell EC. ILAE definition of the Idiopathic Generalized Epilepsy Syndromes: Position statement by the ILAE Task Force on Nosology and Definitions. Epilepsia. 2022;63:1475-1499. doi: 10.1111/epi.17236.

Beniczky S, Tatum WO, Blumenfeld H, Stefan H, Mani J, Maillard L, Fahoum F, Vinayan KP, Mayor LC, Vlachou M, Seeck M, Ryvlin P, Kahane P. Seizure semiology: ILAE glossary of terms and their significance. Epileptic Disord. 2022;24:447-495. doi: 10.1684/epd.2022.1430.

Meritam Larsen P, Wüstenhagen S, Terney D, Gardella E, Aurlien H, Beniczky S. Duration of epileptic seizure types: A data-driven approach. Epilepsia. 2023;64:469-478. doi: 10.1111/epi.17492.
