# Supplementary material for: Updated classification of epileptic seizures: Position paper of the International League Against Epilepsy
Source: Epilepsia. 2025 Apr 23;66(6):1804–23. doi: 10.1111/epi.18338 (PMC12169392; doi:10.1111/epi.18338)
Supplement: Supplementary file 6 — Data S6. [file EPI-66-1804-s003.pdf]

Supplementary Document 6

Translations

Languages page

In alphabetical order

Language		page
**Arabic**	....	**2**
**Chinese**	....	**5**
**Danish**	....	**8**
**French**	....	**11**
**German**	....	**14**
**Hungarian**	....	**17**
**Italian**	....	**20**
**Japanese**	....	**23**
**Korean**	....	**26**
**Portuguese**	....	**29**
**Romanian**	....	**32**
**Russian**	....	**35**
**Spanish**	....	**38**
**Ukrainian**	....	**41**

Arabic translation

التسلسل الهرمي التصنيفي لنوبات الصرع

1. بؤري(F)

1.1. نوبات بؤرية مع بقاء الوعي
1.2. نوبات بؤرية مع اضطراب الوعي
1.3.النوبة التوترية الرمعية من البؤرية إلى الثنائية

الوصف<br>الأساسى : مع مظاهر ملحوظة<br>بدون مظاهر ملحوظة<br>المفصل: وصف الْأَعْرَاض الْمَرَضِيَّة بالتسلسل الزمنى: (قائمة المصطلحات/ المسرد**) + معدلات التموضع الجسدى

2 . غير معروف ما إذا كان بؤريًا أم معمم(U)

2.1غير معروف ما إذا كان بؤريًا أم معممًا - مع بقاء الوعي
2.2غير معروف ما إذا كان بؤريًا أم معممًا - مع اضطراب الوعي
2.3غير معروف ما إذا كانت النوبات التوترية الارتجاجية بؤرية أم معممة

الوصف<br>الأساسى : مع مظاهر ملحوظة<br>بدون مظاهر ملحوظة<br>المفصل: وصف الْأَعْرَاض الْمَرَضِيَّة بالتسلسل الزمنى: (قائمة المصطلحات/ المسرد**) + معدلات التموضع الجسدى

3 معمم(G)

3.1.نوبات الغياب

3.1.1.نوبة غياب نمطية
3.1.2.نوبة غياب غير نمطية
3.1.3.نوبة غياب الرمع العضلي
3.1.4.رمع عضلي في الجفن مع / بدون غياب

3.2 . النوبة التوترية الارتجاجية المعممة
3.2.1 النوبة التوترية الارتجاجية الرمعية
3.2.2غياب إلى نوبة توترية ارتجاجية

3.3 النوبات الحركية المعممة الأخرى
3.3.1.نوبة الرمع العضلي المعمم
3.3.2النوبة التوترية المعممة
3.3.3 نوبة الرمع العضلي السلبي المعمم
3.3.4النوبة التقلصية المعممة
3.3.5النوبة التوترية المعممة.
3.3.6النوبة الارتخائية المعممة
3.3.7 النوبة الرمعية العضلية-الارتخائية المعممة

4.غير مصنف

*انظر الجدول 2 :المظاهر الإكلينيكية (السيميولوجيا).

**هذا مصطلح تجميعي، وليس مفهومًا محددًا.

النسخة الأساسية

معمم

نوبات الغياب

النوبات الحركية المعممة خلاف التوترية الارتجاجية

النوبات الحركية التوترية الارتجاجية المعممة

غير معروف

ما إذا كان بؤريًا أم معممًا

الوعي[1,3]
- *بقاء*
- *اضطراب*

غير معروف ما إذا كانت النوبات التوترية الارتجاجية بؤرية أم معممة

بؤري

الوعي[1,2]
- *بقاء*
- *اضطراب*

النوبة التوترية الارتجاجية من البؤرية

غير مصنف

الوصف الأساسي:

[4]مع مظاهر ملحوظة أو بدون مظاهر ملحوظة

دليل الأرقام بالشكل (1)

1. يتم تحديدها من الناحية التشغيلية من خلال الوعي والاستجابة.
2. عندما تكون حالة الوعي غير معروفة، يتم تصنيفها على أنها بؤرية (دون تحديد التصنيف الفرعي)
3. إذا كانت حالة الوعي غير معروفة، قم بتصنيفها على أنها غير معروفة سواء كانت بؤرية أو معممة (دون تحديد التصنيف الفرعي)
4. يتم التعرف بسهولة على المظاهر التي يمكن ملاحظتها من قبل شاهد عيان. قد تكون هذه حركية أو حبسية أو لاإرادية أو غيرها (انظر الجدول 2). يعتبر ضعف الوعي بمثابة مظهر يمكن ملاحظته. تظهر المصنفات (أنواع النوبات) باللون الأسود، بينما تظهر الواصفات باللون الأزرق.

تشير الخلفية الصفراء أن النوبات التوترية الرمعية الثنائية - المرتبطة بأعلى معدلات الإصابة بالأمراض والوفيات - يمكن أن تحدث في جميع فئات النوبات الرئيسية الثلاثة.

نسخة الموسعة

معمم

- نوبة الرمع العضلي المعمم[5]
- نوبة الرمع العضلي السلبي المعمم [5]
- التوترية الارتجاجية المعممة[5]
- نوبة تقلصية
- النوبة التوترية المعممة[5]
- النوبة الرمعية العضلية-الارتخائية المعممة
- النوبة الارتخائية المعممة [5]

- نوبة غياب نمطية
- نوبة غياب غير نمطية
- نوبة غياب الرمع العضلي
- نوبة رمع عضلي في الجفن مع / بدون غياب

النوبة التوترية الارتجاجية المعممة.
النوبة التوترية الارتجاجية الرمعية
نوبة غياب إلى نوبة توترية ارتجاجية.

غير معروف ما إذا كان بؤريًا أم معممًا

الوعي[1,3]
- ➢ بقاء
- ➢ اضطراب

غير معروف ما إذا كانت النوبات التوترية الارتجاجية بؤرية أم معممة

بؤري

الوعي[1,2]
- ➢ بقاء
- ➢ اضطراب

النوبة التوترية الارتجاجية من البؤرية

غير مصنف

التوصيفات المفصلة :

الْأَعْرَاض الْمَرَضِيَّة بالتسلسل الزمني [4]و تشمل : نوبة بؤرية تقلصية ,نوبة الرمع العضلي, النوبة التوترية& النوبة الارتجاجية[4] .

دليل الأرقام بالشكل (2)

1. يتم تحديدها من الناحية التشغيلية من خلال الوعي والاستجابة.
2. عندما تكون حالة الوعي غير معروفة، يتم تصنيفها على أنها بؤرية (دون تحديد التصنيف الفرعي)
3. إذا كانت حالة الوعي غير معروفة، قم بتصنيفها على أنها غير معروفة سواء كانت بؤرية أو معممة (دون تحديد التصنيف الفرعي)
4. تم وصفه باستخدام المصطلحات الواردة في مسرد ILAE السيميولوجي (انظر الجدول 2).
5. قد تحدث هذه الظواهر أيضًا في النوبات البؤرية (علي جانب واحد أو غير متماثل) كجزء من الوصف الإكلينيكي (سيميولوجيا) النوبة البؤرية

تظهر المصنفات (أنواع النوبات) باللون الأسود، بينما تظهر الواصفات باللون الأزرق.
تشير الخلفية الصفراء أن النوبات التوترية الرمعية الثنائية - المرتبطة بأعلى معدلات الإصابة بالأمراض والوفيات - يمكن أن تحدث في جميع فئات النوبات الرئيسية الثلاثة.

Chinese translation

1. 局灶性(F)

1.1. 局灶性意识保留发作 (FPC)

1.2. 局灶性意识障碍发作(FIC)

1.3. 局灶进展到双侧的强直-阵挛发作 (FBTC)

描述词

- *基本版本:*
  - *伴可观察到的症状*
  - *不伴可观察到的症状*
- *扩展版本:*
  - *以时间顺序罗列症状学描述信息:*
    症状学(术语表) + 躯体定位性描述词*

2. 未知是否为局灶性或全面性(U)

2.1. 未知是否为局灶性或全面性-意识保留发作 (PC)

2.2. 未知是否为局灶性或全面性-意识障碍发作 (IC)

2.3. 未知是否为局灶性或全面性-双侧的强直-阵挛发作 (BTC)

描述词

- *基础版本:*
  - *伴可观察到的症状*
  - *不伴可观察到的症状*
- *扩展版本:*
  - *以时间顺序罗列症状学描述信息:*
    症状学(术语表) + 躯体定位性描述词*

3. 全面性(G)

3.1. 失神发作 (AS)

3.1.1. 典型失神发作 (TA)

3.1.2. 不典型失神发作 (AA)

3.1.3. 肌阵挛失神发作(MA)

3.1.4. 眼睑肌阵挛伴/不伴失神发作 (EMA)

3.2. 全面性强直-阵挛发作(GTC)

3.2.1. 肌阵挛强直-阵挛发作

3.2.2. 失神进展到强直-阵挛发作

3.3. 其他全面性发作**

3.3.1. 全面性肌阵挛发作 (GM)

3.3.2. 全面性阵挛发作 (GC)

3.3.3. 全面性负性肌阵挛发作 (GNM)

3.3.4. 全面性癫痫性痉挛发作(GES)

3.3.5. 全面性强直发作 (GT)

3.3.6. 全面性失张力发作(GA)

3.3.7. 全面性肌阵挛-失张力发作 (GMA)

4. 无法分类的发作

*参考表2描述症状学特点。

**这是一个表述分组的术语，并非一个定义。

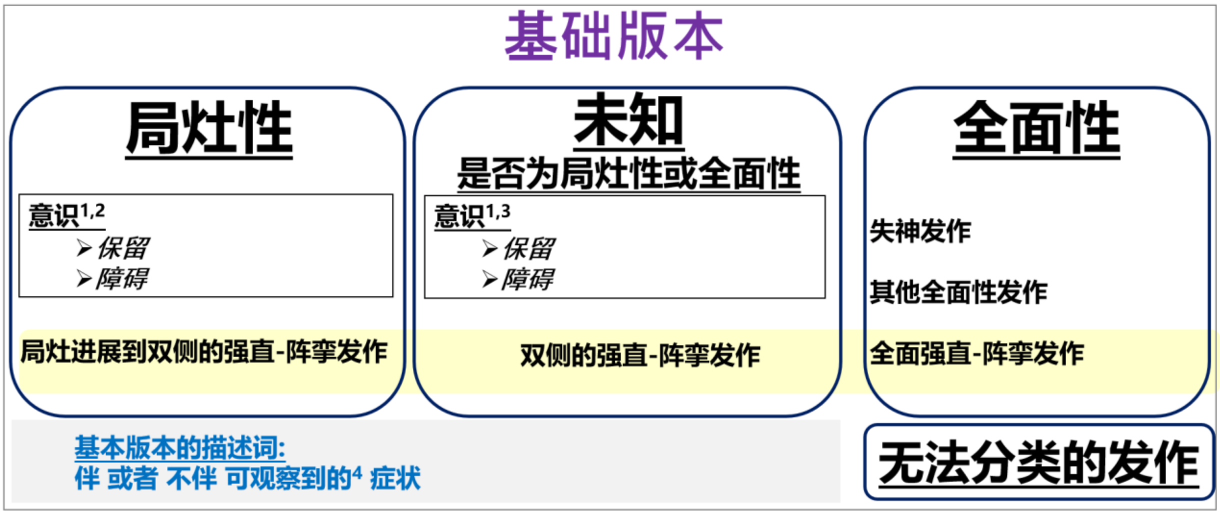

图1注释

1. 操作性定义为知觉和反应能力。
2. 当意识状态未知时，直接分类为局灶性（不特别指出意识的亚分类）
3. 当意识状态未知时，直接分类为未知是否为局灶性或全面性（不特别指出意识的亚分类）
4. 可观察到的症状为目击者很容易识别出的现象。这些可能是运动性的、失语性的、自主神经性的或其他的（参考表2）。意识障碍也是一种可观察到的症状。

分类信息（癫痫发作类型）以黑色显示，而描述信息以蓝色显示。 横向的黄色背景特别指出与死亡率最高度相关的-双侧的强直-阵挛发作-可以出现在所有三种主要发作类型下。

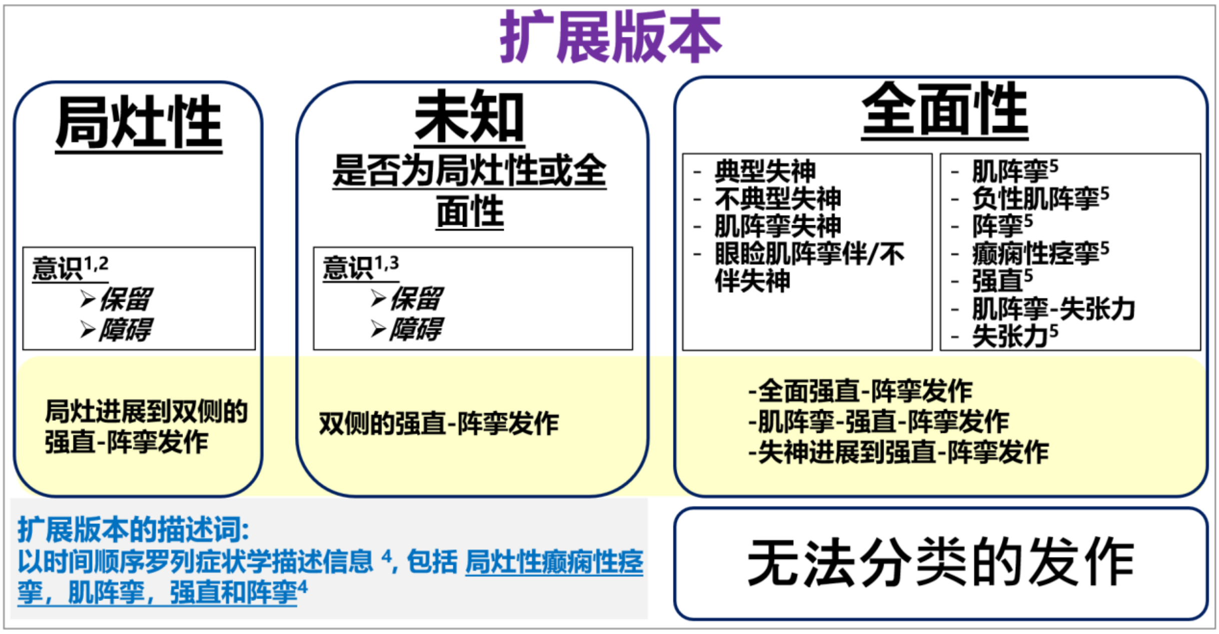

图2注释

1. 操作性定义为知觉和反应能力。
2. 当意识状态未知时，直接分类为局灶性（不特别指出意识的亚分类）
3. 当意识状态未知时，直接分类为未知是否为局灶性或全面性（不特别指出意识的亚分类）
4. 使用ILAE症状学术语表中的术语进行描述（参考表2）
5. 这些症状也可能出现于局灶性发作中 （通常是单侧的或者非对称性的），并作为局灶性发作的一部分症状学特点。

分类信息（癫痫发作类型）以黑色显示，而描述信息以蓝色显示 。 横向的黄色背景特别指出与死亡率最高度相关的-双侧的强直-阵挛发作-可以出现在所有三种主要发作类型下。

Danish translation

5. Fokalt

5.1. Fokalt uden bevidsthedspåvirkning
5.2. Fokalt med bevidsthedspåvirkning
5.3. Fokalt-til-bilateralt tonisk-klonisk

Beskrivelse

- *Basale:*
  - *Med observerbare manifestationer*
  - *Uden observerbare manifestationer*
- *Udvidede:*
  - *Semiologi beskrevet i kronologisk rækkefølge: Semiologiske fund + kropsdele*

6. Ukendt hvorvidt fokalt eller generaliseret

6.1. Ukendt om fokalt eller generaliseret - uden bevidsthedspåvirkning
6.2. Ukendt om fokalt eller generaliseret - med bevidsthedspåvirkning
6.3. Ukendt om fokalt eller generaliseret - bilateralt tonisk-klonisk

Beskrivelse

- *Basale:*
  - *Med observerbare manifestationer*
  - *Uden observerbare manifestationer*
- *Udvidede:*
  - *Semiologi beskrevet i kronologisk rækkefølge: Semiologiske fund + kropsdele*

7. Generaliseret

7.1. Absence anfald
- 7.1.1. Typisk absence
- 7.1.2. Atypisk absence
- 7.1.3. Myoklon absence
- 7.1.4. Øjenlågs myoklonier med/uden absence

7.2. Generaliseret tonisk-klonisk
- 7.2.1. Myoklont-til-tonisk-klonisk
- 7.2.2. Absence-til-tonisk-klonisk

7.3. Øvrige generaliserede anfald**
- 7.3.1. Generaliseret myoklont
- 7.3.2. Generaliseret klonisk
- 7.3.3. Generaliseret negativ myoklont
- 7.3.4. Generaliseret epileptisk spasme
- 7.3.5. Generaliseret tonisk
- 7.3.6. Generaliseret atonisk
- 7.3.7. Generaliseret myoklont-atonisk

8. **Uklassificeret**

*Se Tabel 2 - semiologiske fund.
**Dette er et udtryk for samling, ikke en defineret koncept.

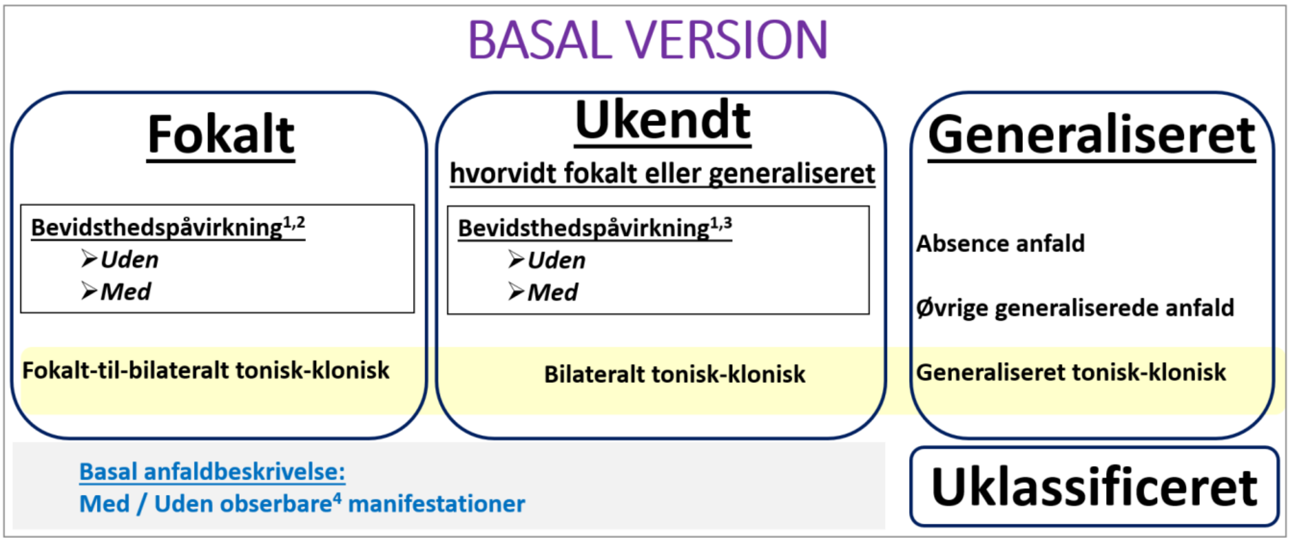

1. Bevidsthedspåvirkning er operationelt defineret ved manglende evne til at gengive begivenheder (amnesi) og ved manglende responsitivitet.
2. Når bevidsthedspåvirkning er ukendt, klassificeres anfaldet som fokalt (uden at specificere underklassifikationen).
3. Hvis bevidsthedspåvirkning er ukendt, klassificeres anfaldet som ukendt hvorvidt det er fokalt eller generaliseret (uden at specificere yderligere).
4. Observerbare manifestationer kan genkendes let af et øjenvidne. Disse kan være motoriske, afatiske, autonome eller andre (se Tabel 2). Påvirket bevidsthed klassificeres som en observerbar manifestation.

Anfaldstyper vises i sort, mens beskrivelse af anfald vises i blåt. Den horisontale gule baggrund i figurerne understreger, at bilaterale tonisk-kloniske anfald er forbundet med høj sygelighed og dødelighed. Denne anfaldstype kan forekomme i alle tre hovedanfaldsklasser.

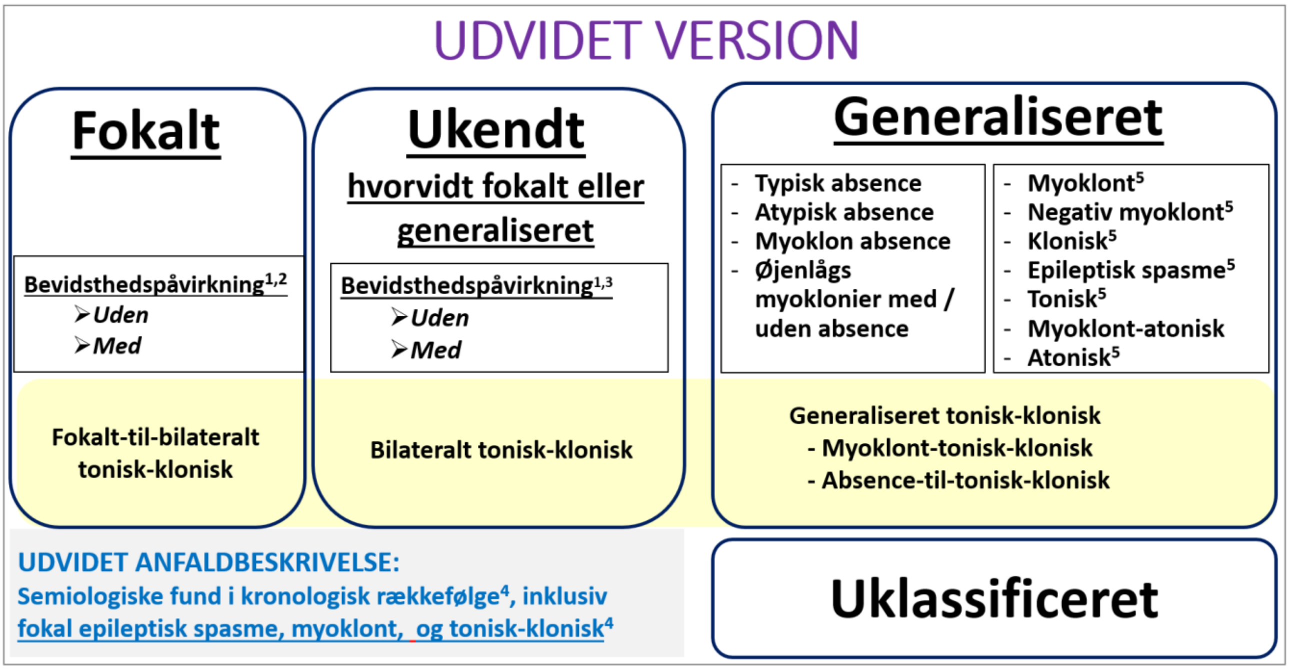

1. Bevidsthedspåvirkning er operationelt defineret ved manglende evne til at gengive begivenheder (amnesi) og ved manglende responsitivitet.
2. Når bevidsthedspåvirkningen er ukendt, klassificeres anfaldet som fokalt (uden at specificere yderligere).
3. Hvis bevidsthedspåvirkningen er ukendt, klassificeres anfaldet som ukendt hvorvidt det er fokalt eller generaliseret (uden at specificere yderligere).
4. Beskrevet ved brug af terminologi fra ILAE's "Semiology glossary" (se Tabel 2).
5. Disse fænomener kan også forekomme ved fokale anfald (unilateralt eller asymmetrisk) som en del af semiologien for et fokalt anfald.

Anfaldstyper vises i sort, mens beskrivelse af anfald vises i blåt. Den horisontale gule baggrund i figurerne understreger, at bilaterale tonisk-kloniske anfald er forbundet med høj sygelighed og dødelighed. Denne anfaldstype kan forekomme i alle tre hovedanfaldsklasser.

French translation

1. Focale (F)

1.1. Crise avec Conscience Préservée (CCP)
1.2. Crise avec Altération de la Conscience (CAC)
1.3. Crise Focale avec évolution Tonico-Clonique Bilatérale (CFTCB)

Descripteurs

- *Basiques:*
  - *Avec manifestations observables*
  - *Sans manifestation observable*
- *Etendus:*
  - *Descripteurs de séméiologie dans l'ordre chronologique: Sémiologie (glossaire*) + Modificateurs somatotopiques*

2. Inconnu si focale ou generalisée (U)

2.1. Inconnu si focale ou généralisée - Crise avec Conscience Préservée (CCP)
2.2. Inconnu si focale ou généralisée - Crise avec Altération de la Conscience (CAC)
2.3. Inconnu si focale ou généralisée – Crise Tonico-Clonique Bilatérale (CTCB)

Descripteurs

- *Basiques:*
  - *Avec manifestations observables*
  - *Sans manifestation observable*
- *Etendus:*
  - *Descripteurs de séméiologie dans l'ordre chronologique: Sémiologie (glossaire*) + Modificateurs somatotopiques*

3. Generalisée (G)

3.1. Absences (AS)
- 3.1.1. Absence Typique (AT)
- 3.1.2. Absence Atypique (AA)
- 3.1.3. Absence Myoclonique (AM)
- 3.1.4. Myoclonies des Paupières avec / sans Absence (MPA)

3.2. Crise Généralisée Tonico-Clonique (CGTC)
- 3.2.1. Crise Myoclono-Tonico-Clonique
- 3.2.2. Absence avec évolution Tonico-Clonique

3.3. Autres crises generalisées**
- 3.3.1. Crise Généralisée Myoclonique (CGM)
- 3.3.2. Crise Généralisée Clonique (CGC)
- 3.3.3. Crise Généralisée Myoclonique Negative (CGMN)
- 3.3.4. Spasme Epileptique Généralisé (SEG)
- 3.3.5. Crise Généralisée Tonique (CGT)
- 3.3.6. Crise Généralisée Atonique (CGA)
- 3.3.7. Crise Généralisée Myoclono-Atonique (CGMA)

4. Inclassable

*Voir Table 2 avec les caractéristiques sémiologiques
** Il s'agit d'un terme de regroupement et non d'un concept défini.

VERSION DE BASE

Focale

Conscience[1,2]
- ***Préservée***
- ***Altérée***

Crise Focale avec évolution Tonico-Clonique Bilatérale

Inconnu
si focale ou generalisée

Conscience[1,3]
- ***Préservée***
- ***Altérée***

Crise tonico-clonique bilatérale

Generalisée

Absence

Autre crise généralisée

Crise Généralisée Tonico-Clonique

Descripteurs de base:
Avec ou sans manifestations observables[4]

Inclassable

Légendes de la Figure 1:

1. *Défini de manière opérationnelle par la conscience et la réactivité.*
2. *Lorsque l'état de conscience est non connu, classé comme focale (sans préciser la sous-classification).*
3. *Lorsque l'état de conscience est non connu, classé comme inconnu si focale ou généralisée (sans préciser la sous-classification).*
4. *Les manifestations observables sont facilement reconnues par un témoin oculaire. Celles-ci peuvent être motrices, aphasiques, autonomiques ou autres (voir table 2). Une altération de la conscience est considérée comme une manifestation observable.*

Les classificateurs (types de crise) sont indiqués en noir, tandis que les descripteurs sont en bleu. Le fond jaune horizontal souligne que les crises tonico-cloniques bilatérales - associées à la morbidité et à la mortalité les plus élevées - peuvent survenir dans les trois principaux types de crise.

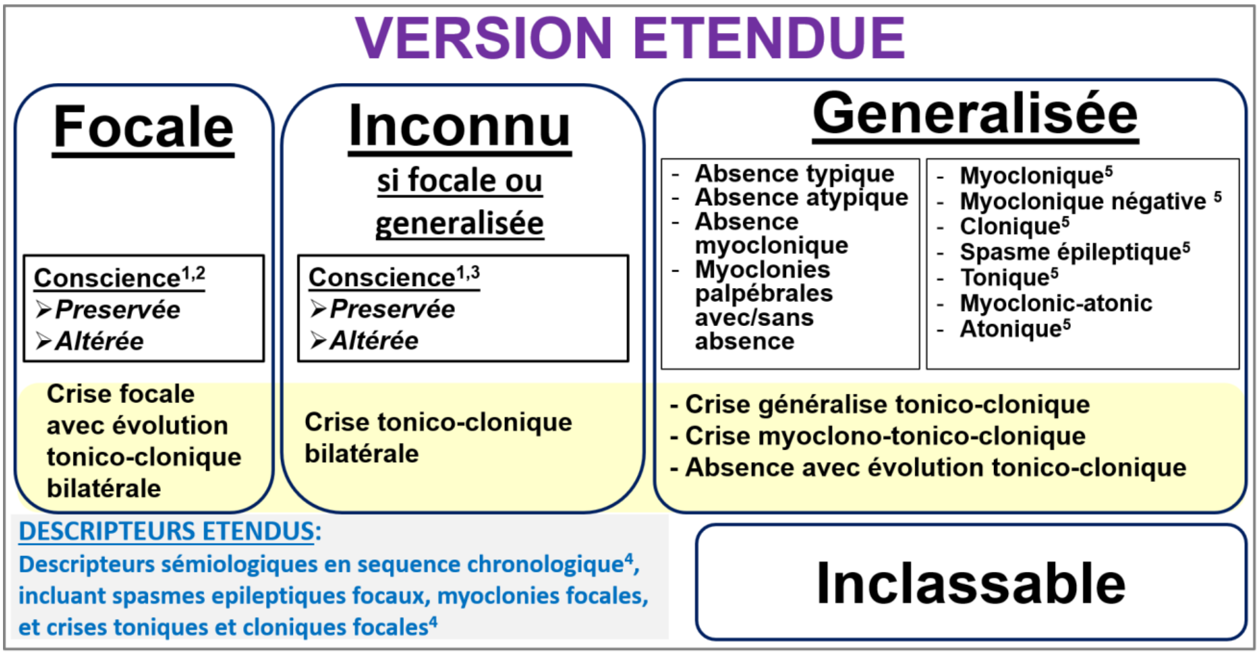

Légendes de la Figure 2:

1. *Défini de manière opérationnelle par la conscience et la réactivité.*
2. *Lorsque l'état de conscience est non connu, classé comme focale (sans préciser la sous-classification).*
3. *Lorsque l'état de conscience est non connu, classé comme inconnu si focale ou généralisée (sans préciser la sous-classification).*
4. *Décrit à l'aide des termes du glossaire de sémiologie de l'ILAE (voir table 2).*
5. *Ces phénomènes peuvent également se produire dans les crises focales (généralement de manière unilatérale ou asymétrique) comme un partie de la sémiologie d'une crise focale.*

Les classificateurs (types de crise) sont indiqués en noir, tandis que les descripteurs sont en bleu. Le fond jaune horizontal souligne que les crises tonico-cloniques bilatérales - associées à la morbidité et à la mortalité les plus élevées - peuvent survenir dans les trois principaux types de crise.

German Translation

1. Fokal (F)

1.1. Fokaler Anfall mit erhaltenem Bewußtsein
1.2. Fokaler Anfall mit gestörtem Bewußtsein
1.3. Fokal-zu-bilateral tonisch-klonischer Anfall

Deskriptoren

- *Grundlegend:*
  - *Mit beobachtbaren Manifestationen*
  - *Ohne beobachtbare Manifestationen*
- *Erweitert:*
  - *Semiologie Deskriptoren in chronologischer Sequenz: Semiologie (Glossar*) + Somatotopische Modifikatoren*

2. Unbekannt ob fokal oder generalisiert (U)

2.1. Unbekannt ob fokal oder generalisiert - Anfall mit erhaltenem Bewußtsein
2.2. Unbekannt ob fokal oder generalisiert - Anfall mit gestörtem Bewußtsein
2.3. Unbekannt ob fokal oder generalisiert - Bilateral tonisch-klonischer Anfall

Deskriptoren

- *Grundlegend:*
  - *Mit beobachtbaren Manifestationen*
  - *Ohne beobachtbare Manifestationen*
- *Erweitert:*
  - *Semiologie Deskriptoren in chronologischer Sequenz: Semiologie (Glossar*) + Somatotopische Modifikatoren*

3. Generalisiert

3.1. Absence Anfälle
- 3.1.1. Typischer Absence Anfall
- 3.1.2. Atypischer Absence Anfall
- 3.1.3. Myoklonischer Absence Anfall
- 3.1.4. Augenlid Myoklonien mit/ohne Absence

3.2. Generalisiert tonisch-klonischer Anfall
- 3.2.1. Myoklonisch tonisch-klonischer Anfall
- 3.2.2. Absence-zu-tonisch-klonischem Anfall

3.3. Andere generalisierte Anfälle**
- 3.3.1. Generalisiert myoklonischer Anfall
- 3.3.2. Generalisiert klonischer Anfall
- 3.3.3. Generalisiert negative myoklonischer Afall
- 3.3.4. Generalisiert epileptischer Spasmus
- 3.3.5. Generalisiert tonischer Anfall
- 3.3.6. Generalisiert atonischer Anfall
- 3.3.7. Generalisiert myoklonisch-atonischer Anfall

4. Unklassifiziert

*Siehe Tabelle 2 mit den Semiologie Merkmalen.
**Dies ist ein Gruppierungsbegriff, kein definiertes Konzept.

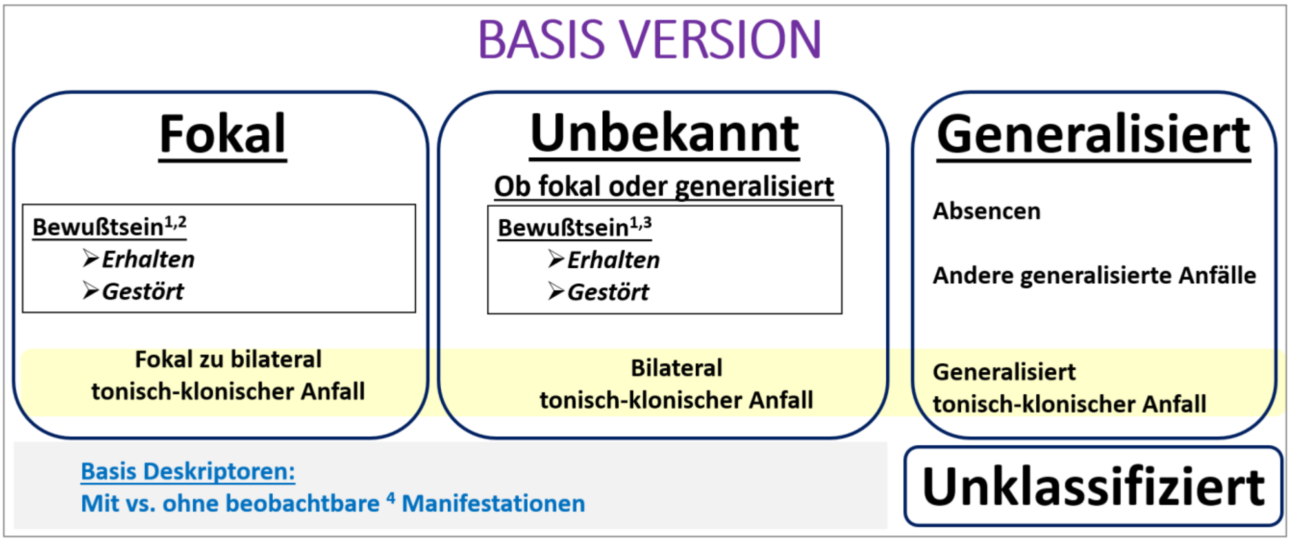

1. Operational definiert als Erinnerungsfähigkeit und Reaktionsfähigkeit.
2.Falls der Bewußtseinszustand unbekannt ist, klassifiziere als fokal (ohne Spezifizierung der Subklassifikation)
3. Falls der Bewußtseinszustand unbekannt ist , klassifiziere als unbekannt ob fokal oder generalisiert (ohne Spezifizierung der Subklassifikation)
4. Beobachtbare Manifestationen werden einfach durch Augenzeugen erfasst. Dies kann motorische,aphasische,autonome oder andere Symptome betreffen (siehe Tabelle 2).Gestörtes Bewußtsein qualifiziert eine beobachtbare Manifestation.

Klassifikatoren (Anfallstypen) sind schwarz , Deskriptoren in blau markiert.Der horizontale gelbe Hintergrund in der Abbildung hebt hervor,daß bilateral tonisch- klonische Anfälle-assoziiert mit höchster Morbidität und Mortalität- in allen drei Hauptklassen auftreten.

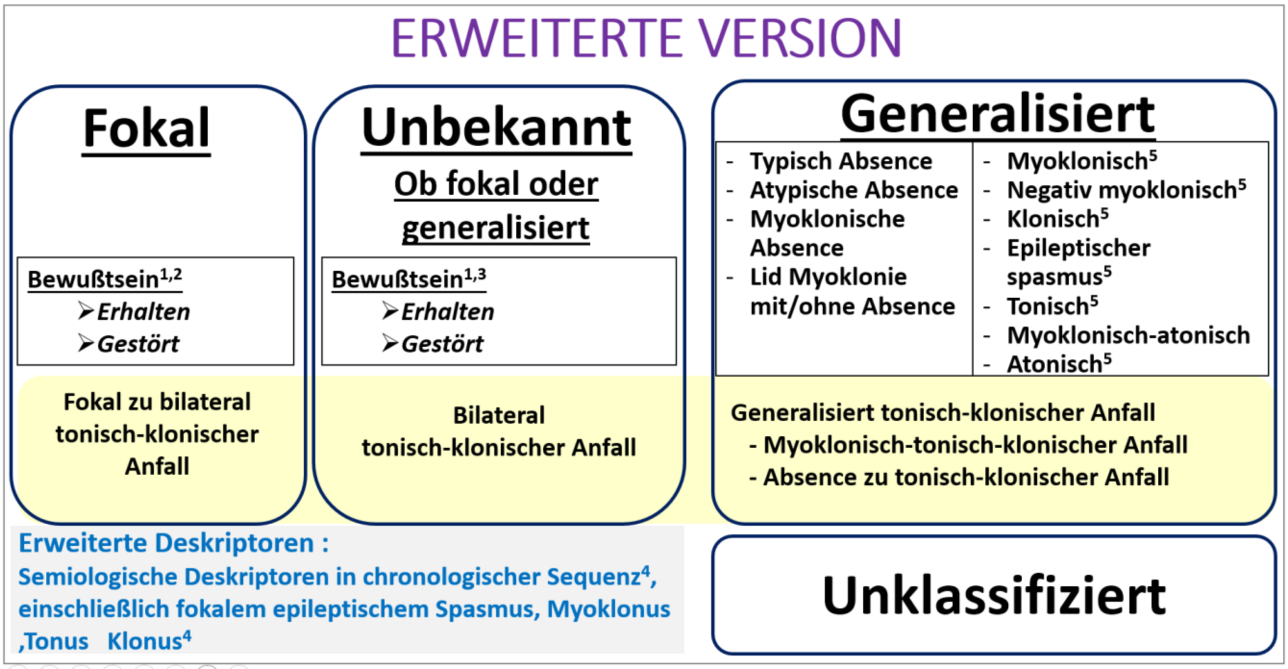

1. Operational definiert als Erinnerungsfähigkeit und Reaktionsfägigkeit.
2. Falls der Bewußtseinszustand unbekannt ist, klassifiziere als fokal (ohne Spezifizierung der Subklassifikation)
3. Falls der Bewußtseinszustand unbekannt ist , klassifiziere als unbekannt ob fokal oder generalisiert (ohne Spezifizierung der Subklassifikation)
4. Beschrieben durch Verwendung des ILAE Semiologie Glossars (siehe Tabelle2).
5. Diese Phänomene können auch bei fokalen Anfällen als Teil der Semiologie eines fokalen Anfalles (gewöhnlich unilateral oder asymmetisch) auftreten.

   Klassifikatoren (Anfallstypen) sind schwarz , Deskriptoren in blau markiert.Der horizontale gelbe Hintergrund in der Abbildung hebt hervor,daß bilateral tonisch- klonische Anfälle-assoziiert mit höchster Morbidität und Mortalität- in allen drei Hauptklassen auftreten.

Hungarian translation

1. Fokális (F)

1.1. Fokális, Megtartott Tudattal járó roham (FMTR)
1.2. Fokális, Tudatzavarral járó roham (FTR)
1.3. Fokálisból bilaterális tónusos-klónusos roham (FBTKR)

Leírók

- *Alap:*
  - *Megfigyelhető tünetekkel*
  - *Megfigyelhető tünetek nélkül*
- *Kiterjesztett:*
  - *Szemiológiai leírók kronológiai sorrendben: Szemiológia (szójegyzék*) + Szomatotóp módosítók*

2. Ismeretlen, hogy fokális vagy generalizált (I)

2.1. Ismeretlen, hogy fokális vagy generalizált eredetű - Megtartott Tudattal járó roham (IMTR)
2.2. Ismeretlen, hogy fokális vagy generalizált eredetű - Tudatzavarral járó roham (ITR)
2.3. Ismeretlen, hogy fokális vagy generalizált eredetű – bilaterális tónusos-klónusos roham (IBTKR)

Leírók

- *Alap:*
  - *Megfigyelhető tünetekkel*
  - *Megfigyelhető tünetek nélkül*
- *Kiterjesztett:*
  - *Szemiológiai leírók kronológiai sorrendben: Szemiológia (szójegyzék*) + Szomatotóp módosítók*

3. Generalizált (G)

3.1. Absence roham (AR)
- 3.1.1. Típusos absence roham (TA)
- 3.1.2. Atípusos absence roham (AA)
- 3.1.3. Mioklónusos absence roham (MA)
- 3.1.4. Szemhéj mioklónia absence-szal vagy absence nélkül (SzMA)

3.2. Generalizált tónusos-klónusos roham (GTKR)
- 3.2.1. Mioklónusos-tónusos-klónusos roham
- 3.2.2. Absence indulású tónusos-klónusos roham

3.3. Egyéb generalizált rohamok**
- 3.3.1. Generalizált mioklónusos roham (GMR)
- 3.3.2. Generalizált klónusos roham (GKR)
- 3.3.3. Generalizált negatív mioklónusos roham (GNMR)
- 3.3.4. Generalizált epilepsziás spazmus (GES)
- 3.3.5. Generalizált tónusos roham (GTR)
- 3.3.6. Generalizált atónusos roham (GAR)
- 3.3.7. Generalizált mioklónusos-atónusos roham (GMAR)

4. **Osztályozatlan**

*Ld. 2. táblázat szemiológiai jelenségekkel
**Ez egy csoportosító kifejezés, nem egy meghatározott fogalom

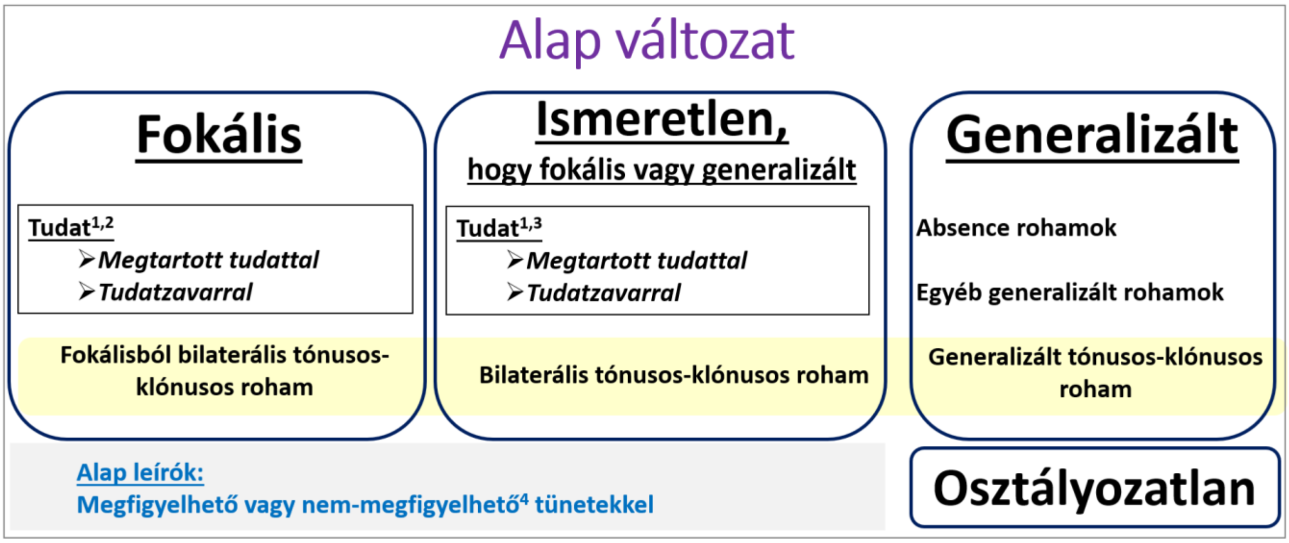

1. Ábraaláírás

1. A tudatállapot az éberség és a válaszkészség alapján értékelhető.
2. Ha a tudatállapot ismeretlen, "fokális" osztályozás adható (a szub-klasszifikáció pontosítása nélkül)
3. Ha a tudatállapot ismeretlen, "Ismeretlen, hogy fokális vagy generalizált" osztályozás adható (a szub-klasszifikáció pontosítása nélkül)
4. A megfigyelhető tünetek szemtanúk által könnyen felismerhetők. Ezek lehetnek motoros, afáziás, autonóm vagy egyéb (ld. 2. táblázat) tünetek. A tudatzavar megfigyelhető tünetnek minősül.

Az osztályozók (rohamtípusok) feketével olvashatók, a leírók kék színnel. A vízszintes sárga háttér a bilaterális tónusos-klónusos rohamokat emeli ki – melyek a legmagasabb morbiditással és mortalitással függenek össze, és mindhárom fő rohamosztályban előfordulhatnak.

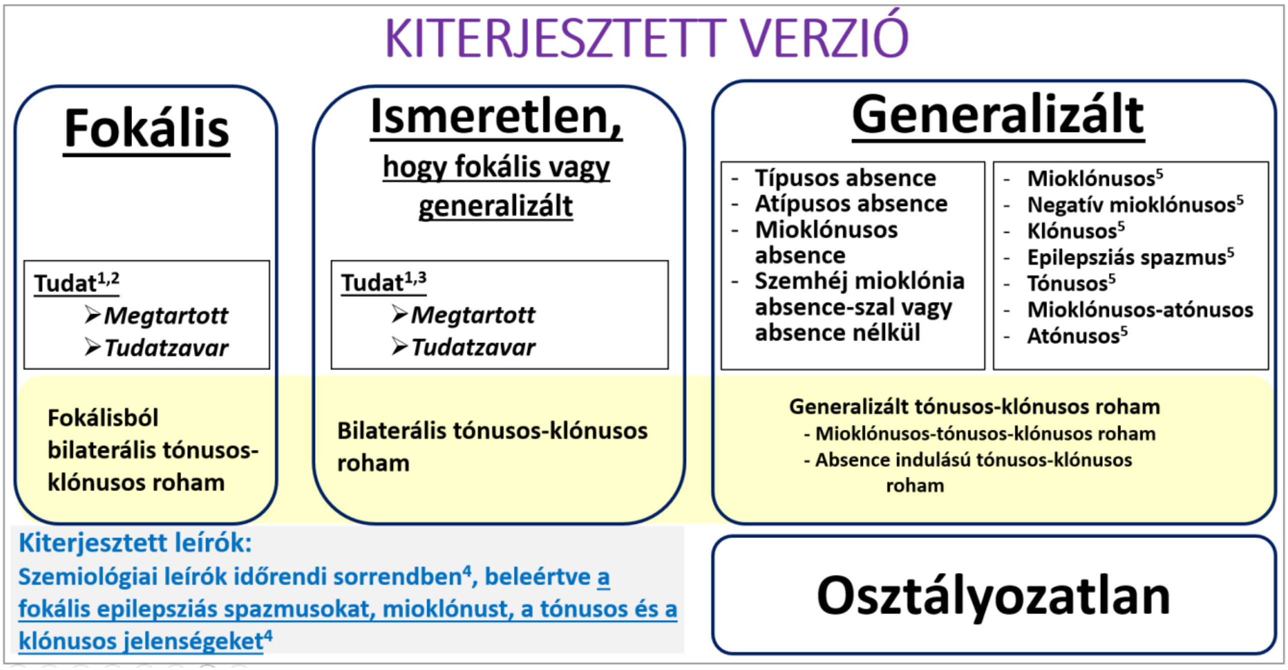

2. ábraaláírás

1. A tudatállapot az éberség és a válaszkészség alapján értékelhető.
2. Ha a tudatállapot ismeretlen, "fokális" osztályozás adható (a szub-klasszifikáció pontosítása nélkül)
3. Ha a tudatállapot ismeretlen, "Ismeretlen, hogy fokális vagy generalizált" osztályozás adható (a szub-klasszifikáció pontosítása nélkül)
4. Az ILAE szemiológiai szójegyzéke alapján (ld. 2. táblázat)
5. Ezek a rohamjelenségek fokális rohamokban is előfordulhatnak (általában unilaterálisan vagy aszimmetrikusan) egy fokális rohamszemiológia részjelenségeként.

Az osztályozók (rohamtípusok) feketével olvashatók, a leírók kék színnel. A vízszintes sárga háttér a bilaterális tónusos-klónusos rohamokat emeli ki – melyek a legmagasabb morbiditással és mortalitással függnek össze, és mindhárom fő rohamosztályban előfordulhatnak.

Italian translation

9. Crisi Focale (F)

9.1. Crisi Focale con Coscienza Preservata (Focal Preserved Consciousness seizure FPC)
9.2. Crisi Focale con Coscienza Compromessa (Focal Impaired Consciousness seizure FIC)
9.3. Crisi da Focale a tonico-clonica bilaterale (Focal-to-bilateral tonic-clonic seizure FBTC)

Descrittori

- *Base:*
  - Con segni e/o sintomi obiettivabili
  - Senza segni e/o sintomi obiettivabili
- *Ampliata:*
  - *Descrittori della semiologia in sequenza cronologica: Semiologia (glossario*) + Modificatori somatotopici*

10. Crisi Non valutabile (sconosciuta) se focale o generalizzata

10.1. Non valutabile (sconosciuta) se focale o generalizzata – Crisi a Coscienza Preservata (Unknown whether focal or generalized - Preserved Consciousness seizure PC)
10.2. Non valutabile (sconosciuta) se focale o generalizzata – Crisi a coscienza compromessa (Unknown whether focal or generalized - Impaired Consciousness seizure IC)
10.3. Non valutabile (sconosciuta) se focale o generalizzata – Crisi con stato di coscienza non valutabile (sconosciuto) (Unknown whether focal or generalized - Bilateral tonic-clonic seizure BTC)

Descrittori

- *Base:*
  - Con segni e/o sintomi obiettivabili
  - Senza segni e/o sintomi obiettivabili
- *Ampliata:*
  - *Descrittori della semiologia in sequenza cronologica: Semiologia (glossario*) + Modificatori somatotopici*

11. Crisi Generalizzata (G)

11.1. Assenze
11.1.1. Assenza tipica (Typical absence seizure (TA)
11.1.2. Assenza atipica (Atypical absence seizure (AA)
11.1.3. Assenza mioclonica (Myoclonic absence seizure (MA)
11.1.4. Mioclonie palpebrali con/senza assenza (Eyelid myoclonia with / without absence (EMA)
11.2. Crisi tonico-cloniche generalizzate (Generalized tonic-clonic seizure (GTC)
11.2.1. Mioclono tonico-cloniche Myoclonic tonic-clonic seizure
11.2.2. Assenza seguita da crisi tonico-clonica (Absence-to-tonic-clonic seizure
11.3. Altre crisi generalizzate **
11.3.1. Crisi miocloniche generalizzate (Generalized myoclonic seizure (GM)
11.3.2. Crisi cloniche generalizzate (Generalized clonic seizure (GC)
11.3.3. Mioclono negativo generalizzato (Generalized negative myoclonic seizure (GNM)
11.3.4. Spasmi epilettici generalizzati (Generalized epileptic spasm (GES)
11.3.5. Crisi toniche generalizzate (Generalized tonic seizure (GT)
11.3.6. Crisi atoniche generalizzate (Generalized atonic seizure (GA)
11.3.7. Crisi mioclono-atoniche generalizzate (Generalized myoclonic-atonic seizure (GMA)

12. **Crisi non classificabile**

*Vedi Tabella 2 con le caratteristiche della semiologia.
**Questa definizione identifica un gruppo non un concetto.

VERSIONE BASE

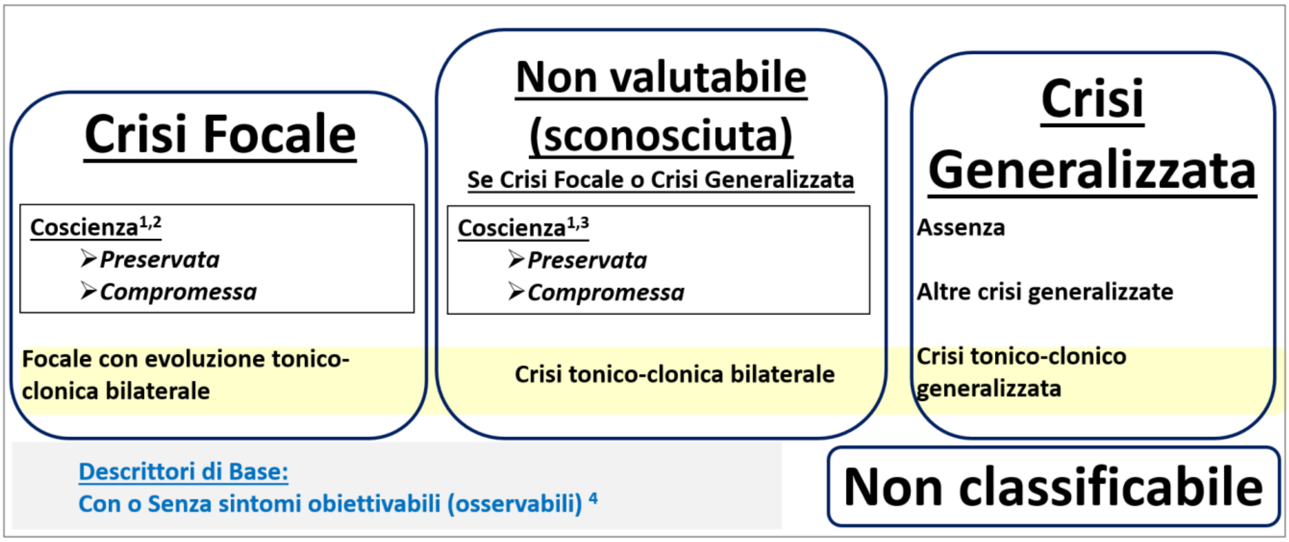

Legenda della Figura 1

1. Operativamente definita da consapevolezza e responsitivà
2. Quando lo stato di coscienza e' sconosciuto, classificare come crisi focale (senza specificare la sotto classificazione)
3. Se lo stato di coscienza e' sconosciuto, classificare come non valutabile (sconosciuto) se crisi focale o crisi generalizzata (senza specificare la sotto classificazione)
4. I sintomi obiettivabili (osservabili) sono prontamente riconoscibili da un testimone. Questi possono essere motori, afasici, autonomici o altro (vedi Tabella 2). La coscienza compromessa e' considerata un sintomo obiettivabile. Questi potrebbero essere motori, verbali, autonomici, o altro (vedi tabella della semeiologia).

La compromissione della consapevolezza è considerata un segno obiettivabile.
Le definizioni (tipo di crisi) sono indicate in nero, mentre i descrittori sono indicati in blu. Lo sfondo giallo nella figura sottolinea che le crisi bilaterali tonico-cloniche – che sono associate con il piu' alto rischio di morbidita' e mortalita' – possono verificarsi in tutte le classi di crisi.

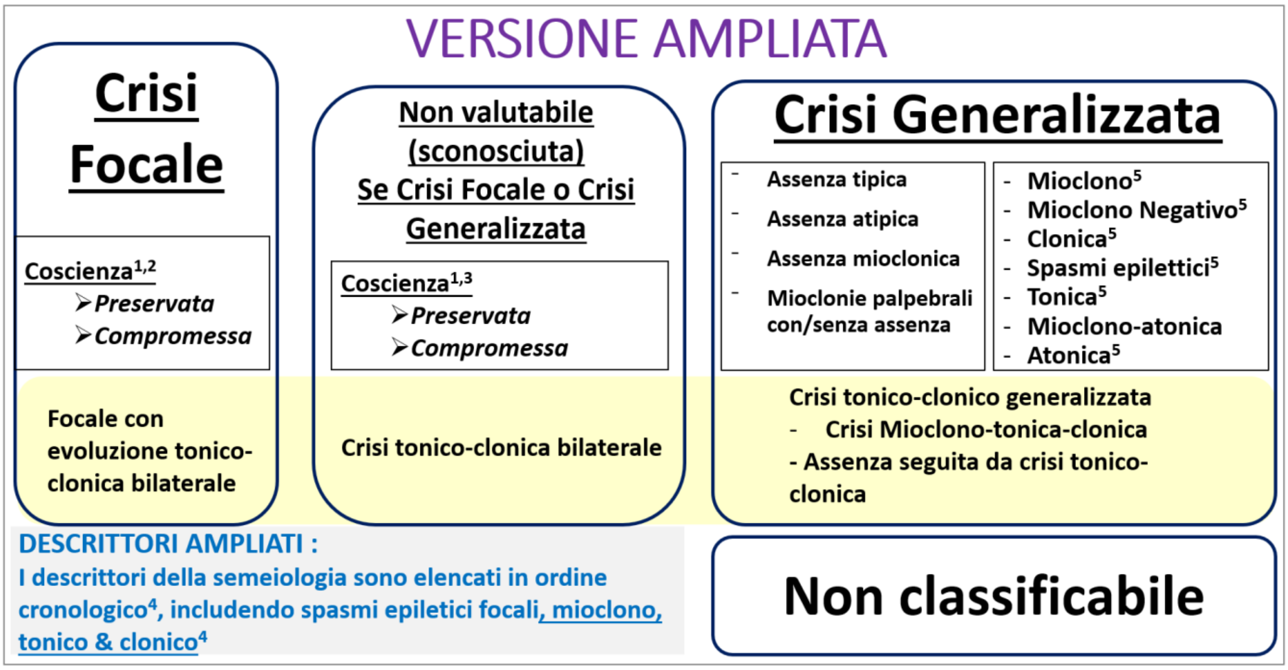

Legenda della Figura 2

1. Operativamente determinata da consapevolezza e responsività
2. Quando lo stato di coscienza e' sconosciuto, classificare come crisi focale (senza specificare la sotto classificazione)
3. Se lo stato di coscienza e' sconosciuto, classificare come non valutabile (sconosciuto) se crisi focale o crisi generalizzata (senza specificare la sotto classificazione)
4. Descritti utilizzando I termini contenuti nel glossario della semeiologia ILAE (vedi tabella 2).
5. Questi fenomeni possono presentarsi anche nelle crisi focali (generalmente unilaterali o asimmetrici) come parte integrante della semiologia di una crisi focale.

Le definizioni (tipo di crisi) sono indicate in nero, mentre i descrittori sono indicati in blu. Lo sfondo giallo nella figura sottolinea che le crisi bilaterali tonico-cloniche – che sono associate con il piu' alto rischio di morbidita' e mortalita' – possono verificarsi in tutte le classi di crisi.

Japanese translation

1. 焦点発作 **Focal (F)**
   - 1.1. 焦点意識保持発作 Focal preserved consciousness seizure (FPC)
   - 1.2. 焦点意識減損発作 Focal impaired consciousness seizure (FIC)
   - 1.3. 点起始両側強直間代発作 Focal-to-bilateral tonic-clonic seizure (FBTC)

   記述子***Descriptors***
   - 基本版*Basic:*
     - 観察可能な症状を伴う*With observable manifestations*
     - 観察可能な症状を伴わない*Without observable manifestations*
   - 拡張版*Expanded:*
     - 時系列での症候の記述*Semiology descriptors in chronological sequence:*
       症候（用語集*） + 体部位に関する修飾語*Semiology (glossary*) + Somatotopic modifiers*

2. 焦点／全般不明発作 **Unknown whether focal or generalized (U)**
   - 2.1. 焦点／全般不明—意識保持発作 Unknown whether focal or generalized - preserved consciousness seizure (PC)
   - 2.2. 焦点／全般不明—意識減損発作 Unknown whether focal or generalized - impaired consciousness seizure (IC)
   - 2.3. 焦点／全般不明—両側強直間代発作 Unknown whether focal or generalized - Bilatelral tonic-clonic seizure (BTC)

   記述子***Descriptors***
   - 基本版*Basic:*
     - 観察可能な症状を伴う*With observable manifestations*
     - 観察可能な症状を伴わない*Without observable manifestations*
   - 拡張版*Expanded:*
     - 時系列での症候の記述*Semiology descriptors in chronological sequence:*
       症候（用語集*） + 体部位に関する修飾語*Semiology (glossary*) + Somatotopic modifiers*

3. 全般発作 **Generalized (G)**
   - 3.1. 欠神発作 Absence seizures (AS)
     - 3.1.1. 定型欠神発作 Typical absence seizure (TA)
     - 3.1.2. 非定型欠神発作 Atypical absence seizure (AA)
     - 3.1.3. ミオクロニー欠神発作 Myoclonic absence seizure (MA)
     - 3.1.4. 欠神を伴う／伴わない眼瞼ミオクロニー Eyelid myoclonia with / without absence (EMA)
   - 3.2. 全般強直間代発作 Generalized tonic-clonic seizure (GTC)
     - 3.2.1. ミオクロニー強直間代発作 Myoclonic tonic-clonic seizure
     - 3.2.2. 欠神強直間代発作 Absence-to-tonic-clonic seizure
   - 3.3. その他の全般発作** Other generalized seizures**
     - 3.3.1. 全般ミオクロニー発作 Generalized myoclonic seizure (GM)
     - 3.3.2. 全般間代発作Generalized clonic seizure (GC)
     - 3.3.3. 全般陰性ミオクロニー発作 Generalized negative myoclonic seizure (GNM)
     - 3.3.4. 全般てんかん性スパズム Generalized epileptic spasmsd (GES)
     - 3.3.5. 全般強直発作 Generalized tonic seizure (GT)
     - 3.3.6. 全般脱力発作 Generalized atonic seizure (GA)
     - 3.3.7. 全般ミオクロニー脱力発作 Generalized myoclonic-atonic seizure (GMA)

4. 分類不能発作 **Unclassified**

*症候に関して表2を参照
**グループ分けのための用語であり、定義された概念ではない。

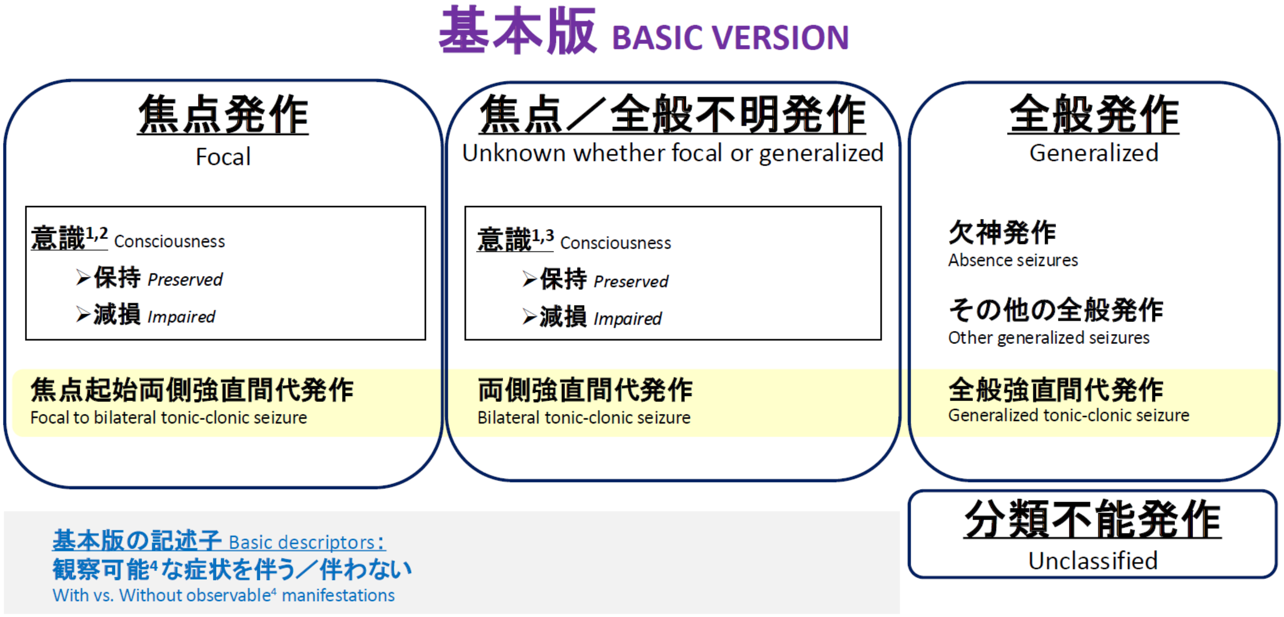

図1の説明文：

1. 意識性（awareness）と反応性（responsiveness）によって操作的に定義される。
2. 意識状態が不明の場合、焦点発作へ分類（下位分類を特定せず）
3. 意識状態が不明の場合、焦点／全般不明発作へ分類（下位分類を特定せず）
4. 観察可能な症状とは目撃者によって容易に認識されるものである。これらは運動、失語、自律神経、あるいはその他の症状の可能性がある（表2を参照）。意識減損は観察可能な症状の一つとみなす。

分類子（発作型）は黒字で、記述子は青字で示す。図中の水平方向に示した黄色背景は両側強直間代発作（罹患率・死亡率が最も高い）が3つの主発作分類全てにおいて生じうることを強調したものである。

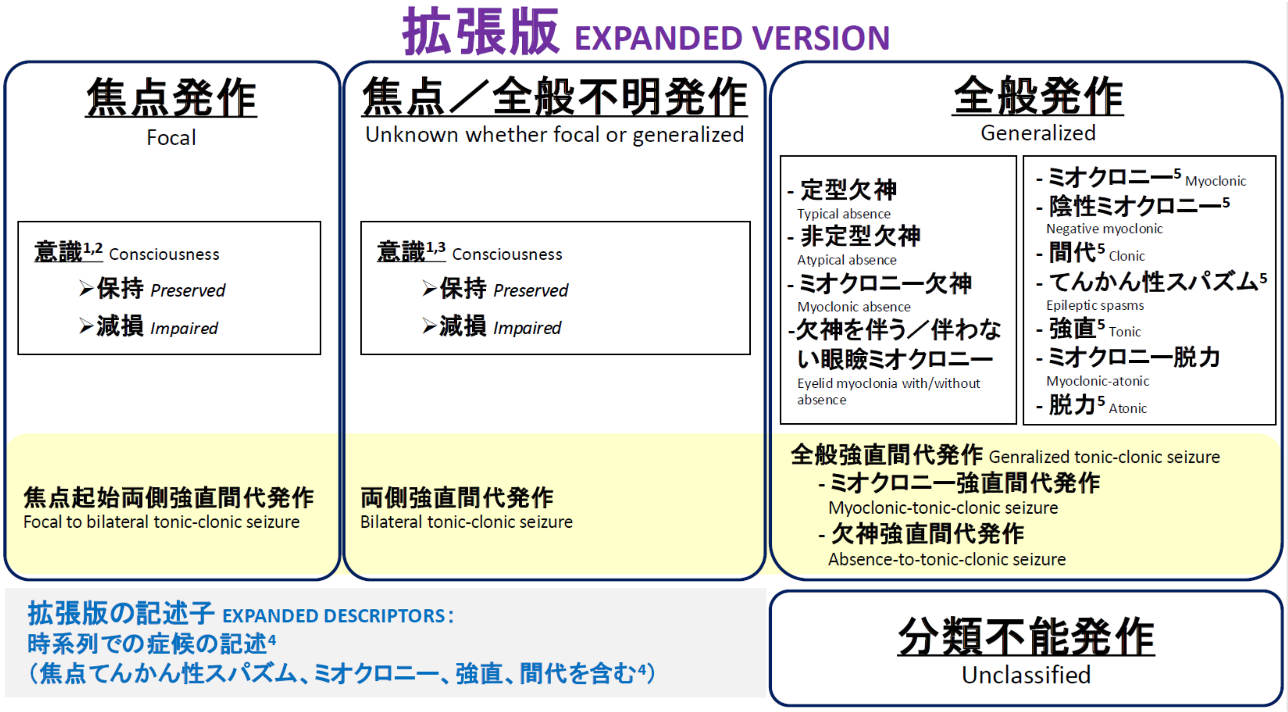

図2の説明文：

1. 意識性（awareness）と反応性（responsiveness）によって操作的に定義される。
2. 意識状態が不明の場合、焦点発作へ分類（下位分類を特定せず）
3. 意識状態が不明の場合、焦点／全般不明発作へ分類（下位分類を特定せず）
4. ILAEの症候学用語集（ILAE semiology glossary）に記載される用語を用いて記述する。
5. これらの現象は、焦点発作の症候の一部として（通常は片側性あるいは非対称性に）焦点発作でも生ずることがある。

分類子（発作型）は黒字で、記述子は青字で示す。図中の水平方向に示した黄色背景は両側強直間代発作（罹患率・死亡率が最も高い）が3つの主発作分類全てにおいて生じうることを強調したものである。

Korean translation

1. 국소

1.1. 국소 의식보존 발작

1.2. 국소 의식소실 발작

1.3. 양측 강직-간대 이행 발작

추가기술

- *기본:*
  - *동반 관찰소견들*
  - *무동반 관찰소견들*
- *확장:*
  - *시간 순서로 증세학 기술:*
    증세학 (용어집) + 신체 부위 수식어*

2. 불명

2.1. 불명 – 의식보존 발작

2.2. 불명 – 의식소실 발작

2.3. 불명 – 양측 강직-간대 발작

추가기술

- *기본:*
  - *동반 관찰소견들*
  - *무동반 관찰소견들*
- *확장:*
  - *시간 순서로 증세학 기술:*
    증세학 (용어집) + 신체 부위 수식어*

3. 전체

3.1. 소발작

3.1.1. 전형 소발작

3.1.2. 비전형 소발작

3.1.3. 근간대 소발작

3.1.4. 안검 근간대 발작

3.2. 전체 강직-간대 발작

3.2.1. 근간대 강직-간대 발작

3.2.2. 소-강직-간대 발작

3.3. 기타 전체 발작**

3.3.1. 전체 근간대 발작

3.3.2. 전체 간대 발작

3.3.3. 전체 음성 간대 발작

3.3.4. 전체 뇌전증 연축

3.3.5. 전체 강직 발작

3.3.6. 전체 무긴장 발작

3.3.7. 전체 근간대-무긴장 발작

*증세학에 대한 표2 참고

**이것은 명확히 정의된 개념이 아니라, 분류를 위한 용어임.

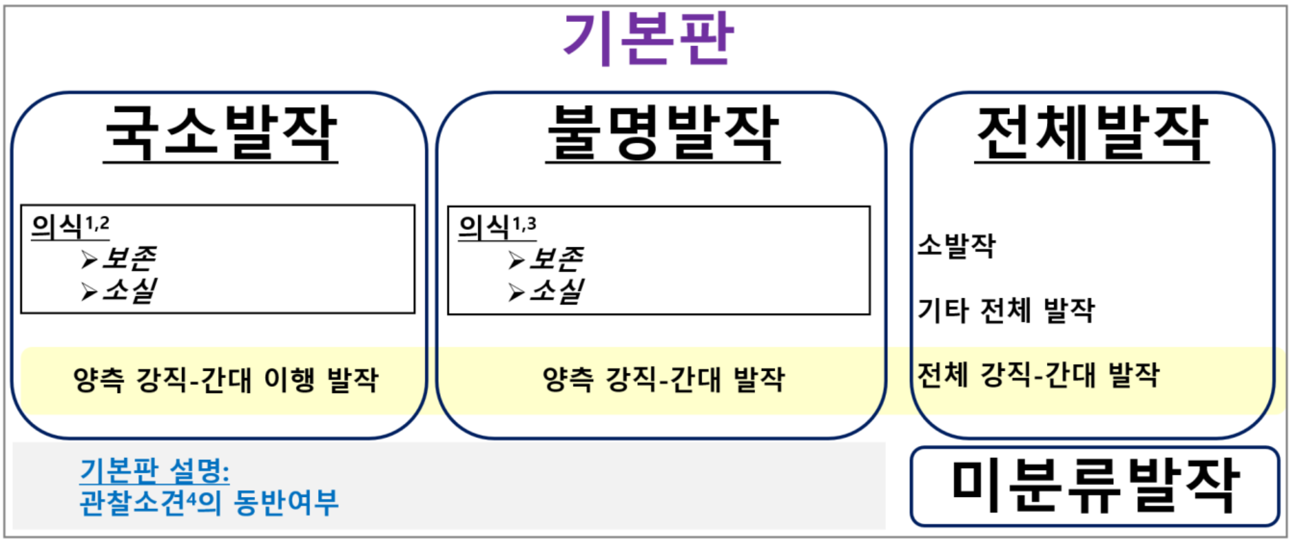

1. 지각과 반응으로 정의함.
2. 의식 상태를 모를 때는, 국소발작으로 분류 (세부분류 없이)
3. 의식 상태를 모를 때는, 불명발작으로 분류 (세부분류 없이)
4. 관찰소견은 목격자가 쉽게 알아챌 수 있는 증상임. 운동, 실어증, 자율신경계 또는 기타증상(표2 참고)일 수 있음. 의식장애는 관찰소견에 해당함.

발작분류는 검정색, 추가기술은 파란색으로 표기함. 그림에서 가로로 된 노란색 배경은 가장 높은 이환율과 사망률과 관련된 양측 강직-간대 발작이 세가지 주요 발작 분류 모두에서 발생할 수 있음을 강조함.

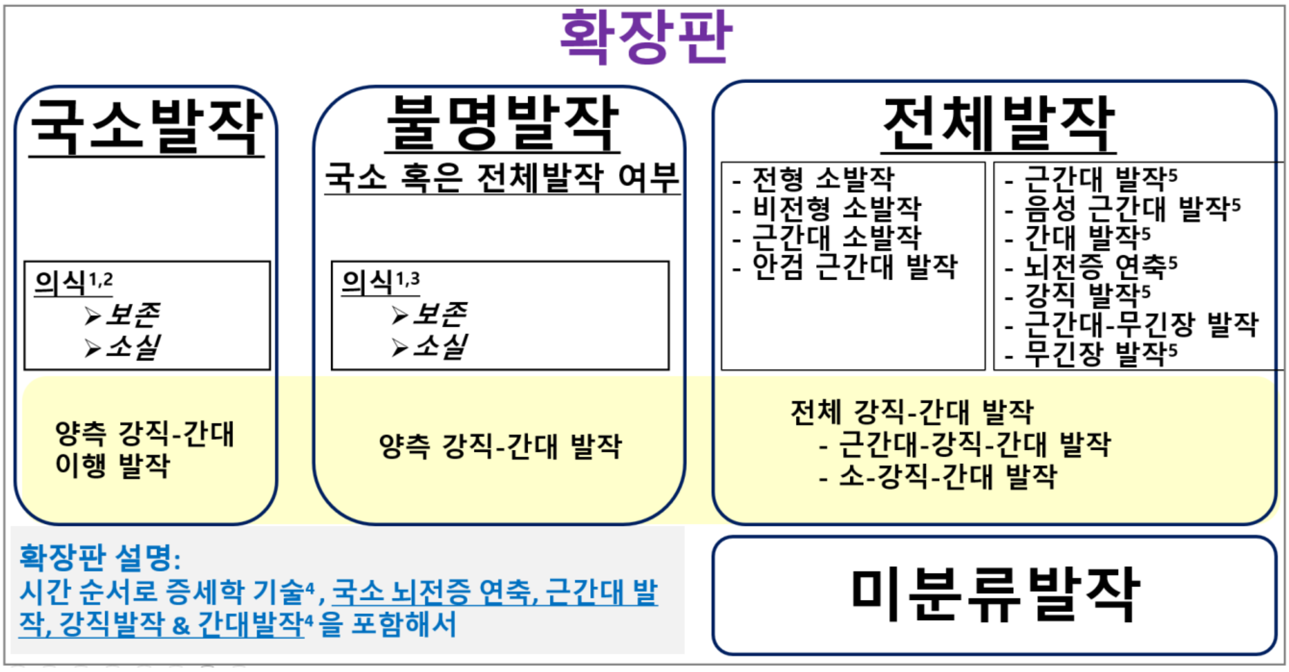

1. 지각과 반응으로 정의함.
2. 의식 상태를 모를 때는, 국소발작으로 분류함 (세부분류 없이).
3. 의식 상태를 모를 때는, 불명발작으로 분류함 (세부분류 없이).
4. ILAE 증세학 용어집의 용어를 사용하여 설명함 (표2 참고).
5. 이러한 현상들은 국소발작(보통 편측 혹은 비대칭적)에서도 국소발작의 증세학의 일부로 발생할 수 있음.

발작분류는 검정색, 추가기술은 파란색으로 표기함. 그림에서 가로로 된 노란색 배경은 가장 높은 이환율과 사망률과 관련된 양측 강직-간대 발작이 세가지 주요 발작 분류 모두에서 발생할 수 있음을 강조함.

Portuguese translation (Brazilian)

1. Focal (F)

1.1. Crise focal com Consciência Preservada (FCP)
1.2. Crise focal com Comprometimento da Consciência (FCC)
1.3. Crise focal para tônico-clônica bilateral (FTCB)

Descritores

- *Básico:*
  - *Com manifestações observáveis*
  - *Sem manifestações observáveis*
- *Expandido:*
  - *Descritores semiológicos em sequência cronológica: Semiologia (glossário*) + Modificadores somatotópicos*

2. Desconhecida se focal ou generalizada (D)

2.1. Desconhecida se focal ou generalizada – Crise com consciência preservada (CP)
2.2. Desconhecida se focal ou generalizada - Crise com consciência comprometida (CC)
2.3. Desconhecida se focal ou generalizada – Crise tônico-clônica bilateral (TCB)

Descritores

- *Básico:*
  - *Com manifestações observáveis*
  - *Sem manifestações observáveis*
- *Expandido:*
  - *Descritores semiológicos em sequência cronológica: Semiologia (glossário*) + Modificadores somatotópicos*

3. Generalizada (G)

3.1. Crises de ausência (CA)
3.1.1. Crise de ausência típica (AT)
3.1.2. Crises de ausência atípica (AA)
3.1.3. Crises de ausência mioclônica (MA)
3.1.4. Mioclonia palpebral com / sem ausência (AMP)
3.2. Crise tônico-clônica generalizada (TCG)
3.2.1. Crise mioclônico-tônico-clônica
3.2.2. Crise de ausência para tônico-clônica generalizada
3.3. Outras crises generalizadas**
3.3.1. Crise generalizada mioclônica (GM)
3.3.2. Crise generalizada clônica (GC)
3.3.3. Crise generalizada com mioclonia negativa (GMN)
3.3.4. Espasmo epiléptico generalizado (EEG)
3.3.5. Crise generalizada tônica (GT)
3.3.6. Crise generalizada atônica (GA)
3.3.7. Crise generalizada mioclônico-atônica (GMA)

4. Não classificadas

*Ver Tabela 2 com as características semiológicas.
** Este é um termo de agrupamento, não um conceito definido.

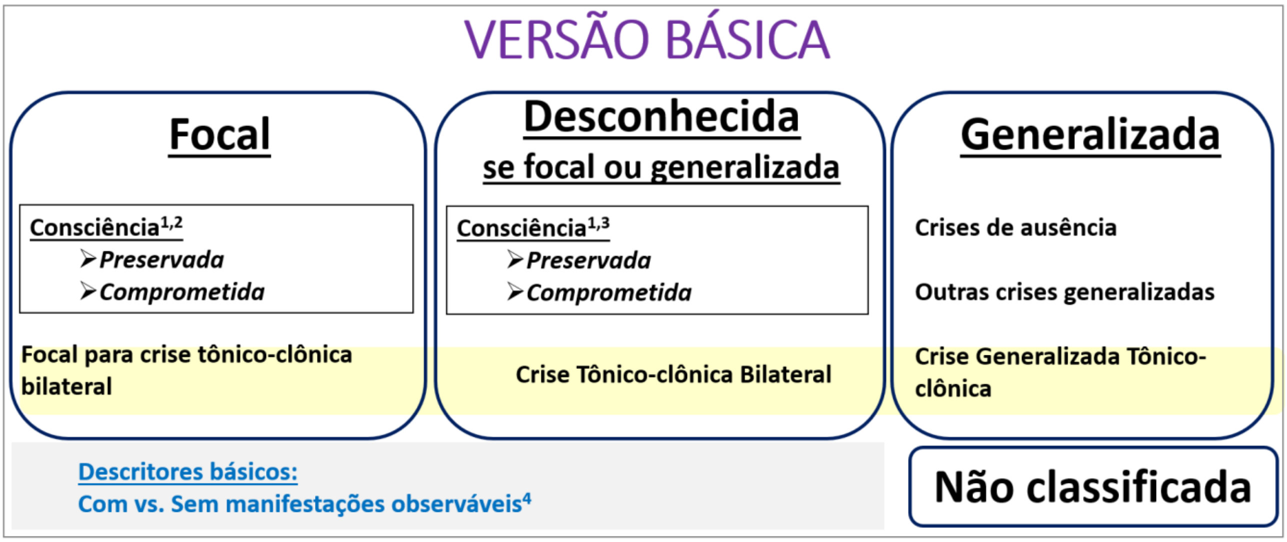

Legenda para a Figura 1

1. Operacionalmente definida por *perceptividade* e *responsividade*.
2. Quando o estado de consciência é desconhecido, classifique como focal (sem especificar a subclassificação)
3. Se o estado de consciência é desconhecido, classifique como desconhecido se focal ou generalizado (sem especificar a subclassificação)
4. Manifestações observáveis são facilmente reconhecíveis por uma testemunha. Estas podem ser motoras, afásicas, autonômicas ou outras (ver Table 2). Comprometimento de consciência deve ser qualificado como uma manifestação observável.

Classificadores (tipos de crises) são mostrados na cor preta, enquanto descritores estão em azul. O fundo horizontal amarelo na figura destaca que as crises tônico-clônicas bilaterais—associadas à maior morbidade e mortalidade—podem ocorrer nas três principais classes de crises.

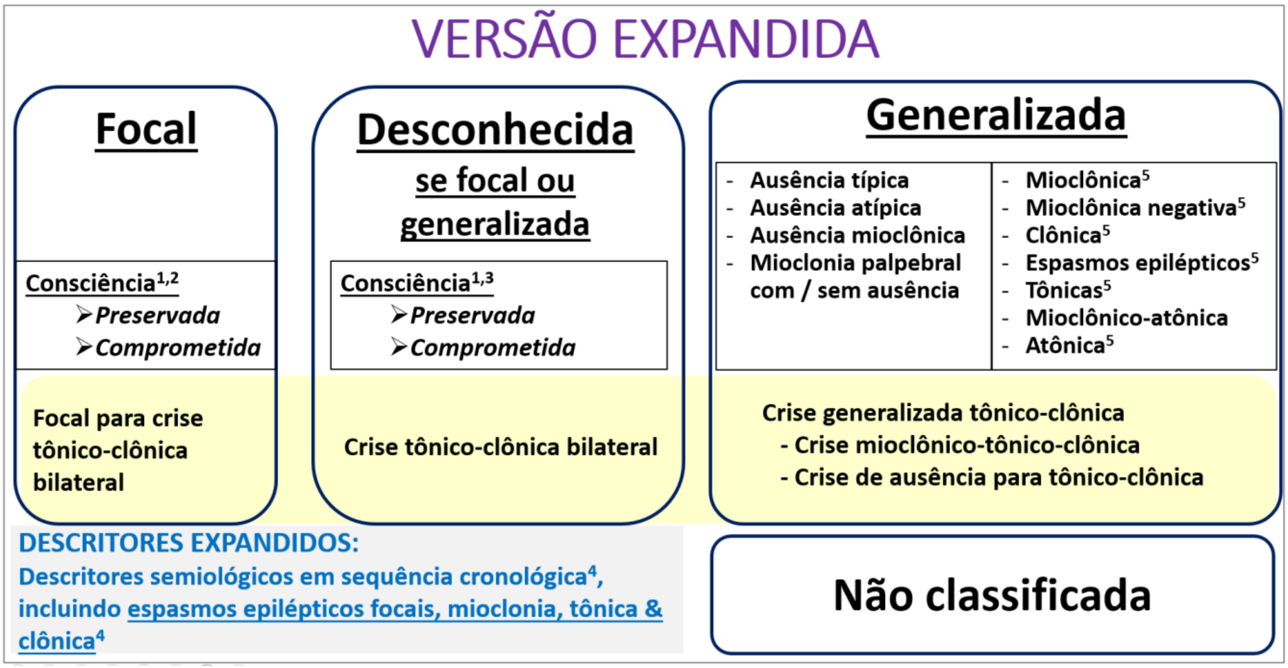

Legenda para a Figura 2

1. Operacionalmente definida por *perceptividade* e *responsividade*.
2. Quando o estado de consciência é desconhecido, classifique como focal (sem especificar a subclassificação)
3. Se o estado de consciência é desconhecido, classifique como desconhecido se focal ou generalizado (sem especificar a subclassificação)
4. Descreva usando os termos ndo glossário de semiologia da ILAE (ver tabela 2).
5. Estes fenômenos podem ocorrer em crises focais (usualmente unilateralmente ou assimetricamente) como parte da semiologia de uma crise focal.

Classificadores (tipos de crises) são mostrados na cor preta, enquanto os descritores estão em azul. O fundo horizontal amarelo na figura destaca que as crises tônico-clônicas bilaterais—associadas à maior morbidade e mortalidade—podem ocorrer nas três principais classes de crises.

Romanian translation

1. Crize Focale (F)
1.1. Crize focale cu conştienţa păstrată (FCP)
1.2. Crize focale cu conştienţa alterată (FCA)
1.3. Crize focale cu evoluţie spre criza tonico-clonică bilaterală (FTCB)

Termeni descriptivi

- **De bază:**
    - Cu manifestări observabile
    - Fără manifestări observabile
- **Detaliaţi:**
    - Termeni descriptivi semiologici în secvenţă cronologică: Semiologie (glosar*) + Modificatori somatotopici

2. Necunoscut dacă sunt focale sau generalizate (U)
2.1. Necunoscut dacă sunt focale sau generalizate - Crize cu conştienţa păstrată (CP)
2.2. Necunoscut dacă sunt focale sau generalizate - Crize cu conştienţa alterată (CA)
2.3. Necunoscut dacă sunt focale sau generalizate - Crize tonico-clonice bilaterale (TCB)

Termeni descriptivi

- **De bază:**
    - Cu manifestări observabile
    - Fără manifestări observabile
- **Detaliaţi:**
    - Termeni descriptivi semiologici în secvenţă cronologică: Semiologie (glosar*) + Modificatori somatotopici

3. Crize Generalizate (G)
3.1. Criză de absenţă (A)
3.1.1. Criză de absenţă tipică (AT)
3.1.2. Criză de absenţă atipică (AA)
3.1.3. Criză de absenţă mioclonică (AM)
3.1.4. Criză cu Mioclonii palpebrale cu / fără absenţă (MPA)
3.2. Criză tonico-clonică generalizată (GTC)
3.2.1. Criză mioclonic-tonic-clonică
3.2.2. Criză de absenţă cu evoluţie spre criza tonico-clonică
3.3. Alte crize generalizate
3.3.1. Criză mioclonică generalizată (MG)
3.3.2. Criză clonică generalizată (CC)
3.3.3. Criză de mioclonus negativ generalizată (MNG)
3.3.4. Spasm epileptic generalizat (SEG)
3.3.5. Criză tonică generalizată (TG)
3.3.6. Criză atonică generalizată (AG)
3.3.7. Criză mioclo-atonică generalizată (MAG)

4. Neclasificate

*Vezi Tabelul 2 pentru caracteristicile semiologice.
Acesta este un termen general, nu un concept definit.

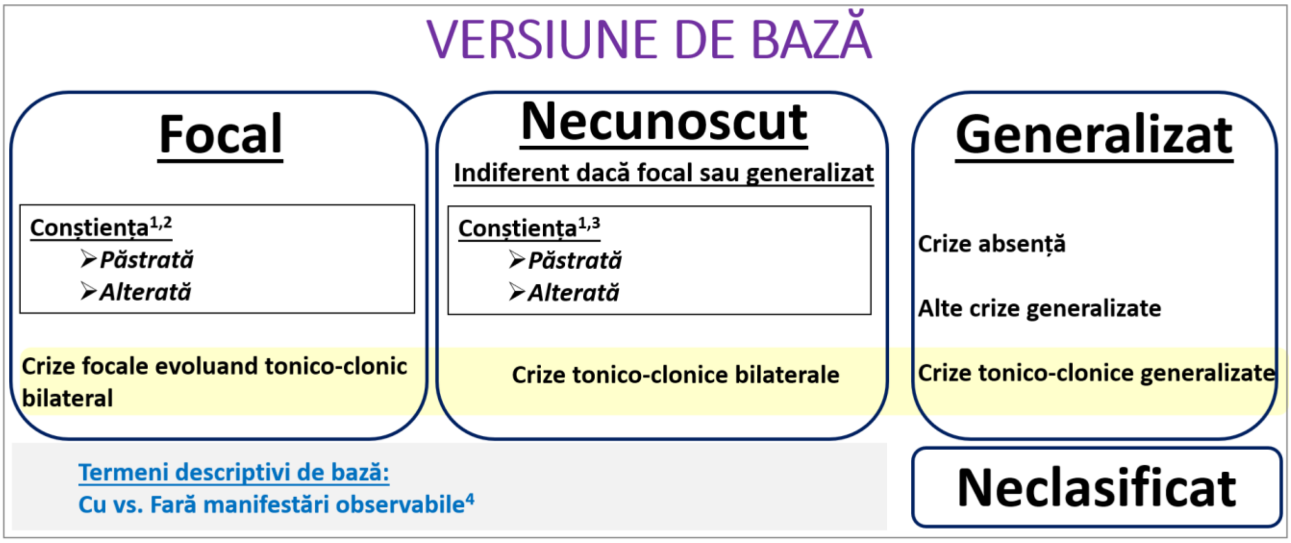

Legendă la Figura 1

1. Definit operațional prin a ști ce se întamplă și prin responsivitate.
2. Cand starea de conștientă este necunoscută, se clasifică FOCAL (fară a specifica subclasificarea).
3. Dacă starea de conștientă este necunoscuta, se clasifică NECUNOSCUT indiferent dacă focal sau generalizat (fară a specifica subclasificarea).
4. Manifestările observabile sunt imediat recunoscute de catre un martor vizual. Acestea pot fi motorii, tulburări de vorbire/exprimare (afazie), vegetative sau altele (vezi Tabelul 2). Afectarea conștienței se califică drept manifestare observabilă.

Tipurile de crize sunt arătate in negru în timp ce descrierea lor este în albastru. Crizele tonico-clonice bilaterale – asociate cu cea mai mare morbiditate și mortalitate - pot apare în toate clasele principale de crize, fapt subliniat de fondul galben orizontal prezent în figuri.

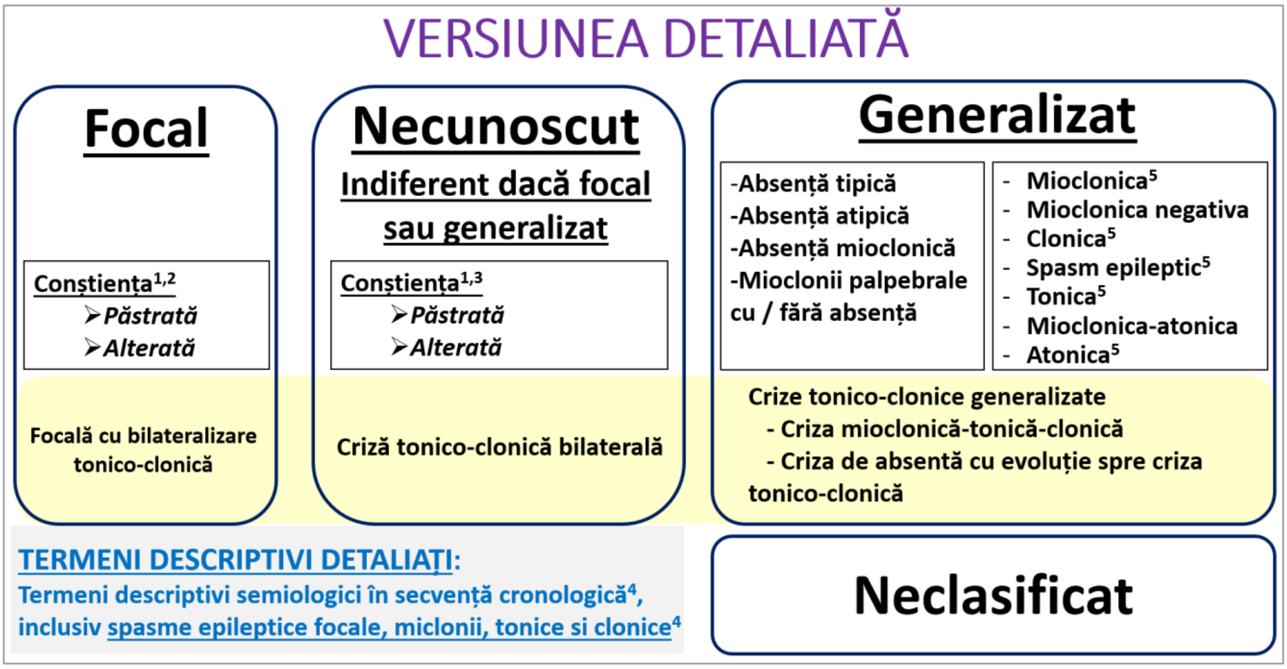

Legenda pentru Figura 2

1. Operațional definit prin a sti ce se intampla și responsivitate
2. Când starea de conștiență este necunoscută, se clasifică drept criză FOCALĂ (fără a specifica sub-clasificarea).
3. Dacă starea de conștiență este necunoscută, se clasifică drept NECUNOSCUT indiferent daca este focală sau generalizată (fără a specifica sub-clasificarea).
4. Semiologia crizei este descrisă folosind termenii din glosarul de semiologie al ILAE (vezi Tabelul 2).
5. Aceste fenomene pot apărea, de asemenea, în crizele focale (de obicei unilateral sau asimetric), ca parte a semiologiei unei crize focale.

Tipurile de crize sunt reprezentate cu text negru, în timp ce temenii descriptivi sunt evidențiați cu text albastru. Fundalul galben orizontal din figuri subliniază faptul că crizele tonico-clonice bilaterale – asociate cu cea mai mare morbiditate și mortalitate – pot apărea în toate cele trei clase principale de crize.

Russian translation

1. Фокальные (Ф)

1.1. Фокальные приступы с сохранным сознанием (ФСС)
1.2. Фокальные приступы с наруценным сознанием (ФНС)
1.3. Фокальные с переходом в билатеральные тонико-клонические приступы (ФБТК)

Дескрипторы

- *Базовые:*
  - *С наблюдаемыми приявлениями*
  - *Без наблюдаемых проявлений*
- *Расширенные:*
  - *Семиологические дескрипторы в хронологической последовательности: Семиология (глоссарий*) + Соматоторические модификаторы*

2. С неизвестным началом, либо фокальные, либо генерализованные (Н)

2.1. С неизвестным началом, либо фокальные, либо генерализованные – Приступы с сохранным сознанием (СС)
2.2. С неизвестным началом, либо фокальные, либо генерализованные – Приступы с нарушенным сознанием (НС)
2.3. С неизвестным началом, либо фокальные, либо генерализованные – Билатерильные тонико-кллоничесике приступы (БТК)

Дескрипторы

- *Базовые:*
  - *С наблюдаемыми приявлениями*
  - *Без наблюдаемых проявлений*
- *Расширенные:*
  - *Семиологические дескрипторы в хронологической последовательности: Семиология (глоссарий*) + Соматоторические модификаторы*

3. Генерализованные (Г)

3.1. Абсансы (А)
 3.1.1. Типичные абсансы (ТА)
 3.1.2. Атипичные абсансы (АА)
 3.1.3. Миоклонические абсансы (МА)
 3.1.4. Миоклонии век с / без абсансов (МВА)
3.2. Генерализованные тонико-клонические приступы (ГТК)
 3.2.1. Миоклонические с переходом в тонико-клонические приступы
 3.2.2. Абсансы с переходом в тонико-клонические приступы
3.3. Другие генерализованные приступы**
 3.3.1. Генерализованные миоклонические приступы (ГМ)
 3.3.2. Генерализованные клонические приступы (ГК)
 3.3.3. Генерализованный негативный миоклонус (ГНМ)
 3.3.4. Генерализованные эпилептические спазмы (ГЭС)
 3.3.5. Генерализованные тонические приступы (ГТ)
 3.3.6. Генерализованные атонические приступы (ГА)
 3.3.7. Генерализованные миоклонически-атонические приступы (ГМА)

4. Неклассифицируемые

*См. Таблицу 2. Семиологические признаки.
**Это – групповой термин, а не определенная концепция.

БАЗОВАЯ ВЕРСИЯ

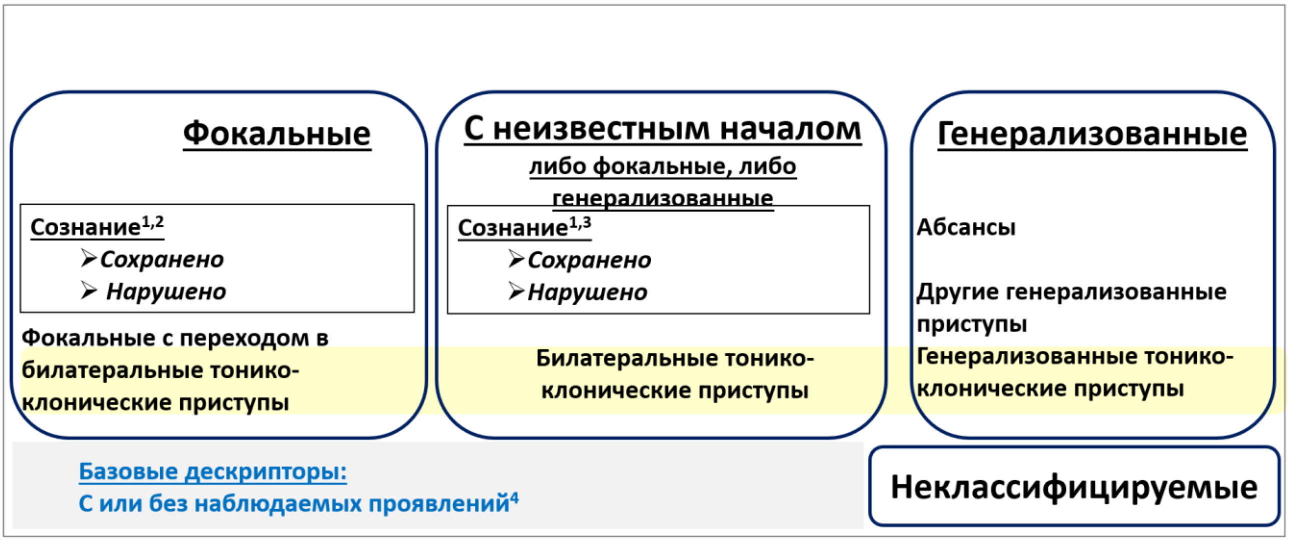

Сноски к Рисунку 1

1. Определяется осознанностью и ответными реакциями.
2. Когда состояние сознания неизвестно, классифицируются как фокальные (без спецификации суб-классификации)
3. Если состояние сознания неизвестно, классифицируются как приступы с неизвестным началом, либо фокальные, либо генерализованные (без спецификации суб-классификации)
4. Наблюдаемые проявления распознаются очевидцем. Они могут быть моторными, афазическими, вегетативными или другими (см. Таблицу семиологии). Нарушенное сознание квалифицируется как наблюдаемое проявление.

Классификаторы (типы приступов) обозначены черным цветом, тогда как дескрипторы – синим цветом. Горизонтальный желтый фон на рисунках подчеркивает, что билатеральные тонико-клонические приступы, сочетающиеся с наиболее высокой заболеваемостью и смертностью, могут наблюдаться при всех трех основных классах приступов.

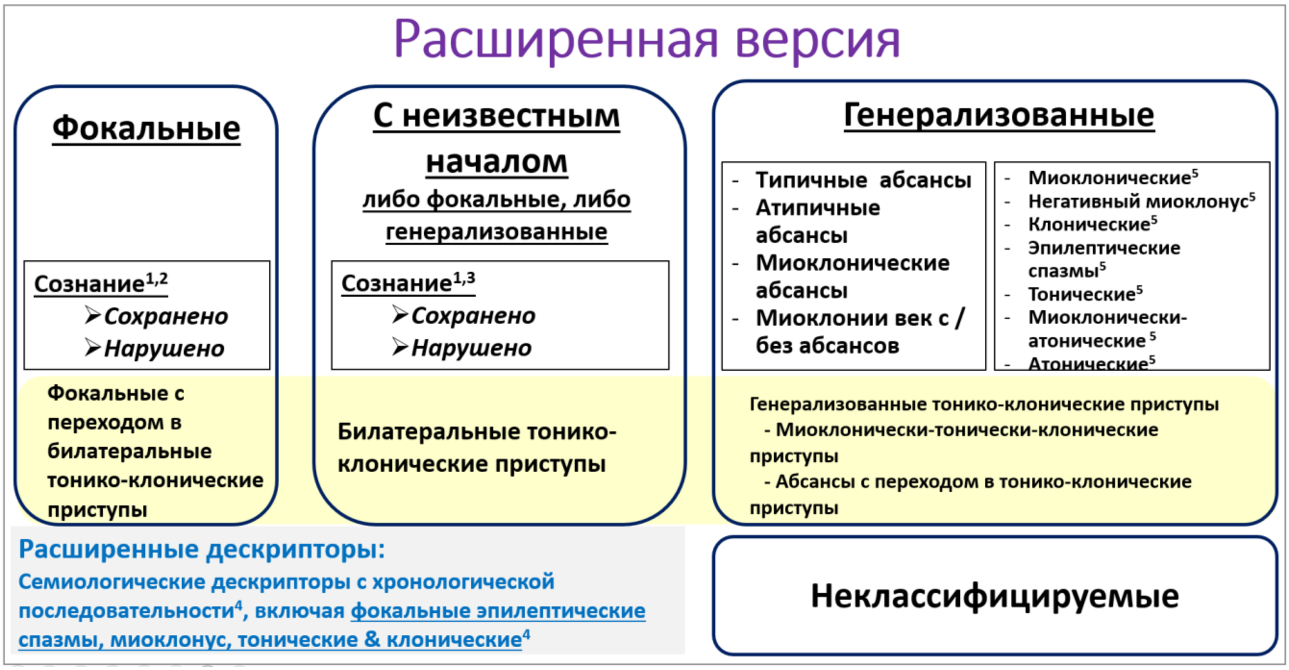

Сноски к Рисунку 2

1. Определяется осознанностью и ответными реакциями.
2. Когда состояние сознания неизвестно, классифицируются как фокальные (без спецификации суб-классификации)
3. Если состояние сознания неизвестно, классифицируются как приступы с неизвестным началом, либо фокальные, либо генерализованные (без спецификации суб-классификации)
4. Описаны с использованием терминов семиологического глоссария ILAE (см. Таблицу 2).
5. Данные феномены могут наблюдаться также при фокальных приступах (обычно односторонне или асимметричные) как элемент семиологии фокального приступа.

Классификаторы (типы приступов) обозначены черным цветом, тогда как дескрипторы – синим цветом.

Горизонтальный желтый фон на рисунках подчеркивает, что билатеральные тонико-клонические приступы, сочетающиеся с наиболее высокой заболеваемостью и смертностью, могут наблюдаться при всех трех основных классах приступов.

Spanish translation

1. Focal (F)

1.1. Crisis Focal con la Consciencia Preservada (FCP)
1.2. Crisis Focal con la Consciencia Alterada (FCA)
1.3. Crisis Focal a Tónico-Clónica (FTC)

Descriptores

- *Básicos:*
  - *Con manifestaciones observables*
  - *Sin manifestaciones observables*
- *Expandidos:*
  - *Descriptores semiológicos presentados en una secuencia cronológica: Semiología (glosario*) + Modificadores somatotópicos*

2. Desconocido si son focales o generalizadas (D)

2.1. Desconocido si son focales o generalizadas – Crisis con la consciencia conservada (CP)
2.2. Desconocido si son focales o generalizadas – Crisis con la consciencia alterada (CA)
2.3. Desconocido si son focales o generalizadas – Crisis tónico-clónica bilateral (TCB)

Descriptores

- *Básicos:*
  - *Con manifestaciones observables*
  - *Sin manifestaciones observables*
- *Expandidos:*
  - *Descriptores semiológicos presentados en una secuencia cronológica: Semiología (glosario*) + Modificadores somatotópicos*

3. Generalizada (G)

3.1. Crisis de ausencias (CA)
- 3.1.1. Crisis de ausencia típica (AT)
- 3.1.2. Crisis de ausencia atípica (AA)
- 3.1.3. Crisis de ausencia mioclónica (AM)
- 3.1.4. Crisis de mioclonías palpebrales con o sin ausencia (MPA)

3.2. Crisis tónico-clónica generalizada (TCG)
- 3.2.1. Crisis mioclónica-tónico-clónica
- 3.2.2. Crisis de ausencia a tónico-clónica

3.3. Otras crisis generalizadas**
- 3.3.1. Crisis mioclónica generalizada (MG)
- 3.3.2. Crisis clónica generalizada (CG)
- 3.3.3. Crisis mioclónica negativa generalizada (MNG)
- 3.3.4. Espasmo epiléptico generalizado (EEG)
- 3.3.5. Crisis tónica generalizada (TG)
- 3.3.6. Crisis atónica generalizada (AG)
- 3.3.7. Crisis mioclónica y atónica generalizada (MAG)

4. No clasificada

*Véase la Tabla 2 para las características de la semiología.
**Este es un término de agrupación, no un concepto definido.

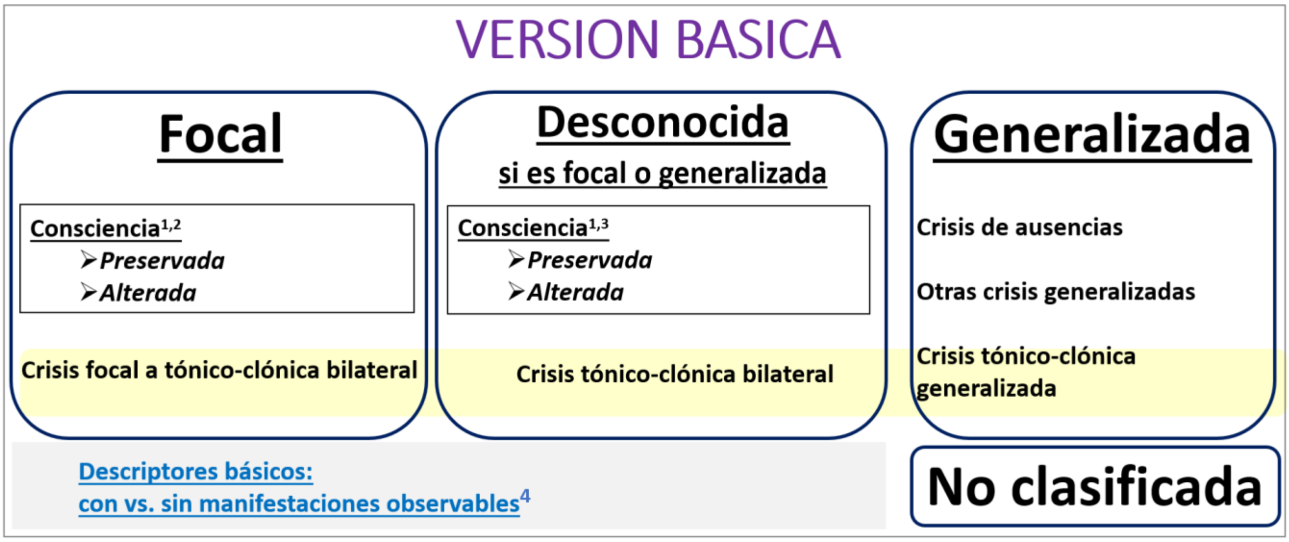

Leyenda de la Figura 1:

1. Definido operativamente por consciencia y capacidad de respuesta.
2. Cuando se desconoce el estado de consciencia, clasificar como focal (sin especificar la subclasificación).
3. Si se desconoce el estado de consciencia, clasificar como desconocido si es focal o generalizado (sin especificar la subclasificación).
4. Las manifestaciones observables son fácilmente reconocibles por un testigo presencial. Pueden ser motoras, afásicas, autonómicas o de otro tipo (véase la Tabla 2). La alteración de la consciencia se considera una manifestación observable.

Los clasificadores (tipos de crisis) se muestran en negro, mientras que los descriptores se muestran en color azul. El fondo amarillo horizontal de las figuras resalta que las crisis tónico-clónicas bilaterales -asociadas con la mayor morbilidad y mortalidad- pueden ocurrir en las tres clases principales de crisis.

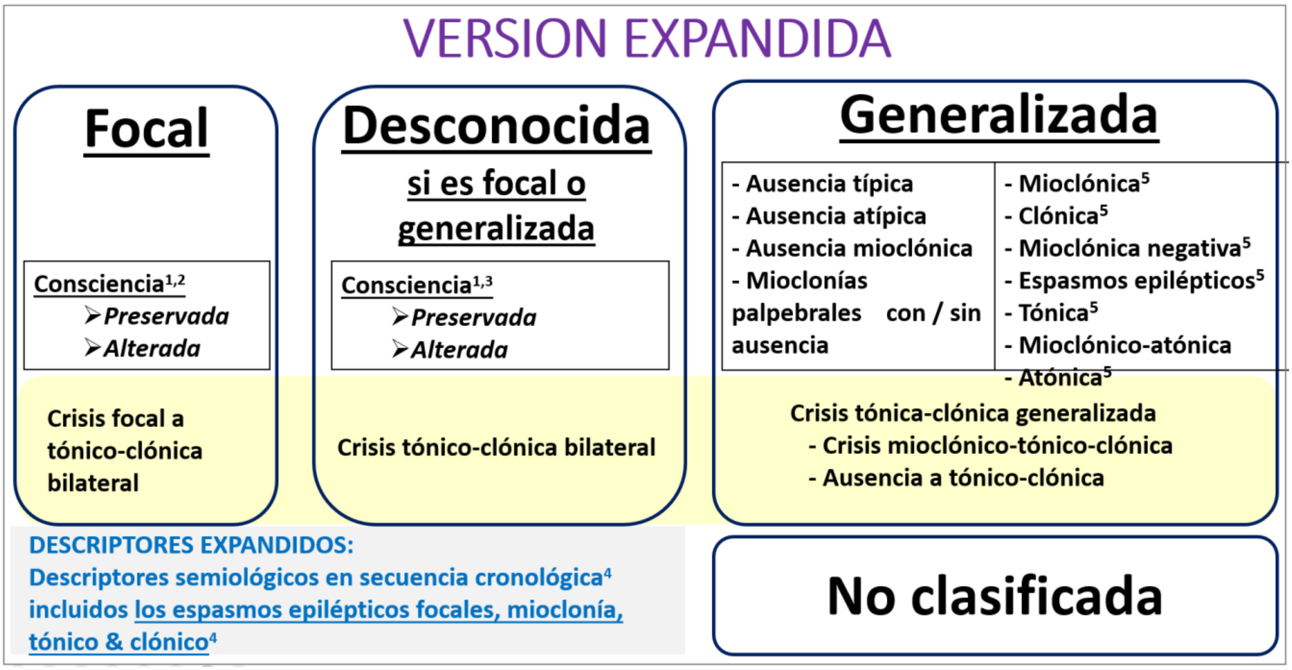

Leyenda de la Figura 2:

1. Definido operativamente por consciencia y capacidad de respuesta.
2. Cuando se desconoce el estado de consciencia, clasificar como focal (sin especificar la subclasificación)
3. Si se desconoce el estado de consciencia, clasificar como desconocido si es focal o generalizado (sin especificar la subclasificación)
4. Descritos utilizando los términos del glosario de semiología de la ILAE (ver Tabla 2).
5. Estos fenómenos pueden ocurrir también en las crisis focales (generalmente de forma unilateral o asimétrica) como parte de la semiología de una crisis focal.

Los clasificadores (tipos de crisis) se muestran en negro, mientras que los descriptores se muestran en color azul. El fondo amarillo horizontal en las figuras destaca que las crisis tónico-clónicas bilaterales -asociadas con la mayor morbilidad y mortalidad- pueden ocurrir en las tres clases principales de crisis.

Ukrainian translation

1. Фокальні (Ф)

1.1. Фокальні зі збереженою свідомістю (ФЗС)
1.2. Фокальні з порушеною свідомістю (ФПС)
1.3. Фокальні з переходом в білатеральні тоніко-клонічні (*білатеральні тоніко-клонічні з фокальним початком*) (ФБТК)

Дескриптори (Визначення)
- *Базисні:*
  - *З видимими проявами*
  - *Без видимих проявів*
- *Розширені:*
  - *Дескриптори семіології в хронологічній послідовності: Семіологія (глосарій*) + Соматотопічні модифікатори*

2. Невизначено фокальні чи генералізовані (Н)

2.1. Невизначено фокальні чи генералізовані зі збереженою свідомістю (ЗС)
2.2. Невизначено фокальні чи генералізовані з порушеною (ПС)
2.3. Невизначено фокальні чи генералізовані з переходом в білатеральні тоніко-клонічні (БТК)

Дескриптори (Визначення)
- *Базисні:*
  - *З видимими проявами*
  - *Без видимих проявів*
- *Розширені:*
  - *Дескриптори семіології в хронологічній послідовності: Семіологія (глосарій*) + Соматотопічні модифікатори*

3. Генералізовані (Г)

3.1. Абсанси (А)
- 3.1.1. Типові абсанси (ТА)
- 3.1.2. Атипові абсанси (АА)
- 3.1.3. Міоклонічні абсанси (МА)
- 3.1.4. Міоклонія повік з/без абсансу (МПА)

3.2. Генералізовані тоніко-клонічні (ГТК)
- 3.2.1. Міоклонічні тоніко-клонічні
- 3.2.2. Абсанси з переходом в тоніко-клонічні

3.3. Інші генералізовані**
- 3.3.1. Генералізовані міоклонічні (ГМ)
- 3.3.2. Генералізовані клонічні (ГК)
- 3.3.3. Генералізовані негативні міоклонуси (ГНМ)
- 3.3.4. Генералізовані епілептичні спазми (ГЕМ)
- 3.3.5. Генералізовані тонічні (ГТ)
- 3.3.6. Генералізовані атонічні (ГА)
- 3.3.7. Генералізовані міоклоніко-атонічні (ГМА)

4. Некласифіковані

* Див. Таблицю 2 з семіологічними характеристиками.
** Це груповий термін, а не визначене поняття.

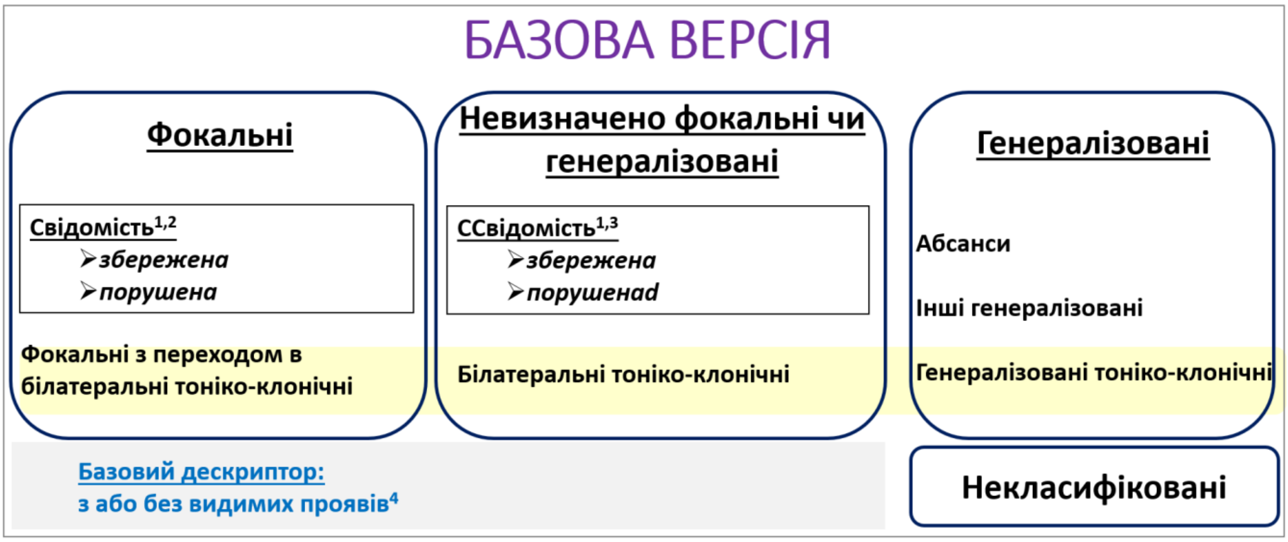

Позначення до рисунка 1

1. Оперативно визначається усвідомленістю та аналізом відчуттів.
2. Коли стан свідомості невідомий, класифікувати як вогнищевий (без уточнення підкласифікації)
3. Якщо стан свідомості невідомий, класифікуйте як невідомий, вогнищевий чи генералізований (без уточнення підкласифікації)

4 Спостережувані прояви легко розпізнаються очевидцем. Вони можуть бути моторними, афатичними, вегетативними або іншими (див. таблицю 2). Порушення свідомості кваліфікується як спостережуваний прояв.

Класифікатори (типи нападів) показані чорним кольором, а дескриптори – синім. Горизонтальний жовтий фон на малюнках підкреслює, що двобічні тоніко-клонічні напади, пов'язані з найвищою захворюваністю та смертністю, можуть виникати в усіх трьох основних класах нападів.

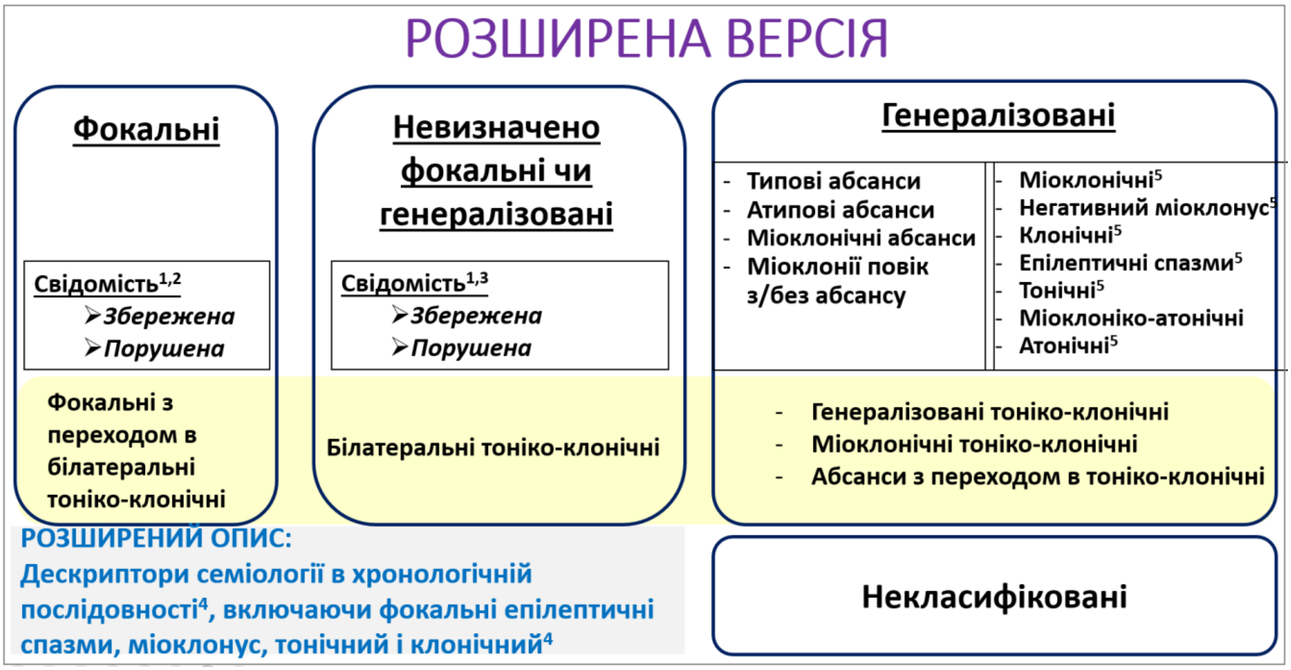

Позначення до рисунка 2

1. Оперативно визначається усвідомленістю та аналізом відчуттів
2. Коли стан свідомості невідомий, класифікувати як вогнищевий (без уточнення підкласифікації)
3. Якщо стан свідомості невідомий, класифікуйте як невідомий, вогнищевий чи генералізований (без уточнення підкласифікації)
4. Описано з використанням термінів у семіологічному глосарії ILAE (див. таблицю 2).
5. Ці явища можуть виникати також у фокальних нападах (зазвичай односторонніх або асиметричних) як частина симптомів фокального нападу.

Класифікатори (типи нападів) показані чорним кольором, а дескриптори – синім. Горизонтальний жовтий фон на малюнках підкреслює, що двобічні тоніко-клонічні напади, пов’язані з найвищою захворюваністю та смертністю, можуть виникати в усіх трьох основних класах нападів.
