# Supplementary figures and images for: Updated classification of epileptic seizures: Position paper of the International League Against Epilepsy
Source: Epilepsia. 2025 Apr 23;66(6):1804–23. doi: 10.1111/epi.18338 (PMC12169392; doi:10.1111/epi.18338)

## Slide 1
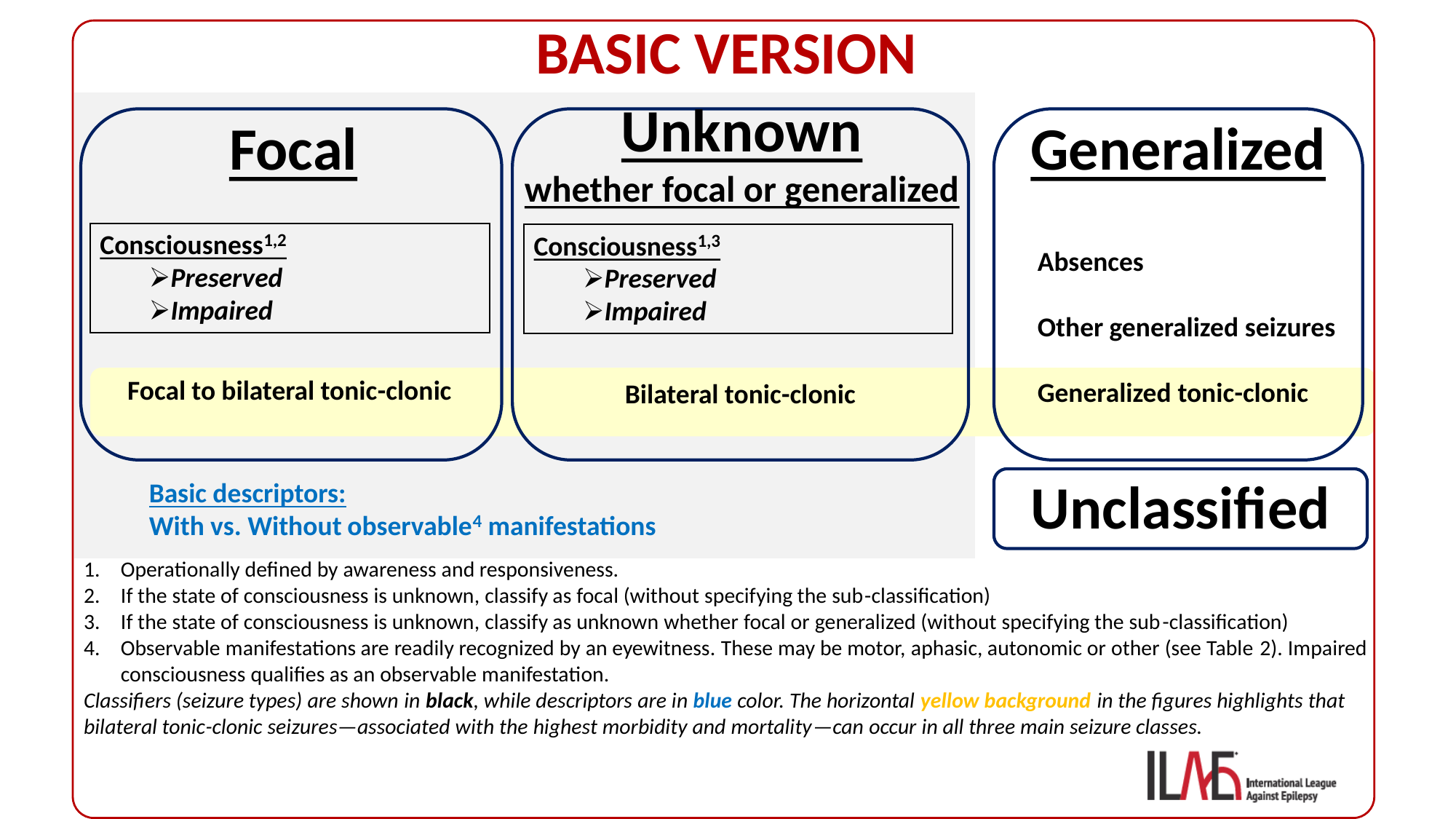

## Slide 2
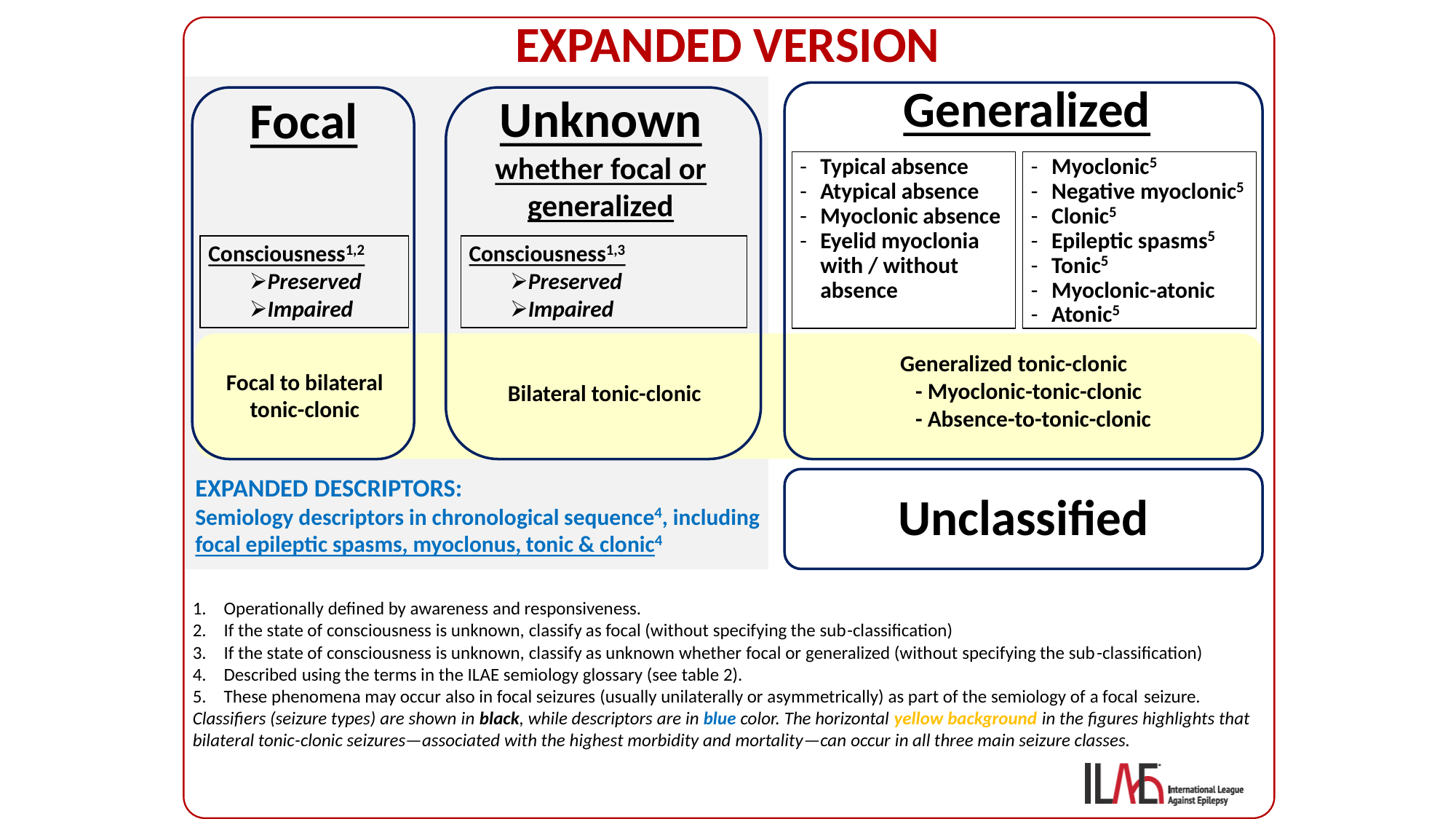

## Slide 3
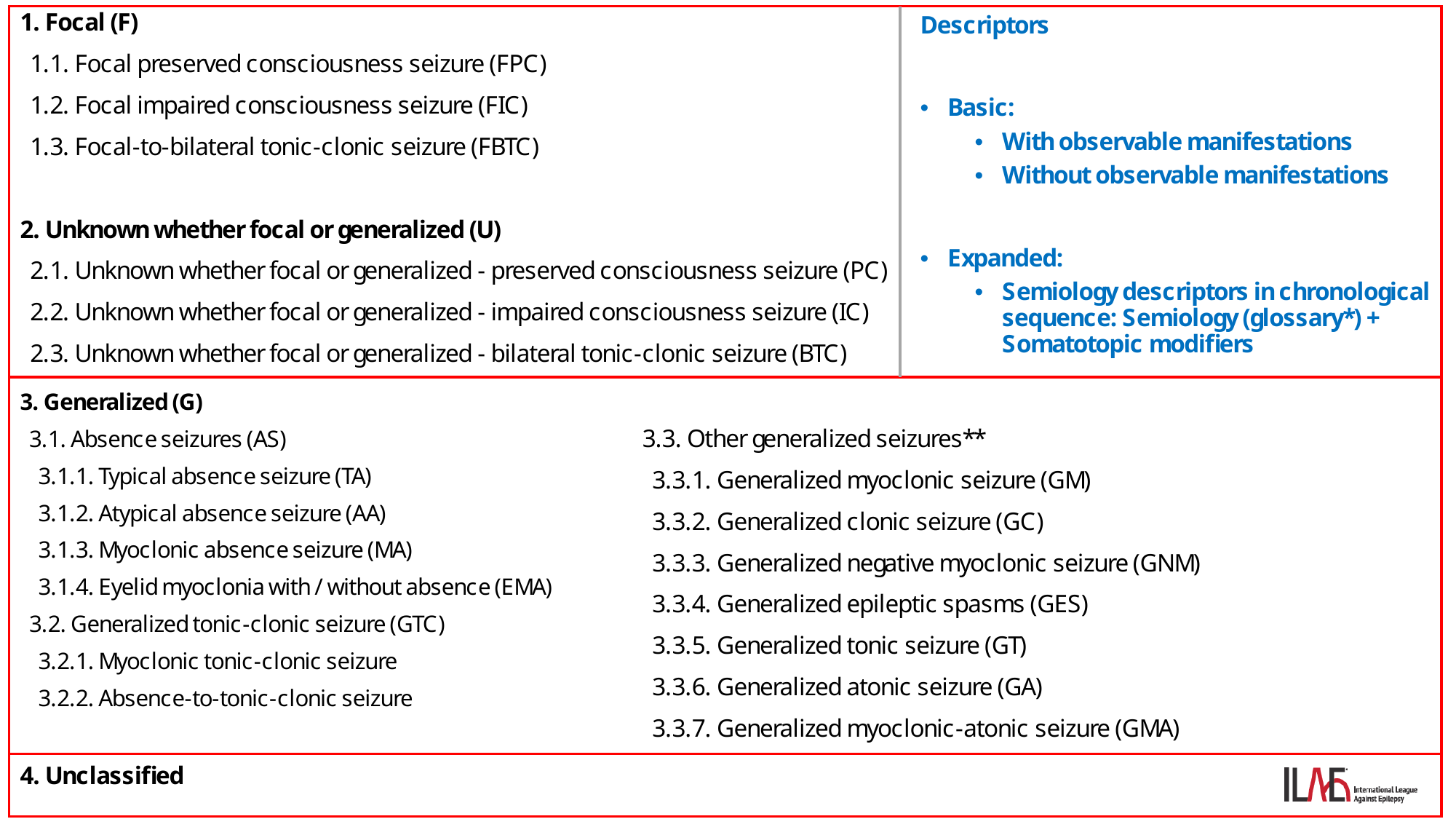

## Slide 4
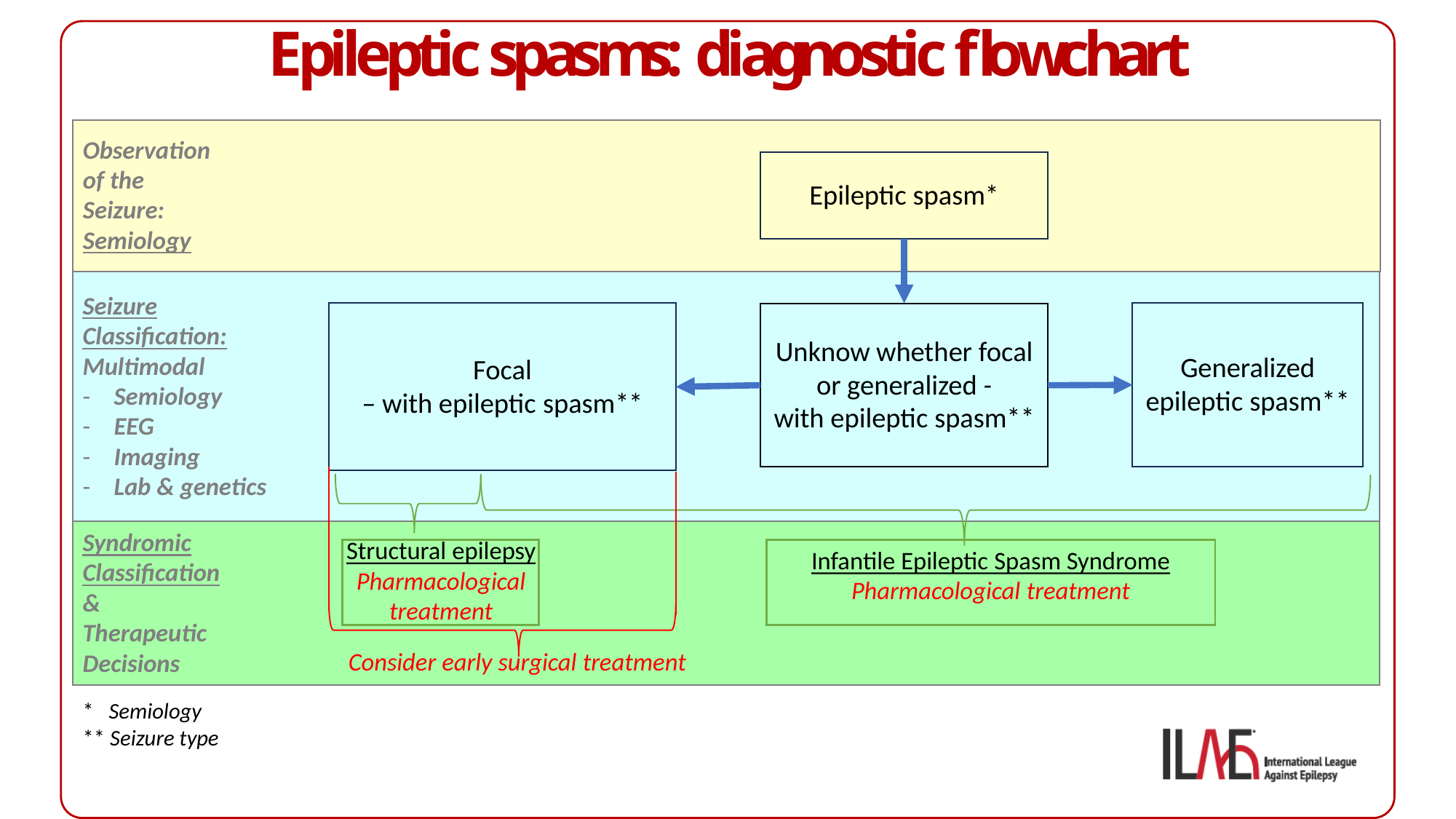

Supplement: Supplementary file 7 — Data S7. [file EPI-66-1804-s006.pptx]
